# PKIB facilitates bladder cancer proliferation and metastasis through mediation of HSP27 phosphorylation by PKA

**DOI:** 10.1038/s41419-025-07814-7

**Published:** 2025-07-01

**Authors:** Xiaolong Liu, Xiaoyu Yin, Feng Yuan, Shiqing Li, Fei Xiao, Feng Zhou, Xudong Pan, Yatfaat Ho, Shuo Dong, Duan Xu, Yunqing Ma, Zhengding Cao, Zhe Lei, Yi Sun

**Affiliations:** 1 Department of Urology, The Second Affiliated Hospital of Soochow University, Suzhou, 215004 China; 2 Department of Genetics, School of Basic Medical Sciences, Suzhou Medical College of Soochow University, Suzhou, 215123 China; 3 Department of Urology, The First Affiliated Hospital of Soochow University, Suzhou, 215006 China; 4 Department of Bioinformatics and Computational Biology, School of Life Sciences, Suzhou Medical College of Soochow University, Suzhou, 215123 China; 5 Department of General Medicine, The First Affiliated Hospital of Soochow University, Suzhou, 215006 China; 6 Department of Urology, Suzhou BenQ Medical Center, Suzhou, 215000 China; 7 Institute of Epidemiology & Health Care, Faculty of Population Health Sciences, University College London, London, WC1E 6BT UK; 8 Department of Pathology, The First Affiliated Hospital of Soochow University, Suzhou, 215006 China; 9 Clinical Medicine Research Institute of Soochow University and Suzhou BenQ Medical Center, Suzhou Medical College of Soochow University, Suzhou, 215123 China

**Keywords:** Bladder cancer, Kinases

## Abstract

Cyclic AMP-dependent protein kinase A (PKA) is recognized for its pivotal involvement in various cancer types, with Protein Kinase Inhibitor Beta (PKIB) serving as an endogenous inhibitor that curtails PKA activity. Despite the documented escalation of *PKIB* expression in several malignancies, a comprehensive understanding of its precise mechanistic implications in human cancers remains elusive. This investigation is centered on bladder cancer (BLCA), unveiling an augmented expression of *PKIB* concomitant with heightened BLCA cell proliferation, migration, and invasion in vitro and augmented tumorigenic potential in an in vivo model. Mechanistically, PKIB disrupts PKA kinase activity, thereby resulting in diminished phosphorylation of the substrate target protein HSP27 at serine 15, 78, and 82. Additionally, the transcription factor MYCN exhibits an affinity for the *PKIB* promoter, leading to its enhanced expression in the context of BLCA. These findings reveal the oncogenic proclivity of PKIB and introduce a novel signalling pathway in BLCA, providing valuable insights into potential therapeutic targets for precise intervention.

## Introduction

Cell growth is regulated by signal transduction pathways, and their dysregulation can contribute to cancer. Post-translational modifications such as phosphorylation, ubiquitination, acetylation, and methylation are key to controlling these pathways. Abnormal modifications can drive tumorigenesis by disrupting cell signaling [1]. Since kinase/phosphatase signaling dysregulation is common in tumors, pinpointing specific signaling interactions is crucial for developing targeted cancer therapies [2]. Cyclic AMP–dependent protein kinase A (PKA) is a versatile enzyme that plays a crucial role in various signaling pathways, influencing both normal physiological processes and disease states, including cancer [3]. PKA functions as a tetramer made up of two regulatory (R) and two catalytic (C) subunits. When cyclic AMP (cAMP) binds to the regulatory subunits, it triggers the release and activation of the catalytic subunits, which then phosphorylate serine and threonine residues on target proteins [4]. PKA has been demonstrated to promote cell migration and invasion, enhancing signal transduction and facilitating early cell cycle progression [5–7]. PKA has attracted extensive attention in cancer research [8]; however, its role in in tumorigenesis is complex and context-dependent, varying according to cancer types, stages, and other factors [9]. The cAMP/PKA signaling pathway is implicated in various facets of malignancy, including invasion, migration, adhesion, and clonal development, within a range of cancers encompassing glioblastoma, ovarian, colorectal, breast cancer, esophageal squamous cell carcinoma, and pituitary tumors [10–15]. Nevertheless, PKA has also demonstrated tumor-suppressive effects in certain cancers. Research indicates that PKA exhibits tumor-suppressive properties in glioblastoma cells [16–18]. In the context of lung cancer, PKA enhances apoptosis induced by radiotherapy by promoting phosphorylation of PP2A and upregulating *AP1* expression [19, 20]. The spatiotemporal specificity of PKA is exquisitely controlled, particularly by the A-kinase anchoring proteins (AKAPs), the regulatory subunits of PKA, and the endogenously expressed PKA inhibitory peptides (PKIs) [21]. Nevertheless, our understanding of PKA signaling merely skims the surface of an extremely intricate phosphorylation network. The substrate specificity of PKA kinases is considerably more diverse than expected and is driven extensively by negative selectivity [22]. Consequently, targeting PKA signaling through specific inhibitors is essential for both cancer therapy and research endeavors. Such interventions could profoundly enhance our understanding of the intricate mechanisms governing downstream signaling pathways associated with PKA [23]. Despite the structural characterization of the PKA catalytic subunit being established for over three decades, the market for PKA inhibitors remains constrained, with no clinical approvals to date [24].

PKIB, denoted as cAMP-dependent protein kinase inhibitor β, is an integral part of the PKIs class, composed of endogenous peptides with the ability to suppress PKA kinase activities [25]. These inhibitors, including the α, β, and γ isomers, engage with PKA’s catalytic subunit via an unphosphorylated pseudosubstrate sequence at the N-terminus, consequently inhibiting PKA [26, 27]. PKIA (PKI-α) is a specific PKA inhibitor extensively researched for its binding and inhibitory capabilities [28]. Notably, PKIB (PKI-β) shares only 40% amino acid identity with PKIA and demonstrates more cell- and tissue-specific expression patterns [23]. Since its identification in rat testis in 1977, the majority of our current understanding regarding the function of PKIB is derived from its homologous protein PKIA; however, its exact functional role remains largely underexplored. A few studies have reported the abnormal overexpression of *PKIB* in a range of cancers, suggesting its potential role in tumorigenesis. In prostate cancer (PC), *PKIB* is notably overexpressed in castration-resistant and aggressive subtypes, where it enhances cell growth and invasion by increasing the kinase activity of PKA-C and the phosphorylation of AKT [29]. Similarly, in non-small cell lung cancer (NSCLC), *PKIB* is upregulated, leading to increased cell proliferation and tumorigenesis through the activation of the PI3K/AKT pathway [30]. PKIB also promotes the proliferation of highly metastatic osteosarcoma cells via the AKT pathway [31]. Additionally, elevated *PKIB* expression and its clinical significance have been identified in triple-negative breast cancer [32–34] and colorectal carcinoma [35]. Notably, a correlation has been observed between *PKIB* expression and AKT phosphorylation levels, as well as between AKT pathway activation and the effects of PKIB in these tumors. These evidences imply a significant potential oncogenic function of PKIB in multiple cancer types. Nevertheless, despite the aforementioned reports suggesting that PKIB might have a role in tumor progression by modifying the kinase activity of PKA-C and altering AKT phosphorylation, as of now, there is no proof that AKT is a substrate for PKA. The exact mechanism through which PKIB governs tumor progression remains unclear, especially the direct substrate of PKA kinase in tumor malignant progression mediated by PKIB and the overexpression regulation of *PKIB* in cancer cells, all of which demand further exploration.

Bladder cancer (BLCA) ranks as the most commonly diagnosed cancer of the urinary system. Recent trends indicate a gradual increase in both its incidence and mortality rates, with approximately 573,000 new cases and 213,000 deaths occurring annually [36]. BLCA is categorized based on the depth of invasion into two main types: muscle-invasive bladder cancer (MIBC) and non-muscle-invasive bladder cancer (NMIBC). Among these subtypes, MIBC has a high degree of malignancy, strong invasive ability, and rapid clinical progression [37]. Despite recent advances in surgical treatment and chemical diagnosis, the effectiveness and specificity of treatments for BLCA remain limited, leading to frequent patient recurrence and disease progression [38]. The 5-year overall survival (OS) rate for patients with MIBC ranges from approximately 60% to 70%. Approximately 10% of patients with BLCA present with disease beyond the bladder, with an associated 5-year OS rate of 5–30% [39]. BLCA is the most underfunded of the most common cancers, which stifled research and contributed to a limited understanding of bladder tumor biology, ultimately led to insufficient progress in treatment [39]. Consequently, advancing research into the molecular mechanisms underlying bladder tumor development and malignant progression is essential. Such research is critical for the identification of novel therapeutic targets and the development of more effective diagnostic and therapeutic strategies for BLCA.

Current evidence suggests that PKA may play a pivotal role in the regulation of BLCA cell invasion, although the findings are currently sporadic. The PKA activator cholera toxin has been observed to specifically inhibit cell proliferation and induce G1 phase arrest in human bladder transitional cell carcinoma (TCC) cells, with these effects being reversed by the PKA inhibitor KT5720 [40]. Additionally, the cAMP/PKA signaling pathway has been shown to impede BLCA cell invasion through the modulation of MAP4-dependent microtubule dynamics [41]. There is evidence that PKA signaling may also mediate the anti-cancer effects of garlic extract in BLCA prevention [42]. Moreover, PKA inhibition has been linked to the attenuation of cAMP-induced reductions in BLCA cell mobility in animal models [43]. These results suggested that the inhibition of PKA kinase is strongly related to the pathogenesis and progression of BLCA. Given the role of PKIs in regulating PKA kinase activity, the study of PKIs may help to reveal the specific functional mechanisms of PKA in tumors. However, the function of PKIs (including PKIA, PKIB, and PKIC) in regulating BLCA progression is currently unclear.

In this study, we elucidated the critical roles and molecular mechanisms of the PKIB as a tumor promoter in BLCA. Our findings indicate that PKIB facilitates a reduction in the phosphorylation of the novel substrate protein HSP27 by inhibiting PKA kinase activity, thereby promoting tumor cell proliferation and migration. Furthermore, the transcription factor MYCN is implicated in regulating the overexpression of *PKIB*. These results illuminate the oncogenic role of PKIB and reveal an innovative signaling axis involving MYCN-PKIB-PKA-HSP27 in BLCA, enhancing our understanding of the intricate cellular pathways and regulatory mechanisms mediated by PKA kinases while providing valuable insights into PKIB as a potential novel therapeutic target for targeted intervention in bladder tumors.

## Results

### *PKIB* is highly expressed in BLCA, and its high expression is associated with BLCA progression

**Fig. 1:**
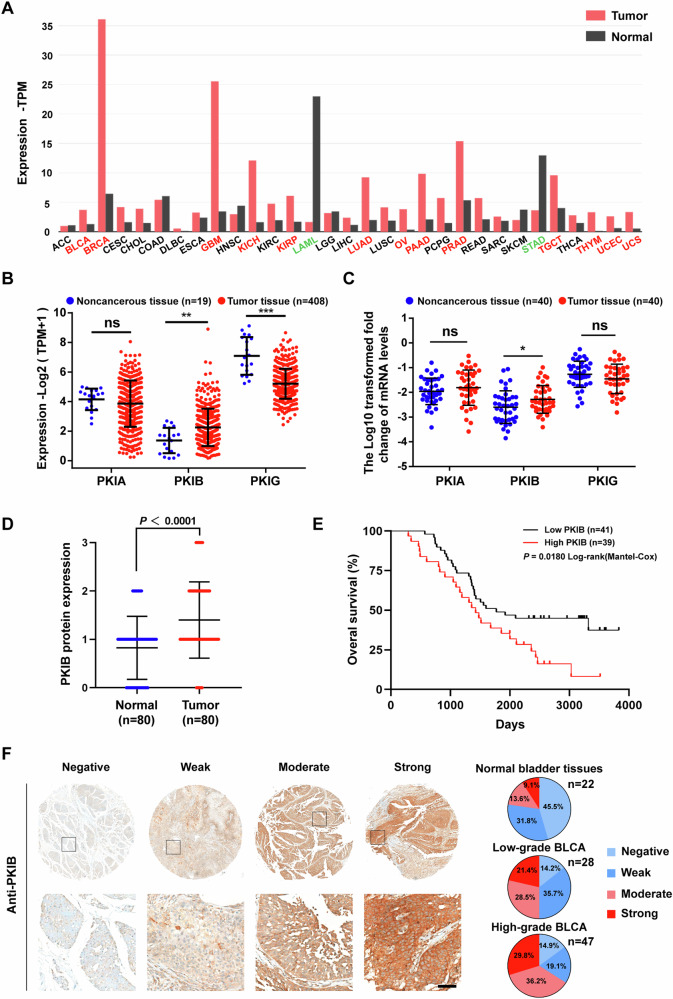
*PKIB* expression is associated with BLCA progression. **A** The expression of *PKIB* in tumor samples was analysed via GEPIA2 (http://gepia2.cancer-pku.cn/#general). The samples highlighted in red and green indicate significant upregulation and downregulation of PKIB, respectively. Adrenocortical carcinoma (ACC), bladder urothelial carcinoma (BLCA), breast invasive carcinoma (BRCA), cervical squamous cell carcinoma and endocervical adenocarcinoma (CESC), cholangiocarcinoma (CHOL), colon adenocarcinoma (COAD), diffuse large B-cell lymphoma (DLBC), esophageal carcinoma (ESCA), glioblastoma multiforme (GBM), head and neck squamous cell carcinoma (HNSC), kidney chromophobe (KICH), kidney renal clear cell carcinoma (KIRC), kidney renal papillary cell carcinoma (KIRP), acute myeloid leukemia (LAML), brain lower grade glioma (LGG), liver hepatocellular carcinoma (LIHC), lung adenocarcinoma (LUAD), lung squamous cell carcinoma (LUSC), ovarian serous cystadenocarcinoma (OV), pancreatic adenocarcinoma (PAAD), pheochromocytoma and paraganglioma (PCPG), prostate adenocarcinoma (PRAD), rectum adenocarcinoma (READ), sarcoma (SARC), skin cutaneous melanoma (SKCM), stomach adenocarcinoma (STAD), testicular germ cell tumors (TGCT), thyroid carcinoma (THCA), thymoma (THYM), and uterine corpus cavernosum (UCEC). **B** mRNA expression analysis of *PKIA*, *PKIB* and *PKIG* in BLCA (*n* = 408) and normal tissue samples (*n* = 19) from the TCGA database. ** *P* = 0.0027, *** *P* < 0.0001, ns = not significant according to unpaired Student’s *t* test. **C** qRT‒PCR analysis of *PKIA*, *PKIB* and *PKIG* mRNA expression in BLCA and matched paracarcinoma tissues (*n* = 40). * *P* = 0.0264, ns = not significant according to unpaired Student’s *t* test. **D** Quantification of PKIB expression in 80 paired BLCA tissues and normal tissues by IHC analysis. *P* < 0.0001 according to unpaired Student’s *t* test. **E** K-M survival analysis of overall survival of patients with BLCA with low (staining intensity ≤1) versus high (staining intensity ≥2) PKIB expression. **F** Representative immunohistochemistry (IHC) images of PKIB-stained normal (*n* = 22) and different grade BLCA tissues (*n* = 75) are shown (left panel). Differential percentage of high-PKIB samples in normal and various BLCA tissues (right panel).

As the role of PKIB in tumors has not been determined, we investigated *PKIB* expression in public human cancer databases using Gene Expression Profiling Interaction Analysis 2 (http://gepia2.cancer-pku.cn). *PKIB* mRNA is overexpressed in 13 out of 31 cancer types, including bladder urothelial carcinoma, breast invasive carcinoma, glioblastoma multiforme, kidney chromophobe, prostate adenocarcinoma, etc. However, it is downregulated in two different tumors, acute myeloid leukemia and stomach adenocarcinoma (Fig. 1A), indicating that PKIB might perform different biological functions depending on the tumor background. We then focused on the expression of *PKIB* in BLCA tissues. It has been reported that endogenous PKA inhibitors (PKIs) contain PKIA, PKIB and PKIG [44]. To evaluate the possible roles of different PKIs in BLCA, we assessed the mRNA levels of the three genes in The Cancer Genome Atlas (TCGA) database (Fig. 1B) and 40 pairs of BLCA samples (Table S2). Intriguingly, only *PKIB* exhibited significant upregulation in both the TCGA database and our clinical specimens (Fig. 1C). Moreover, higher protein level of PKIB were detected in BLCA tissues than in normal tissues (Fig. 1D and Table S3). Kaplan-Meier Survival Analysis demonstrated a significant positive correlation between PKIB overexpression and reduced overall survival (OS) in patients with BLCA (Fig. 1E and Table S3).

To determine the clinical significance of PKIB overexpression in BLCA, immunohistochemistry (IHC) was performed on a tissue array containing 22 normal bladder tissue samples, 28 low-grade BLCA tissue samples and 47 high-grade BLCA tissue samples (Table S4). As shown in Fig. 1F, the percentages of patients with strong and moderate PKIB expression in normal urothelial tissues, low-grade BLCA tissues and high-grade BLCA tissues were 22.7%, 49.9% and 66%, respectively. The expression of PKIB increased significantly (Chi-square test, *P* = 0.0036) with aggressiveness and was highest in high-grade BLCA tissues. Collectively, these results suggest that a substantial elevation in PKIB may significantly contribute to the progression of BLCA.

### PKIB promotes BLCA cell viability and invasion

**Fig. 2:**
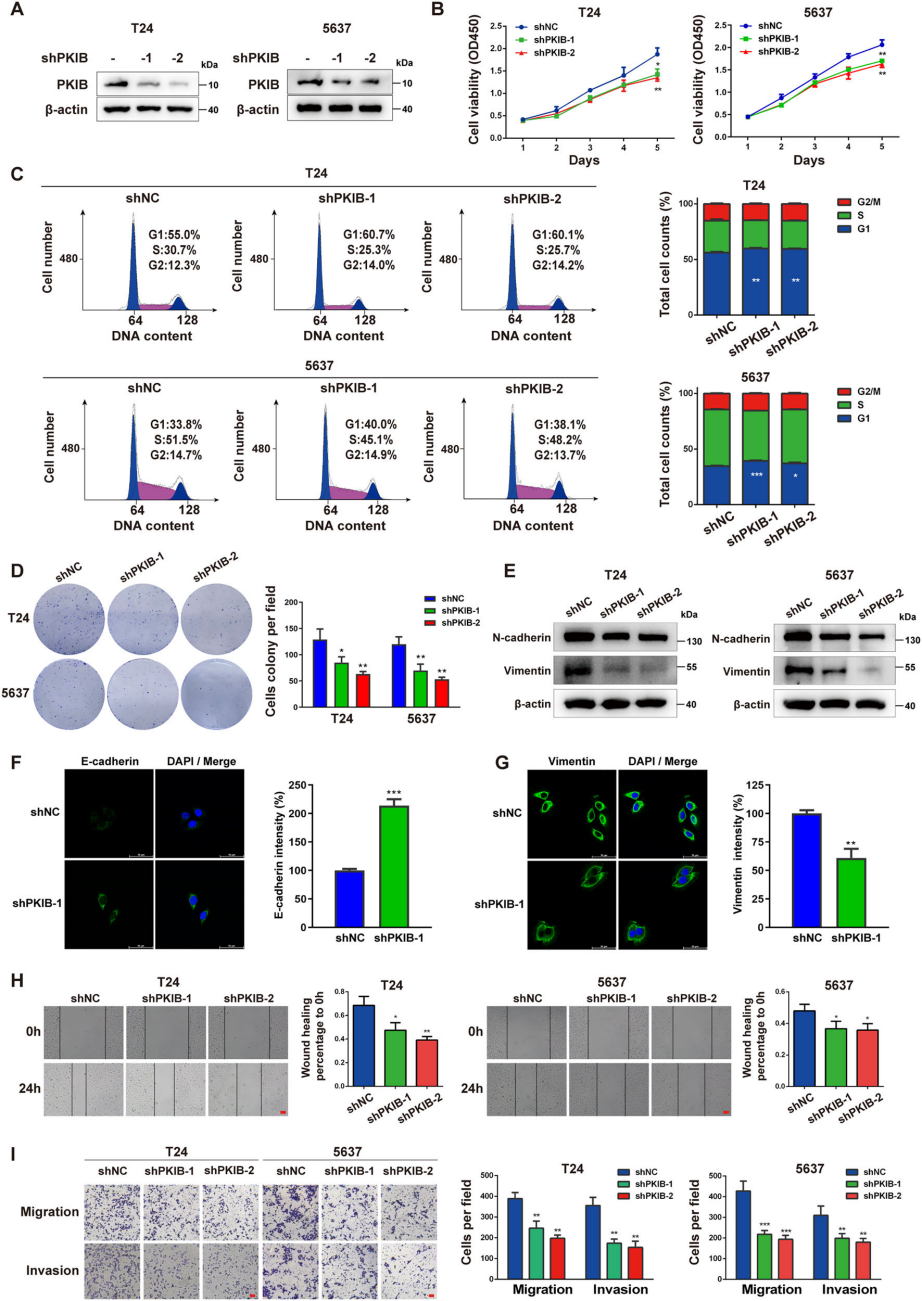
Knockdown of *PKIB* inhibits the proliferation, EMT, migration and invasion of BLCA cells. **A** Western blot analysis of PKIB protein levels in T24 and 5637 cells stably transfected with a negative control vector (shNC) or the indicated shRNA-expressing vectors of *PKIB* (shPKIB). **B** Relative growth of T24 and 5637 cells with *PKIB* knockdown or empty vector transfection, as indicated, as determined by the CCK-8 assay. The data are shown as the means ± SD (*n* = 3). T24 shPKIB-1 *P = 0.0126, shPKIB-2 **P = 0.0045; 5637 shPKIB-1 **P = 0.0057, shPKIB-2, **P = 0.0053 by unpaired Student’s *t* test. **C** The cell cycle distribution was detected by PI staining in *PKIB*-silenced and control T24 and 5637 cells. The data are shown as the means ± SD (*n* = 3). T24 shPKIB-1 **P = 0.0041, shPKIB-2 **P = 0.0056; 5637 shPKIB-1 ***P = 0.0008, shPKIB-2, *P = 0.0163 by unpaired Student’s *t* test. **D** Representative images of colony formation assay of the indicated cell lines. The data are shown as the means ± SD (*n* = 3). T24 shPKIB-1 *P = 0.0289, shPKIB-2 **P = 0.0052; 5637 shPKIB-1 **P = 0.0093, shPKIB-2 **P = 0.0013 by unpaired Student’s *t* test. **E** The expression of the EMT markers N-cadherin and Vimentin was analysed via Western blotting. β-actin served as an internal control. **F** Representative IF images of E-cadherin in control and *PKIB*-silenced T24 cells (left panel). DNA was stained with DAPI (blue). Scale bar, 50 μm. Relative intensities (right panel). **G** Representative IF images of Vimentin in control and *PKIB*-silenced T24 cells (left panel). DNA was stained with DAPI (blue). Scale bar, 50 μm. Relative intensities (right panel). **H** A wound healing migration assay was performed with *PKIB*-silenced T24 and 5637 cells as well as control cells. Wound healing was recorded and quantified at least three times. The data are shown as the means ± SD (*n* = 3). T24 shPKIB-1 *P = 0.0208, shPKIB-2 **P = 0.0032; 5637 shPKIB-1 *P = 0.0358, shPKIB-2 *P = 0.0226 according to unpaired Student’s *t* test. Scale bar, 100 μm. **I** In Transwell assays, *PKIB*-silenced T24 and 5637 cells were allowed to migrate through an 8-μm porous membrane or invade through a Matrigel-coated membrane. After 24 or 48 h, migrating and invading cells were stained and counted in at least three microscopic fields. The data are shown as the means ± SD (*n* = 3). T24 Migration shPKIB-1, ** *P* = 0.0053; shPKIB-2, **P = 0.0015. T24 Invasion shPKIB-1, **P = 0.0020; shPKIB-2, **P = 0.0021. 5637 Migration shPKIB-1, ***P = 0.0009; shPKIB-2, ***P = 0.0004. 5637 Invasion shPKIB-1, **P = 0.0097; shPKIB-2, **P = 0.0047 by unpaired Student’s *t* test. Scale bar, 100 μm.

To explore the biological functions of PKIB in BLCA cells, stable *PKIB*-knockdown T24, 5637 and RT112 cell lines were generated using lentiviruses harboring short hairpin RNA (shRNA) targeting *PKIB* (shPKIB-1, shPKIB-2) or nontargeting control shRNA (shNC) (Fig. 2A, Fig. S1A). Following this, CCK-8 and colony formation assays were utilized to quantify the proliferation potential of the knockdown cells. The results showed that knocking down *PKIB* in T24, 5637 and RT112 cells significantly attenuated their proliferation (Fig. 2B, D and Fig. S1C, S1D). To investigate the role of PKIB in the cell cycle, we conducted flow cytometry analysis, which revealed that *PKIB* knockdown caused a disruption in cell cycle progression, specifically leading to arrest at the G0/G1 to S phase transition (Fig. 2C).

Metastasis to distant organs is an important feature of BLCA. T24, 5637 and RT112 cells with *PKIB* knockdown exhibited decreased expression of mesenchymal phenotypic markers (Fig. 2E, Fig. S1A), including Vimentin and N-cadherin. Immunofluorescence (IF) also confirmed that *PKIB* knockdown in T24 cells reduced mesenchymal and increased epithelial markers (Figs. 2F, G) which indicated *PKIB* expression is positively correlated with the epithelial–mesenchymal transition (EMT) in BLCA cells. To assess the involvement of PKIB in BLCA metastasis, we conducted wound healing and transwell assays. As shown in Figs. 2H, I and Fig. S1B, the number of migrated and invaded cells was markedly decreased in *PKIB*-knockdown T24, 5637 and RT112 cells.

To further elucidate the oncogenic role of PKIB, we developed T24 and 5637 cell clones with stable expression of exogenous *PKIB* (Fig. S2A). In contrast to the *PKIB* knockdown phenotype, the *PKIB* overexpression phenotype significantly promoted cell growth (Fig. S2B) and accelerated cell cycle progression (Fig. S2C) in BLCA cells. Furthermore, overexpression of *PKIB* also led to increased levels of Vimentin and N-cadherin (Fig. S2A) and enhanced cell migration and invasion (Fig. S2D). Taken together, these data indicate that PKIB promotes BLCA cell proliferation, EMT, migration and invasion in vitro.

### PKIB inhibits PKA activity and mediates the nuclear translocation of PKA-Cα (catalytic subunit)

**Fig. 3:**
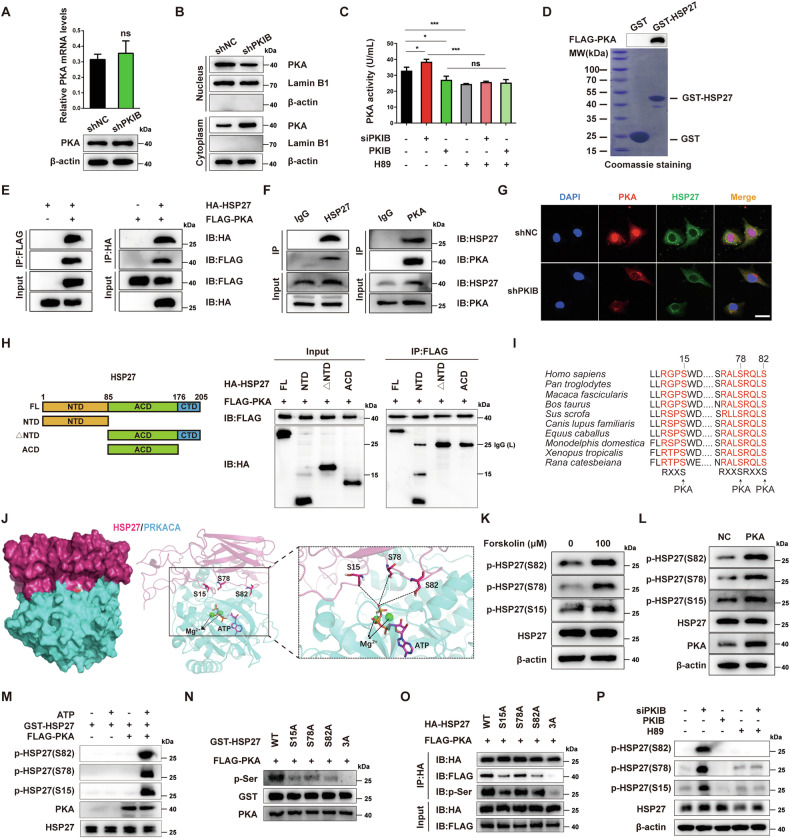
PKA directly interacts with HSP27 and phosphorylates HSP27 at serine 15, 78 and 82 in BLCA cells. **A** qRT‒PCR and Western blot analyses of PKA expression in *PKIB*-silenced T24 cells. The data are shown as the mean ± SD (*n* = 3). Statistical analysis was conducted by unpaired Student’s *t* test. **B** Western blot analyses of PKA expression in the cytoplasmic and nuclear fractions extracted from *PKIB*-silenced T24 cells. β-actin and Lamin B1 served as loading controls for cytoplasmic and nuclear lysates. **C** T24 cells were transfected with siRNA against PKIB (siPKIB) or with a *PKIB* overexpression plasmid and treated with or without H89 as indicated. The lysates were collected to assess PKA activity. The data are shown as the mean ± SD (*n* = 3). *P < 0.05, **P < 0.01, ***P < 0.001, ns = not significant according to one-way ANOVA with Tukey’s multiple comparison tests. **D** Bacterially purified *E. coli* BL21 GST-HSP27 or GST protein was incubated with active FLAG-PKA for a GST pull-down assay. **E** The exogenous interaction between PKA and HSP27 was confirmed by Co-IP using anti-FLAG or anti-HA antibodies in HEK293T cells cotransfected with FLAG-PKA and HA-HSP27. **F** The endogenous interaction between PKA and HSP27 was determined by Co-IP using anti-PKA or anti-HSP27 antibodies in T24 cells. **G** Immunofluorescence analysis was performed to evaluate the colocalization of endogenous PKA (red) and HSP27 (green) in T24 cells with *PKIB* knockdown or empty vector transfection. The nuclei were stained with DAPI (blue). Scale bar, 25 μm. **H** Full-length and three truncated CDSs of HSP27 (denoted FL, NTD, △NTD, and CTD) were used for the construction of HA-tagged HSP27 expression vectors (left panel). The abovementioned HA-tagged HSP27 vectors and FLAG-tagged PKA vector were cotransfected into HEK293T cells as indicated for 48 h, after which the cells were subjected to Co-IP assays to detect the domains of HSP27 that interact with PKA (right panel). **I** The N-terminal amino acid sequences of HSP27 proteins from different species were aligned. The conserved serine 15 residue (S15), serine 78 residue (S78) and serine 82 residue (S82) are predicted to be potential PKA phosphorylation sites in above species. **J** The modeled structures of HSP27 (warmpink) and PRKACA (cyan) are shown as surfaces (left panel) and cartoons (right panel), respectively. The ATP and three potential phosphorylation sites (S15, S78 and S82) are represented as colored sticks, and the Mg^2+^ sites are shown as green spheres. **K** T24 cells were treated with forskolin as indicated for 1 h, then western blot analysis was used to determine the level of phosphorylated HSP27. **L** Western blot analysis of the phosphorylation level of HSP27 in T24 cells overexpressing PKA. **M** HEK293T cells were transfected with the FLAG-tagged PKA vector, followed by IP with anti-FLAG magnetic beads. Purified proteins were incubated with *E. coli* BL21 bacterially purified GST-HSP27 for the kinase assay, and immunoblot analysis was performed to measure the level of phosphorylated HSP27. **N** In vitro GST-HSP27 (WT or mutant) proteins were incubated with active FLAG-PKA for an in vitro kinase assay. The total amount of phosphorylated HSP27 was quantified via densitometry. **O** HA-tagged HSP27 (WT or mutant) vectors and the FLAG-tagged PKA vector were cotransfected into HEK293T cells as indicated for 48 h, after which the cells were subjected to IP with anti-HA magnetic beads to detect the binding of HSP27 mutants to PKA. **P** T24 cells were transfected with siPKIB or with the PKIB overexpression vector and treated with or without H89 as indicated. Immunoblot analysis was performed to detect the protein level of phosphorylated HSP27.

Although PKIB is a member of the PKI family, its inhibitory effect on PKA has not been fully characterized [29]. To assess the effect of PKIB on PKA, we analyzed mRNA and protein levels of the catalytic subunit PKA-Cα in *PKIB*-silenced T24 cells. qRT‒PCR and Western blot analysis revealed that *PKIB* knockdown did not affect the mRNA or protein levels of PKA-Cα (Fig. 3A). However, the subcellular localization of the PKA-Cα protein was altered upon *PKIB* knockdown, leading to an increase in the cytoplasmic PKA-Cα level and a decrease in the nuclear PKA-Cα level (Fig. 3B).

To evaluate the effect of PKIB on PKA kinase activity, we transfected T24 cells with siPKIB or the *PKIB* overexpression plasmid and treated them with the PKA inhibitor H89. The ELISA assay indicated that *PKIB* overexpression led to a substantial decrease in PKA activity, comparable to the effect of H89. In contrast, *PKIB* knockdown caused a significant increase in PKA activity. This elevated PKA activity associated with *PKIB* knockdown was entirely reversed by H89 (Fig. 3C). These findings suggest that PKIB inhibits the kinase activity of PKA in BLCA cells.

### PKIB mediates the phosphorylation of serine 15, 78 and 82 in HSP27 via PKA

Given that the important roles of PKA in different cancer types may be mediated by different substrate proteins, we conducted a phosphoproteomic analysis using *PKIB*-knockdown T24 cells to identify potential PKA substrates regulated by PKIB in BLCA. In total, we identified 37 significantly upregulated phosphorylated proteins, including the known PKA substrate GSK3β (Fig. S3B, Table S5), and 30 proteins with significantly downregulated phosphorylation. Among the top 10 proteins sequentially ranked according to the fold change value of upregulated phosphorylation, 6 proteins (FRYL, SYNRG, SRRM2, HSP27, RAPH1 and ERBIN) contained the canonical PKA phosphorylation motif RXXS, but only the phosphorylation of HSP27 has been widely reported in tumor biology. Therefore, among these proteins, heat shock protein 27 (HSP27/HSPB1) has attracted our special attention, although previous studies generally suggested that HSP27 may not be a genuine substrate for PKA [45]. HSP27 typically possesses three potential phosphorylation sites: Ser15 (S15), Ser78 (S78) and Ser82 (S82) [46]. We also conducted Western blot analysis on T24 and other BLCA cells (RT112 and 5637) with *PKIB* knockdown (Fig. 3P, S3C, S4D), which likewise revealed a significant elevation in HSP27 phosphorylation (S15, S78, and S82). These outcomes imply that HSP27 might serve as a potential PKA-interacting protein in BLCA.

The interaction between PKA and HSP27 was first detected by a GST pull-down assay in which the GST-HSP27 fusion protein or GST alone was incubated with purified FLAG-tagged PKA. The pull-down results showed that under our experimental conditions, FLAG-tagged PKA bound to GST-HSP27 but not to GST alone (Fig. 3D). We further performed Co-IP assays using exogenous proteins in HEK293T cells cotransfected with HA-HSP27 and FLAG-PKA plasmids (Fig. 3E) or endogenous proteins in T24 cells (Fig. 3F), confirming that PKA interacts with HSP27. Moreover, immunofluorescence staining revealed that PKA was localized in both the cytoplasm and the nucleus, whereas HSP27 was almost exclusively localized in the cytoplasm. These two proteins predominantly colocalized in the cytoplasm. When PKIB was knocked down, additional PKA translocated from the nucleus to the cytoplasm where it colocalized with HSP27 (Fig. 3G). Thus, these findings suggest that PKA directly interacts with HSP27 in BLCA cells.

HSP27 is comprised of a central α-crystallin domain (ACD) that is bordered by flexible N-terminal (NTD) and C-terminal (CTD) regions. To further explore the structural determinants of the interaction between HSP27 and PKA-Cα, three different truncations of HSP27 were generated (Fig. 3H, left). The different truncations were then cotransfected with FLAG-tagged PKA-Cα into HEK293T cells. Co-IP assays showed that PKA coprecipitated with the NTD-containing domain but not with the ACD or CTD of HSP27 (Fig. 3H, right), indicating that the NTD domain is crucial for the interaction between HSP27 and PKA. Interestingly, three phosphorylation sites in HSP27 (S15, S78, and S82) are located in the NTD domain, and sequence analysis also revealed that all of these sites correspond to the canonical PKA phosphorylation motif RXXS (Fig. 3I).

We also constructed protein homology models for human PRKACA (PKA-Cα) and HSP27 and performed molecular docking and molecular dynamics (MD) simulations of the complex to study its conformational dynamics (Fig. S4). The single-protein models of PRKACA and HSP27 were evaluated using PROCHECK (Fig. S4A). As shown in Fig. S4D, >97% of the regions were favorable for HSP27, and this value was 100% for the PRKACA model. The top three best scored poses of the complexes (complex 1, score 1833; complex 2, score 1525; and complex 3, score 1511) were further evaluated by CCharPPI (https://life.bsc.es/pid/ccharppi) [47]. Seven comprehensive scoring functions, namely, ZRANK, ROSETTADOCK, PYDOCK_TOT, FIREDOCK, AP_PISA, CP_PIE and SIPPER, were used to evaluate the rationality of the conformations of the three complexes (Fig. S4E). According to the evaluation results, complex 1 was the best composite model, and this model was used for further analysis (Fig. S4B). To determine the structural stability of the PRKACA/HSP27 complex, we performed an all-atom molecular dynamics simulation of the constructed complex. The root mean square deviation (RMSD) of the Cα atoms was used to assess the deviation of the structures from the initial coordinates (Fig. S4F). The system demonstrated convergence between 200 and 500 ns, with RMSD values stabilizing in the final 300 ns of the simulations. The conformational changes in the complex mainly occur in the loop region of HSP27. Interestingly, the conformation of the binding interface of the two proteins was relatively stable, and the relative positions and distances of S15, S78 and S82 to the ATP molecule remained constant ( ~ 6.0 Å, ~7.5 Å and ~8.5 Å, respectively) and stable during the 500 ns dynamics simulation (Fig. 3J). The results of molecular docking analysis and molecular dynamics simulation showed that HSP27 has a strong binding affinity for PRKACA, and the serine residues S15, S78 and S82 of HSP27 are the most likely phosphorylation sites.

We subsequently tested whether PKA phosphorylates these three sites on HSP27. Since elevated cAMP levels can lead to PKA activation, we treated T24 cells with forskolin, a pharmacological activator of adenylate cyclase. The results showed that HSP27 phosphorylation at S15, S78 and S82 was significantly increased (Fig. 3K). Similarly, when PKA-Cα was overexpressed in T24 cells, the phosphorylation of the three sites in HSP27 was also increased (Fig. 3L). To exclude the influence of other kinases in vivo, a kinase assay was performed in vitro using purified GST-tagged HSP27 and the PKA-Cα active protein (Fig. 3M). Western blotting also revealed that phosphorylation at these three sites in HSP27 was increased. These results indicate that the S15, S78 and S82 sites of the HSP27 protein can be phosphorylated by PKA in BLCA cells. To investigate whether there are other serine residues on the HSP27 protein that can be phosphorylated by PKA, we constructed four nonphosphorylated HSP27 mutants, namely, the Ala substitution Ser15 (S15A), Ser78 (S78A) and Ser82 (S82A) mutants, as well as the S15A/S78A/S82A triple mutant (3 A). Serine phosphorylation of HSP27 was then detected in vitro (Fig. 3N). The results showed that PKA-mediated serine phosphorylation of HSP27 was significantly reduced in all three single mutants, with the S15A mutant having a lower phosphorylation level than S78A or S82A. In addition, serine phosphorylation was almost undetectable in the HSP27-3A mutant. Furthermore, we detected the binding of four HSP27 mutants to PKA by Co-IP assays (Fig. 3O). Compared with that of wild-type HSP27, the binding of the S15A, S78A and S82A mutants to PKA was significantly weakened, whereas the binding of the HSP27-3A mutant to PKA was limited. These results strongly suggest that PKA binds and primarily phosphorylates the S15, S78, and S82 serine residues of HSP27. To further explore the association between PKIB and PKA-mediated phosphorylation of HSP27, we examined the phosphorylation of three serine residues in HSP27 after *PKIB* overexpression, *PKIB* silencing and H89 treatment in T24 cells (Fig. 3P). *PKIB* silencing increased the phosphorylation of HSP27 at S15, S78, and S82. As with H89 treatment, overexpression of *PKIB* significantly inhibited the phosphorylation of these three sites. In addition, H89 blocked the effect of *PKIB* silencing on the phosphorylation of these sites in HSP27. These findings suggested that PKIB affects the phosphorylation of HSP27 by inhibiting PKA kinase activity in BLCA cells.

### HSP27 overphosphorylation inhibits BLCA cells viability and invasion

**Fig. 4:**
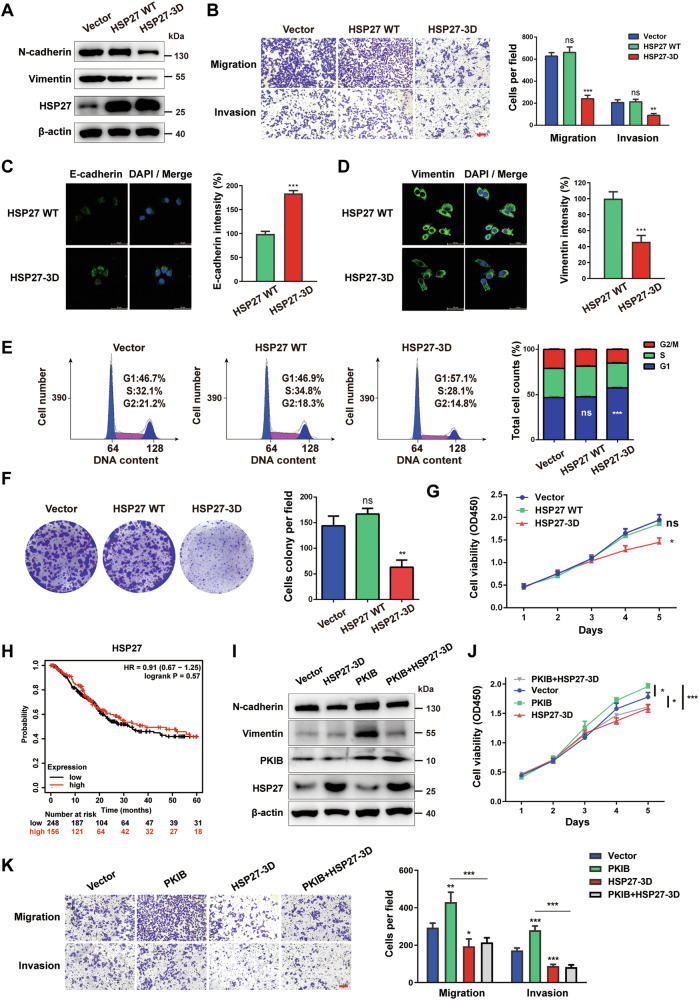
HSP27 overphosphorylation inhibits the proliferation, EMT, migration and invasion of BLCA cells. **A** Western blot analysis of HSP27 and EMT markers (N-cadherin and Vimentin) protein levels in T24 cells stably transfected with a negative control vector (NC), wild-type HSP27 (HSP27 WT) or an HSP27-overexpressing mutant (HSP27-3D). **B** The migratory and invasive capabilities of the above-treated T24 cells were evaluated via Transwell assays. The data are shown as the mean ± SD (*n* = 3). ^**^P = 0.0026, ^***^P = 0.0001, ns = not significant according to unpaired Student’s *t* test. Scale bar, 100 μm. **C** Representative IF images of E-cadherin in HSP27 WT and HSP27-3D T24 cells (left panel). DNA was stained with DAPI (blue). Scale bar, 50 μm. Relative intensities (right panel). **D** Representative IF images of Vimentin in HSP27 WT and HSP27-3D T24 cells (left panel). DNA was stained with DAPI (blue). Scale bar, 50 μm. Relative intensities (right panel). **E** The cell cycle distribution was detected in the above-described treated T24 cells. The data are shown as the mean ± SD (*n* = 3). ^***^P < 0.0001, ns = not significant according to unpaired Student’s *t* test. **F** Colony formation assay of HSP27-overexpression and HSP27 over-phosphorylation of T24 cells. The data are shown as the mean ± SD (*n* = 3). ^**^P = 0.0040, ns = not significant according to unpaired Student’s *t* test. **G** CCK8 assay for HSP27-overexpressing and HSP27-overphosphorylation T24 cells. The data are shown as the mean ± SD (*n* = 3). ^**^P = 0.0048, ns = not significant according to unpaired Student’s *t* test. **H** Kaplan-Meier survival curves (http://kmplot.com/analysis/) of patients with bladder carcinoma (*n* = 408) with high or low HSP27 expression. The log-rank test was used to analyse the difference between two groups. **I** T24 cells were cotransfected with PKIB and HSP27-3D and then subjected to Western blot analysis to detect the expression of PKIB, HSP27 and EMT markers (N-cadherin and Vimentin). **J** CCK8 assay of the above-treated T24 cells. The data are shown as the mean ± SD (*n* = 3). PKIB ^*^P = 0.0216; PKIB + HSP27-3D ^*^P = 0.0331; HSP27-3D ^*^P = 0.0159; ^***^P = 0.0004, ns = not significant according to one-way ANOVA with Tukey’s multiple comparison tests. **K** The migratory and invasive capabilities of T24 cells cotransfected with PKIB and HSP27-3D were evaluated via Transwell assays. The data are shown as the mean ± SD (*n* = 3). Migration PKIB ^**^P = 0.0092; HSP27-3D ^*^P = 0.0449; ^***^P = 0.0005. Invasion PKIB ^***^P = 0.0001; HSP27-3D ^***^P = 0.0007. ^***^P < 0.0001 according to one-way ANOVA with Tukey’s multiple comparison tests. Scale bar, 100 μm. The numbers of migrated and invasive T24 cells are presented.

We have observed that *PKIB* downregulation led to reduced proliferation and invasion of BLCA cells, accompanied by increased phosphorylation of HSP27. To investigate whether elevated phosphorylation of HSP27 can induce cell phenotypes similar to those associated with *PKIB* downregulation, we overexpressed a mimicking phosphorylated triple mutant of HSP27 in T24 cells in which serine residues at positions 15, 78, and 82 were mutated to aspartic acid (HSP27-3D). As shown in Fig. 4A, the protein levels of the mesenchymal markers Vimentin and N-cadherin were lower in HSP27-3D cells than in HSP27-overexpressing and control cells, indicating that elevated phosphorylation of HSP27 is negatively associated with epithelial–mesenchymal transition in BLCA cells. Immunofluorescence (IF) also confirmed that HSP27-3D increased epithelial markers and reduced mesenchymal (Figs. 4C, D). Transwell assays were further conducted to evaluate cell migration and invasion (Fig. 4B). The results indicated a significant reduction in both migration and invasion capabilities in HSP27-3D cells, whereas HSP27-overexpressing cells showed no notable alterations in these processes. Additionally, the impact of HSP27 phosphorylation on T24 cell proliferation was assessed. The HSP27-3D variant caused cell cycle arrest at the G0/G1 to S phase transition (Fig. 4E) and significantly impaired colony formation and proliferation capacity, as evidenced by colony formation assays (Fig. 4F) and CCK-8 assays (Fig. 4G). Remarkably, the overexpression of HSP27-WT had no effect on these phenotypes. To validate these findings in a clinical context, bladder cancer patient samples in the TCGA database were divided into low and high *HSP27* expression groups, and overall survival was analysed using the Kaplan‒Meier Plotter (http://kmplot.com/analysis/). The analysis demonstrated that elevated *HSP27* expression did not correlate with adverse prognosis in BLCA patients (Fig. 4H), which corroborates the findings from the cell phenotyping experiments. Taken together, these results indicate that phosphorylation rather than overexpression of HSP27 inhibits BLCA cell viability and invasion, which is similar to the phenotypic changes observed in PKIB-knockdown cells.

To further confirm that PKIB regulates these biological processes by altering the phosphorylation of HSP27, we performed rescue experiments using T24 cells overexpressing *PKIB* and *HSP27-3D*. Introduction of *HSP27-3D* restored the increase in the expression of the N-cadherin and Vimentin proteins induced by *PKIB* overexpression (Fig. 4I). We then assessed the effect of overexpressing *PKIB* and *HSP27-3D* on cell phenotypes. According to the CCk8 assay, HSP27-3D effectively counteracted the effects of *PKIB* overexpression and blocked the proliferation of T24 cells (Fig. 4J). In addition, HSP27-3D successfully restored the increase in cell migration and invasion induced by *PKIB* overexpression (Fig. 4K). Collectively, the above results demonstrate that PKIB promotes cell proliferation and invasion in BLCA by mediating the inhibition of HSP27 phosphorylation.

### S15-mediated phosphorylation of HSP27 is more important for inhibiting BLCA cell proliferation and metastasis

**Fig. 5:**
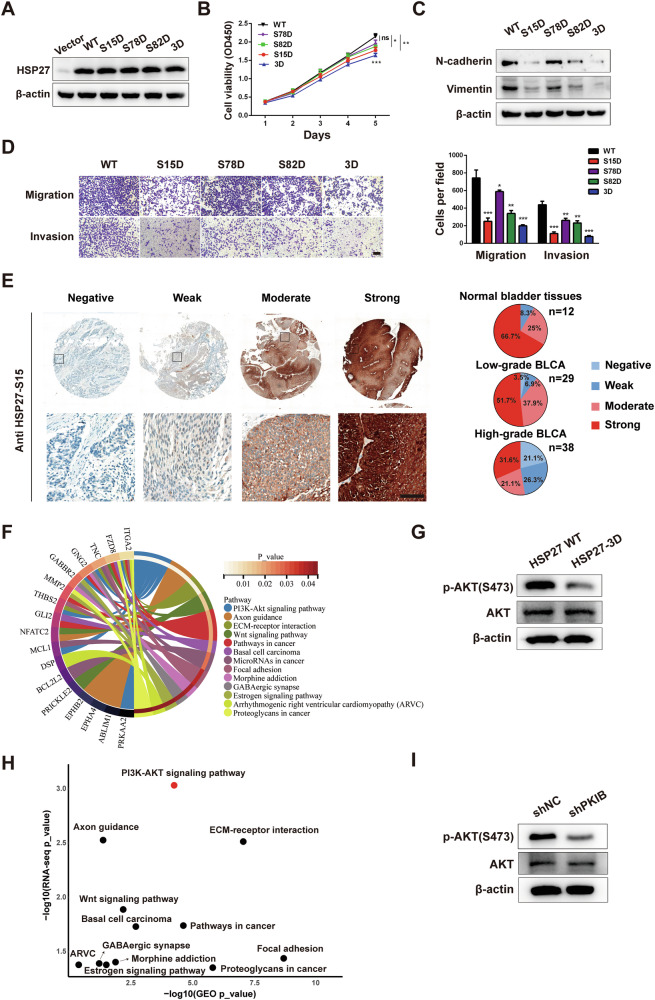
Overphosphorylation of HSP27 at residue 15 is more potent in BLCA cells inhibition. **A** T24 cells were transfected with wild-type HSP27 or HSP27-S15A/S78A/S82A/3D mutants, then Western blot analysis of T24 cells with ectopic HSP27 expression was performed. **B** A CCK8 assay was used to assess the proliferation ability of the above-described treated T24 cells. The data are shown as the mean ± SD (*n* = 3). ^*^P = 0.0467, ^**^P = 0.0037, ^***^P = 0.0006, ns = not significant according to unpaired Student’s *t* test. **C** The expression of the EMT markers (N-cadherin and Vimentin) were analysed via Western blotting. **D** A transwell assay was performed to assess the migration and invasion of the above-described treated T24 cells. The data are shown as the mean ± SD (*n* = 3). Migration ^*^P = 0.0414, ^**^P = 0.0018, S15D ^***^P = 0.0009, 3D ^***^P = 0.0005; Invasion S15D ^***^P = 0.0002, S78D ^**^P = 0.0026, S82D ^**^P = 0.0016, 3D ^***^P <0.0001 according to unpaired Student’s *t* test. Scale bar, 100 μm. The numbers of migrated and invasive T24 cells are presented. **E** Representative immunohistochemistry (IHC) images of HSP27-S15 staining in normal and different grade BLCA tissues (left panel). Differential percentage of low HSP27-S15 expression in normal and various BLCA tissues (right panel). **F** Total RNA isolated from T24 cells overexpressing *HSP27* or overphosphorylating HSP27 was subjected to RNA-seq analysis. Kyoto Encyclopedia of Genes and Genomes (KEGG) analysis revealed that HSP27 overphosphorylation in T24 cells downregulated the PI3K-AKT signaling pathway. The detailed relationships between the DEGs and major pathways annotated by KEGG are shown in the Circos graph. Differentially expressed genes with log2 FC > 2 were chosen for analysis and are shown on the left side of the graph. Representative signaling pathways are shown on the right. **G** Western blot analysis of total AKT and phosphorylated AKT S473 protein levels in T24 cells stably transfected with wild-type HSP27 or the 3D mutant. HSP27 overphosphorylation in T24 cells decreased the phosphorylation of AKT at serine 473. **H** The GEO database was used to screen for upregulated pathways in BLCA tissues overexpressing *PKIB*. Twelve signaling pathways were found to be upregulated by *PKIB* overexpression and downregulated by HSP27 phosphorylation. **I** Western blot analysis of total AKT and phosphorylated AKT S473 protein levels in *PKIB*-silenced T24 cells. *PKIB* knockdown in T24 cells reduces the phosphorylation of AKT at serine 473.

As shown above, PKIB-mediated phosphorylation of HSP27 at these three serine residues results in a tumor suppressor effect. To further investigate which specific site phosphorylation has the greatest impact on inhibiting the oncogenic potential of BLCA, we substituted each serine with aspartic acid and constructed three single mutants of HSP27 that mimic phosphorylation in T24 cells, named S15D, S78D and S82D (Fig. 5A). CCK8 assays indicated that HSP27-S15D most significantly suppressed the proliferative capability of BLCA cells, while S78D did not cause significant changes in the cell growth rate (Fig. 5B). Moreover, compared with S78D and S82D, HSP27-S15D led to a greater decrease in the expression of the mesenchymal markers N-cadherin and Vimentin (Fig. 5C) and inhibited cell migratory and invasive capabilities more effectively in T24 cells (Fig. 5D). These results imply that S15 phosphorylation of HSP27 is more important than S78 or S82 phosphorylation for inhibiting BLCA cell proliferation and metastasis. Additionally, immunohistochemical (IHC) staining analysis was conducted to determine the clinical significance of HSP27-S15 phosphorylation in different BLCA tissues (Table S6). HSP27-S15 was strongly and moderately phosphorylated in 91.7% of normal bladder tissues (*n* = 12), compared with 89.6% in low-grade (*n* = 29) and 52.7% in high-grade BLCA tissues (*n* = 38) (Fig. 5E), suggesting that the phosphorylation level of HSP27-S15 decreased significantly (Chi-square test, *P* < 0.001) with the progression of BLCA.

### HSP27 phosphorylation inhibits the activation of the AKT/MMP2 signaling pathway

To investigate the downstream molecular pathways involved in HSP27 phosphorylation in BLCA progression, RNA-seq analysis was utilized to compare T24 cells with HSP27-phosphorylated (HSP27-3D) and HSP27-overexpressing (HSP27-WT) cells. This analysis revealed 98 downregulated and 112 upregulated genes at the mRNA level in HSP27-3D cells (Table S7). KEGG enrichment analysis identified the PI3K-AKT signaling pathway as the most prominently enriched among the downregulated genes in HSP27-3D cells (Fig. 5F). Notably, previous studies have reported the direct interaction between HSP27 and AKT [48]. Considering the pivotal role of AKT in the PI3K-AKT signaling pathway and the necessity of AKT-S473 phosphorylation for its activation, we examined the phosphorylation status of AKT-S473 in T24 cells. Phosphorylation of AKT in HSP27-3D cells was significantly lower than that in HSP27-WT cells (Fig. 5G). Because *PKIB* knockdown leads to enhanced phosphorylation of HSP27, we further used the GEO database (GSE13507) to screen for upregulated pathways in BLCA tissues with high *PKIB* expression. Interestingly, the PI3K-AKT signaling pathway was also identified in the enrichment list (Fig. 5H), consistent with the results of the RNA-seq analysis above. We also observed reduced phosphorylation of AKT-S473 in T24 cells after *PKIB* knockdown (Fig. 5I). These collective results strongly suggest that PKIB-mediated phosphorylation of HSP27 inhibits the activation of the PI3K-AKT signaling pathway in BLCA.

In addition, RNA-seq analysis also showed that the *MMP2* gene was significantly downregulated (log2FC = –2.6) in HSP27-3D T24 cells (Table S7). MMP2, known to be regulated by the PI3K-AKT signaling pathway, is a key factor in tumor metastasis [49, 50]. We first assessed the protein expression of MMP2 in PKIB-knockdown T24 cells and found that the MMP2 protein level was significantly reduced (Fig. S5A). Subsequently, we overexpressed *MMP2* in *PKIB*-knockdown T24 cells and performed CCK-8 and transwell assays. As expected, overexpression of *MMP2* successfully rescued the proliferative and migratory abilities of T24 cells inhibited by *PKIB* knockdown (Fig. S5B, S5C), suggesting that MMP2 may be an important downstream protein for PKIB to influences the BLCA phenotypes.

### MYCN regulates the transcription of *PKIB* in BLCA

**Fig. 6:**
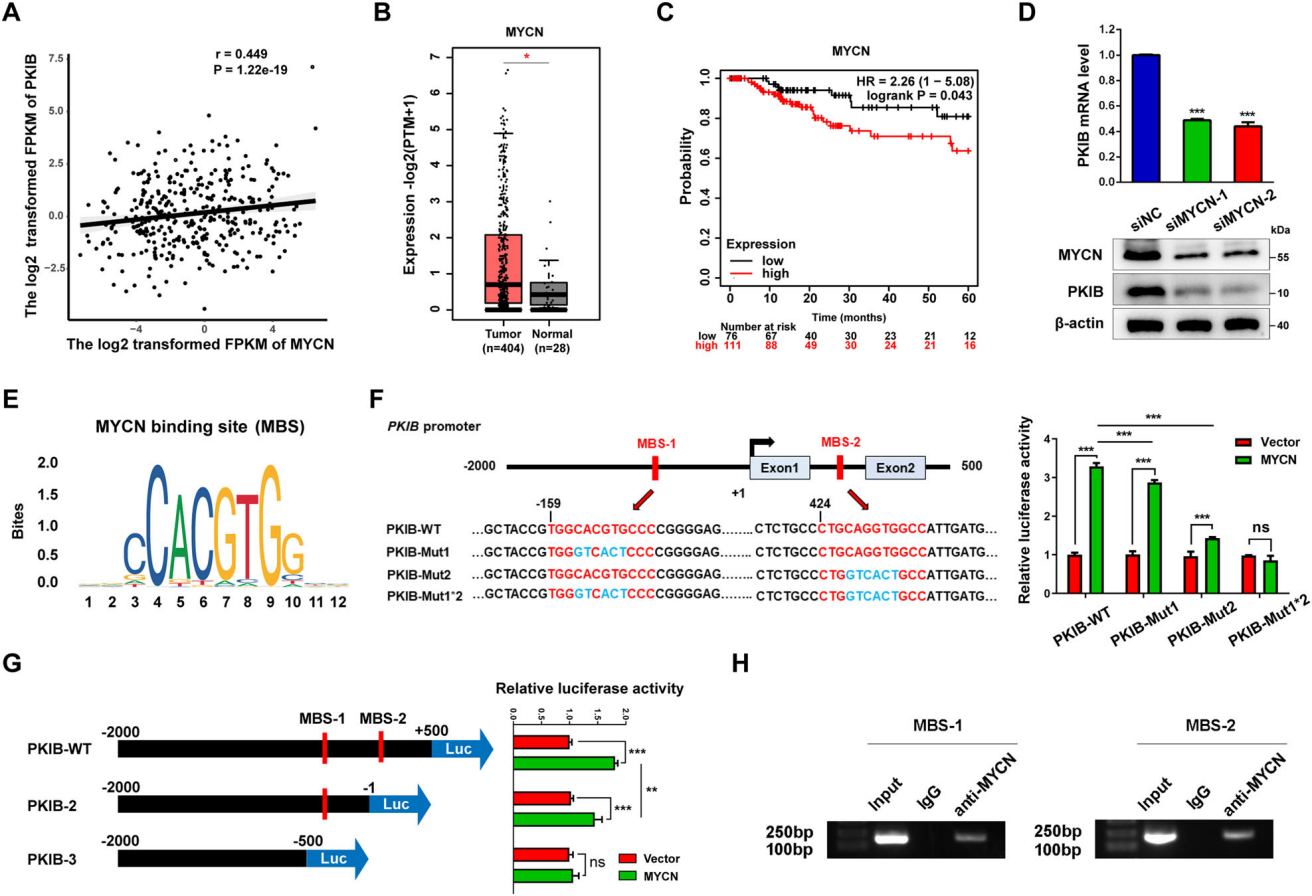
MYCN directly interacts with the *PKIB* promoter in BLCA cells. **A** Correlation of *MYCN* and *PKIB* mRNA expression in BLCA tissues. The X- and Y-axes represent the log2-transformed FPKMs of *MYCN* and *PKIB* mRNA in BLCA tissues, respectively. **B** mRNA expression level of *MYCN* in BLCA and normal tissues in the TCGA database. (http://gepia2.cancer-pku.cn/#index). ^*^P < 0.05. **C** Kaplan-Meier survival curves (http://kmplot.com/analysis/) of bladder cancer patients (*n* = 408) with high or low expression levels of *MYCN*. The log-rank test was used to analyse the difference between two groups. **D** qRT–PCR and Western blot analyses of *PKIB* mRNA and protein levels in *MYCN*-silenced T24 cells. qRT–PCR data are shown as the mean ± SD (*n* = 3). ^***^P < 0.0001 according to unpaired Student’s *t* test. **E** The conserved MYCN binding site (MBS) is shown (https://jaspar.genereg.net/matrix/MA0104.4/). **F** Schematic showing two predicted binding sites for MYCN in the *PKIB* promoter (upper left panel). Boxed areas indicate several *PKIB* promoter segments containing wild-type (PKIB-WT) or mutant MBS-1/2 (PKIB-Mut1, PKIB-Mut2, or PKIB-Mut1*2; lower left panel), which were subcloned and inserted into the pGL3-Luc reporter vector. The numbers indicate the location of the nucleotides of the *PKIB* promoter. The constructs were transiently transfected into 293 T cells overexpressing *MYCN*, after which luciferase activities were determined (right panel). The data are shown as the mean ± SD (*n* = 3). PKIB-WT vs PKIB-Mut1 ^***^P = 0.0003, others ^***^P < 0.0001, ns = not significant according to one-way ANOVA with Tukey’s multiple comparison tests. **G** Different fragments of the *PKIB* promoter (PKIB-WT, PKIB-2 and PKIB-3) were subcloned and inserted into the pGL3-Luc reporter vector (left panel), which was transiently transfected into 293 T cells overexpressing *MYCN*, after which luciferase activity was determined (right panel). The data are shown as the mean ± SD (*n* = 3). ^**^P = 0.0018, PKIB-WT ^***^P < 0.0001, PKIB-2 ^***^P = 0.0018, ns = not significant according to one-way ANOVA with Tukey’s multiple comparison tests. **H** The ChIP assay was performed on T24 cells using an anti-MYCN antibody. An anti-IgG antibody was used as a negative control. The immunoprecipitated DNA fragment was subjected to PCR and analysis to determine the enrichment of MYCN.

To further investigate the regulatory effect of *PKIB* gene overexpression on BLCA, we utilized the TCGA database to analyse the transcription factors that were positively correlated with *PKIB* mRNA expression. A total of 31 potential candidates (r > 0.3) were identified (Table S8). To determine which transcription factor could bind to the *PKIB* promoter (positions –3000 to –1), we employed the multiple EM for motif elicitation (MEME) analysis tool. This analysis revealed seven putative transcription factors (Table S8). The expression levels of these transcription factors in BLCA were subsequently assessed using the TCGA database through the Gene Expression Profiling Interactive Analysis 2 platform (http://gepia2.cancer-pku.cn). Notably, only *MYCN* exhibited both a positive correlation with *PKIB* expression and significantly higher expression in BLCA tissues compared to normal tissues (Fig. 6A, B). In addition, Kaplan‒Meier survival analysis using the Kaplan‒Meier Plotter demonstrated a significant association between high *MYCN* expression and poor prognosis in BLCA patients (Fig. 6C).

We conducted experiments to verify the role of MYCN in regulating *PKIB* transcription in BLCA cells. First, the expression of the *PKIB* gene was measured in T24 cells with *MYCN* knockdown (siRNA), and the results showed that both the mRNA and protein expression of *PKIB* were significantly reduced (Fig. 6D), suggesting that *PKIB* expression may be regulated by the MYCN. Then, we used the JASPAR database (http://jaspar.genereg.net/) to search for potential MYCN binding sites (MBS) in the *PKIB* gene sequence (Fig. 6E). Sequence analysis revealed two putative binding sites located at positions –159 to –148 (MBS-1) in the *PKIB* promoter region and 424–435 (MBS-2) in the first intron (Fig. 6F).

To test whether MYCN regulates *PKIB* gene transcription by binding to these two prediction sites, we constructed a series of dual-luciferase reporter vectors containing *PKIB* promoter fragments, including the wild type and three mutants (Mut1, Mut2 and Mut1/Mut2), as shown in Fig. 6F (left panel). The construct was then cotransfected into 293 T cells with the *MYCN* overexpression plasmid. Luciferase activity was increased in the wild-type *PKIB* promoter but was significantly reduced in both the Mut1 and Mut2 mutant strains. Notably, the luciferase activity of the Mut1/Mut2 double mutant was almost completely eliminated (Fig. 6F, right panel). We also designed luciferase reporter vectors containing *PKIB* promoter fragments of different lengths (PKIB-WT, PKIB-2 and PKIB-3), as shown in Fig. 6G. Compared with that of the PKIB-WT vector containing all the predicted sites, the luciferase activity of PKIB-2 containing only MBS-1 was significantly lower, whereas the luciferase activity of PKIB-3 (positions -2000 to -500) without MBS-1 and MBS-2 was completely eliminated in 293 T cells. This finding is consistent with the results for the mutants, suggesting that the specific sites in the *PKIB* promoter region are critical for its transcriptional regulation by MYCN. To further validate the results of these luciferase assays and to confirm the direct interaction between MYCN and the *PKIB* promoter binding site, we also performed a chromatin immunoprecipitation (ChIP) assay in T24 cells. In this analysis, we observed significant enrichment of MBS-1 or MBS-2 in the DNA fragments immunoprecipitated by the MYCN antibody (Fig. 6H), indicating direct binding of MYCN to these sites. Taken together, these results suggest that MYCN binds directly to the promoter of *PKIB* and regulates its transcription in BLCA.

### PKIB silencing or HSP27 phosphorylation can inhibit the metastasis and proliferation of BLCA cells in vivo

**Fig. 7:**
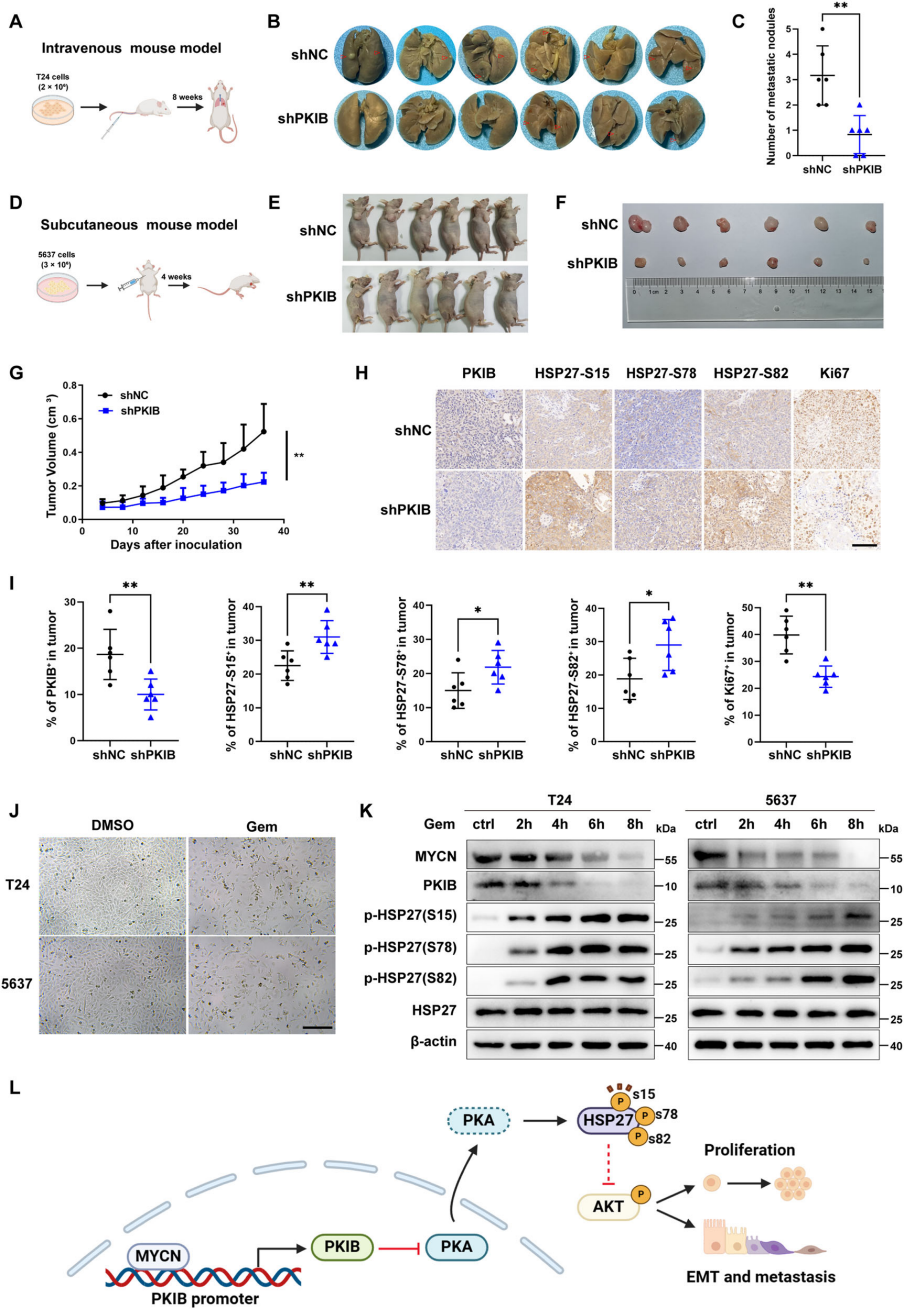
Knockdown of *PKIB* suppresses BLCA cell metastasis and proliferation in vivo. **A** Schematic flowchart of the in vivo metastasis model of BLCA cells. *PKIB*-silenced and control vector–transfected T24 cells (2 × 10^6^ cells/mouse) were intravenously injected into BALB/c nude mice (6 mice per group). **B** Photographs of metastatic nodules established in mice after injection of indicated T24 cells for 8 weeks. Red arrowheads indicate metastatic nodules formed in lungs. **C** Plots showing the difference in lung metastatic nodules between the *PKIB*-silenced group and vector control group (6 mice per group). ^**^P = 0.0021 by unpaired Student’s *t* test. **D** Schematic flowchart of the in vivo proliferation model of BLCA cells. *PKIB*-silenced and control vector–transfected 5637 cells (3 × 10^6^ cells/mouse) were subcutaneously injected into BALB/c nude mice (6 mice per group). **E**–**G** Xenograft tumorigenesis of *PKIB*-silenced T24 cells. Six nude mice were used for each group, and tumor growth curves were generated every 4 days. Images of tumors (**E**) and image of tumor volumes (**F**) in nude mice. **G** Growth curves of tumors formed by the indicated cells. ^**^P = 0.0018 by unpaired Student’s *t* test. Representative immunohistochemistry (IHC) images (**H**) and quantitation (**I**) of mouse tumors showing PKIB, phosphorylation of HSP27 at S15, S78, S82 and Ki67. Scale bar, 100 μm. **J** T24 and 5637 cells were treated with 10 nM gemcitabine or DMSO for 24 h. Scale bar, 100 μm. **K** T24 and 5637 cells were treated with 10 nM gemcitabine for 0 h, 2 h, 4 h, 6 h or 8 h, then Western blot analysis were performed. **L** A schematic model showing that PKIB acts as a PKA inhibitor and inhibits PKA-mediated phosphorylation of HSP27 to promote tumor proliferation and metastasis.

Furthermore, we used in vivo tumor models to assess the potential of targeting *PKIB* to suppress BLCA metastasis and proliferation. First, T24 cells (2 × 10^6^) with stable *PKIB* knockdown or a negative control vector were intravenously injected into BALB/c nude mice. After 8 weeks of inoculation, the mice were humanely killed and their lungs were surgically excised and examined for metastatic lung lesions (Fig. 7A). The lungs were stained with Bouin’s solution and hematoxylin and eosin (H&E), we observed that the mice injected with *PKIB* knockdown T24 cells developed fewer metastatic lung nodules compared to those injected with control cells (Figs. 7B, C). We also established a subcutaneous mouse model by subcutaneously injecting BALB/c nude mice with 5637 cells of stable *PKIB* knockdown (Fig. 7D). The tumor tissue volume in mice engrafted with *PKIB*-knockdown cells was considerably smaller than that in control mice (Figs. 7E, F). Indeed, the tumor growth rate was decreased by *PKIB* knockdown (Fig. 7G). This was further confirmed by immunohistochemical (IHC) staining of tumor tissues from sacrificed mice, which showed lower expression of Ki-67 in *PKIB*-deficient tumor tissues than in control tissues. Strikingly, IHC staining also indicated that *PKIB* knockdown markedly increased the expression of p-HSP27-S15, p-HSP27-S78 and p-HSP27-S82 in tumors (Figs. 7H, I), suggesting that PKIB mediates the phosphorylation of HSP27 in vivo. To investigate the inhibitory effects of HSP27 phosphorylation on BLCA metastasis, we also conducted a complementary in vivo experiment. The results demonstrated that HSP27 phosphorylation effectively suppressed tumor metastasis in animal models (Fig. S6), which is consistent with our proposed mechanistic hypothesis regarding its tumor-inhibitory function. Taken together, these findings are consistent with our in vitro results and further support the conclusion that PKIB mediates HSP27 phosphorylation and plays an important role in promoting BLCA metastasis and proliferation.

### Gemcitabine inhibits the proliferation of BLCA cells via the MYCN-PKIB-HSP27 axis

Gemcitabine is a well-established chemotherapeutic agent used in clinical trials [51, 52]. It has been reported to activate the PKA signaling pathway [53] and induce HSP27 phosphorylation in pancreatic cancer [53, 54]. To investigate whether the anticancer effect of gemcitabine in BLCA involves the MYCN-PKIB-PKA-HSP27 axis, we detected the protein expression of MYCN, PKIB, and HSP27 in gemcitabine-treated BLCA cells. T24 and 5637 cells were first treated with 10 nM gemcitabine for 24 h. As expected, these treatments significantly inhibited the proliferative capacity of both types of BLCA cells and induced cell death (Fig. 7J). We then conducted time-dependent experiments on gemcitabine-treated T24 and 5637 cells to detect the protein levels of MYCN, PKIB, and p-HSP27. Interestingly, as the duration of drug exposure increased, there were notable changes in protein expression. In particular, the expression levels of the MYCN and PKIB proteins decreased over time, while those of p-HSP27-S15, p-HSP27-S78 and p-HSP27-S82 all increased gradually. Importantly, no changes in HSP27 protein expression were observed in the gemcitabine-treated cells (Fig. 7K). These results imply that the anticancer mechanism of gemcitabine may be mediated through the signaling axis involving MYCN-PKIB-PKA-HSP27 in BLCA.

In conclusion, our data suggest that high expression of PKIB, regulated by MYCN, mediates reduced HSP27 phosphorylation through inhibition of PKA kinase activity, thereby inducing a malignant phenotype in human bladder cancer (Fig. 7L).

## Discussion

Increasing evidence suggests that dysregulation of kinase/phosphatase signaling is a frequent pathological characteristic of tumors. Dysregulated cAMP–PKA signaling is frequently regarded as crucial for mediating various physiological and pathological effects associated with malignant tumors [3, 9]. The results presented in this manuscript provide critical molecular and biological insights into the role of the *PKIB*, an endogenous PKA inhibitor, in tumors, as an oncogene and a driver of tumor cell proliferation and invasion in BLCA. Protein kinase inhibitors (PKIs) are important regulators of intracellular signaling pathways that control cell growth and division. These proteins typically bind to PKA via pseudosubstrate sequences and act as negative regulators of PKA kinase activity. Three PKIs (PKIA, PKIB and PKIG) have been discovered [44], among which the role of PKIA in PKA has been widely reported [28, 55]. Notably, the PKIs are endogenous, potent, heat-stable, and highly specific PKA inhibitors that effectively blocks PKA-mediated substrate phosphorylation, representing one of the most efficient strategies for PKA inhibition and a potential therapeutic target in diseases with aberrant PKA signaling.

Although PKIB exhibits only 40% amino acid homology with PKIA, its binding interactions and inhibitory effects on PKA remain inadequately characterized [28]. In our study, we found that the expression of only *PKIB* was significantly upregulated in BLCA, suggesting that PKIB plays an important role in bladder tumors. Although *PKIB* is abnormally overexpressed in various cancers and promotes tumorigenesis, the detailed molecular mechanisms by which PKIB regulates tumor progression are unclear. To the best of our knowledge, the molecular mechanism of PKIB in tumors is mostly understood in prostate cancer, where PKIB promotes the growth and invasion of prostate cancer cells by increasing the phosphorylation of AKT [29]. However, these findings imply that PKIB enhances the activity of PKA kinase in prostate cancer, which contradicts the role of PKIs as pseudosubstrates for PKA. More importantly, there are currently no reports indicating that AKT is a substrate of PKA. If PKIB enhances the phosphorylation of AKT via PKA, the substrate of PKIB-mediated PKA kinase has yet to be identified. The impact of PKA on cancer cell proliferation, invasion, and metastasis may be attributed to its interaction with multiple substrates. The diverse functions of PKA are likely due to its numerous targets, each contributing to its multifaceted roles in cancer progression. In our work, we found that PKIB plays an important oncogenic role in BLCA by inhibiting PKA kinase activity and reducing the phosphorylation of a new substrate target protein, HSP27, which confirms the biological function of PKIB as a specific PKA inhibitor. *PKIB* overexpression is a common feature in multiple malignancies, suggesting that this PKIB-PKA-HSP27 regulatory axis may have broader implications across different cancer types. On the other hand, our findings indicate that PKIB plays a role in the nuclear translocation of PKA-Cα, either by promoting its nuclear import or inhibiting its export, aligning with observations in prostate cancer [29]. Unlike PKIA, which promotes PKA-C nuclear export through binding with its pseudosubstrate motif [55], PKIB’s effect on PKA-C is comparatively less pronounced despite sharing the same motif [28]. The intracellular translocation of PKIB involved in PKA-C appears to be different from that of PKIA.

HSP27 is a small molecular chaperone whose activity is regulated through dynamic phosphorylation. In humans, HSP27 is phosphorylated at three primary sites: Ser15, Ser78, and Ser82 [46]. This phosphorylation is mediated by various kinases, reflecting a complex interplay that varies with cell type and signaling context [45]. Key kinases involved in HSP27 phosphorylation include MAPK-activated protein kinases (MK2, MK3, MK5) and Protein Kinase D (PKD) [56]. MK2 and MK5 are particularly notable for their role in directly phosphorylating HSP27, impacting its oligomerization and stress response functions [45]. Although Protein Kinase A (PKA) has been reported to phosphorylate HSP27 in some studies [57–60], this association is contentious and may be indirect [61–64], involving intermediary kinases such as MK2 or MK5 [45]. The consensus in the field suggests that while MK2, MK5, and PKD are crucial for HSP27 phosphorylation, the role of PKA remains ambiguous and is likely dependent on cellular context. In this study, however, through protein interaction analysis (including GST pull-down assay, Co-IP assay and immunofluorescence) and conformational dynamics study (molecular docking and molecular dynamics simulation), we demonstrated that HSP27 is a direct substrate of PKA kinase in BLCA cells. We determined that PKA binds to the NTD domain of HSP27 and phosphorylates its S15, S78, and S82 serine residues. In addition, phosphoproteomic analysis and *PKIB* knockdown or overexpression also confirmed that the phosphorylation of HSP27 by PKA is regulated by PKIB. To our knowledge, this is the first clear evidence that HSP27 is a direct substrate of PKA. Previous reports have shown that both phosphorylated and nonphosphorylated forms of HSP27 are predominantly localized in the cytoplasm [65], and our immunofluorescence staining demonstrated similar results. PKIB is expected to interact with PKA through two primary mechanisms: it both inhibits the kinase activity of the PKA catalytic subunit and modulates its nuclear localization. These mechanisms operate independently, and PKIB might employ both pathways to modulate HSP27 phosphorylation in BLCA.

The tumorigenic and metastatic roles of HSP27 have been well described [46]. HSP27 exists in two distinct forms, characterized by their phosphorylation and oligomerization states, each with specific functions and subcellular localizations. The phosphorylated monomeric HSP27 is primarily engaged in signaling pathways within the cytoplasm and nucleus, while the multimeric (nonphosphorylated) form is mainly involved in protein folding and preventing aggregation [66]. Increased expression and phosphorylation of HSP27 have been correlated with various tumor features, including disease progression, metastatic potential, therapeutic resistance, and adverse prognosis [67]. Although the relevance of HSP27 in tumor biology is undeniable, the evidence for its expression level and clinical significance in bladder tumors is still controversial [68]. The role of this molecule in BLCA has not been clearly elucidated. In this study, we found that phosphorylated HSP27 inhibited BLCA cell viability and invasion, whereas overexpression of HSP27 (nonphosphorylated) had no effect on these phenotypes. Moreover, phosphorylation at S15 of HSP27 plays a more important role in inhibiting BLCA cells than phosphorylation at S78 or S82. This finding is also in line with the results of PKA-mediated phosphorylation assays in HSP27 nonphosphorylated mutants, with the greatest reduction in phosphorylation levels occurring in the S15A mutant. In addition, HSP27-3D rescued the proliferation, migration and invasion phenotypes of BLCA cells enhanced by *PKIB* overexpression, suggesting that the carcinogenic effect of PKIB is mediated by altering HSP27 phosphorylation. Furthermore, phosphorylated HSP27 reduces the phosphorylation of AKT at S473 and inhibits its activity in BLCA, which is consistent with previously reported results showing a direct interaction between HSP27 and AKT [48]. Collectively, these data demonstrate that HSP27’s role in BLCA is phosphorylation-state-specific, with S15 phosphorylation being functionally dominant. Our findings reveal that PKIB-mediated inhibition of HSP27 phosphorylation represents a pivotal mechanism in BLCA progression, providing new insights into context-dependent PKA signaling regulation.

Although *PKIB* has been reported to be upregulated in a variety of cancers, the regulatory mechanisms leading to its abnormal overexpression are unknown. In this study, we screened MYCN, a transcription factor that is positively associated with *PKIB* expression in BLCA, and demonstrated that MYCN directly binds to two sites in the *PKIB* promoter region and promotes its transcription. MYCN is a pivotal oncogene functioning as a DNA-binding transcription factor that interacts with the bHLH domain of the E box promoter region to regulate gene expression [69]. Dysregulation of MYCN is implicated in various malignancies, influencing tumor initiation, progression, and therapeutic response [70, 71]. Evidence highlights MYCN’s role in neuroblastoma, where it modulates cell proliferation and apoptosis through its interaction with the transcriptional target NLRR1 [72]. In prostate cancer, MYCN enhances tumor progression by transcriptionally activating genes such as PARP1 and BRCA1 [73]. Additionally, MYCN has been shown to contribute to chemoresistance in non-small cell lung cancer (NSCLC) by binding to the *HES1* promoter, which suppresses apoptotic pathways. Elevated MYCN levels are associated with advanced NSCLC stages and poorer patient survival [74]. Recent findings in BLCA also indicate that *MYCN* is upregulated and regulates cell proliferation through interaction with circLAMA3 [75]. Our study further demonstrates that MYCN upregulates *PKIB* promoter activity, underscoring its critical role as a regulator of PKIB in BLCA.

To date, the oncogenic molecular mechanism of PKIB in various cancers has not been determined. The results of our study suggest that MYCN promotes *PKIB* overexpression in BLCA, thereby inhibiting PKA activity and reducing HSP27 phosphorylation. This potentially represents a novel mechanism by which the MYCN-PKIB-PKA-HSP27 axis regulates BLCA tumorigenesis. Interestingly, we also found that the chemotherapeutic agent gemcitabine has a significant effect on this axis. After treating BLCA cells with gemcitabine, the expression of MYCN and PKIB decreased over time, while the expression of p-HSP27 increased gradually, consistent with the inhibitory effects of these protein expression changes on cancer cells. It has been reported that gemcitabine activates the PKA signaling pathway [53] and induces the phosphorylation of HSP27 [54] in pancreatic cancer. Additionally, gemcitabine mediates pancreatic cancer cell growth inhibition by activating the p38 MAPK-MAPKAP-2 pathway, which results in the phosphorylation of HSP27 [76]. In BLCA, the inhibition of HSP27 (OGX-427, an antisense oligonucleotide targeting HSP27) has been reported to enhance cell susceptibility to gemcitabine [77]. Gemcitabine is a cell cycle phase-specific chemotherapeutic agent (pyrimidine antimetabolite) whose mechanism of action involves killing cells undergoing deoxyribonucleic acid (DNA) synthesis (in the S phase), and it also impedes cell transition across the G1/S phase boundary [78]. Gemcitabine is the standard first-line therapy for BLCA; however, the prevalence of gemcitabine resistance significantly impacts long-term patient survival [79]. Over the years, resistance to gemcitabine in BLCA has been largely understudied. Therefore, developing a more complete understanding of gemcitabine and establishing a rational basis for the design of effective approaches for treating gemcitabine resistance remain major clinical challenges. The response of the MYCN, PKIB and p-HSP27 proteins to gemcitabine found in our study implies that the inhibitory effect of gemcitabine on BLCA may be mediated through the MYCN-PKIB-PKA-HSP27 axis, which may provide new implications for the clinical treatment of BLCA.

Recently, several comprehensive genomic analyses of bladder cancers have provided detailed characterization of genetic and epigenetic alterations in BLCA subtypes [80–86]. While classification systems differ in subtype nomenclature and numbers, luminal and basal subtypes consistently show the greatest separation, with distinct expression profiles, differentiation patterns, oncogenic mechanisms, tumor microenvironments, and associated histological and clinical features [87]. Although none of the current numerous molecular subtypes criteria for BLCA uses *PKIB* gene as a subtype-specific marker, we also conducted a preliminary study on the oncogenic role and molecular mechanism consistency of PKIB in the major BLCA subtypes. We performed bioinformatic analyses of *PKIB* gene expression in several transcriptome datasets (GSE87304 and GSE124305) with molecular subtype information [88, 89] and in the vast majority [25] of BLCA cell lines from DepMap (Broad Institute Cancer Dependency Map) [90]. The results showed that there was no significant difference in the expression of the gene among different molecular subtypes (Fig. S7A–D). We also selected eight commonly used BLCA cell lines among three major subtypes [90] (RT4, SW780 and RT112 belong to luminal subtype, SCaBER, HT1376 and 5637 belong to basal subtype, T24 and J82 belong to non-type or mix subtype) for qRT-PCR to further confirm the results. There was also no significant difference in *PKIB* expression among these subtypes (Fig. S7E, F). Furthermore, *PKIB* knockdown significantly inhibited EMT, migration, invasion, and proliferation of 5637 (basal subtype), T24 (mixed subtype), and RT112 (luminal subtype) cells (Fig. 2 and Fig. S1), suggesting that the oncogenic role of PKIB in different subtypes of BLCA cells is conserved. In addition, phosphorylation levels at S15, S78 and S82 of HSP27 were significantly elevated in T24, RT112 and 5637 cells with *PKIB* knockdown (Fig. 3P and Fig. S3C, D), implying that PKIB-mediated phosphorylation of downstream target HSP27 is consistent in BLCA. These results provide a preliminary indication that although there are some differences in clinical and histological behavior and markers of BLCA subtypes, *PKIB* expression, oncogenic role and targets are similar across the major subtypes (basal, luminal, and mixed), indicating the conserved nature of PKIB in BLCA.

The role of PKIB remains not fully elucidated. While prior researches have investigated PKI’s impacts on specific kinases such as protein kinase G (PKG) and protein kinase C (PKC) [91–94], the effects on a wider range of kinases have yet to be thoroughly explored. It remains uncertain whether PKIB exclusively inhibits PKA or if it also affects other enzymes or proteins. On the basis of our proteomic analysis of *PKIB*-knockdown T24 cells, we identified a number of significantly up- or downregulated proteins, the vast majority of which are not known direct substrates of PKA kinase. Does PKIB alter the phosphorylation of these proteins by affecting the activity of other kinases or by other regulatory means? On the other hand, although *PKIA* and *PKIG* show no significant overexpression in BLCA, their potential functional redundancy with PKIB is worth investigating, especially considering the link between PKA inhibition and BLCA progression [40–43].While no pharmacological PKIB inhibitors currently exist, microRNA-495 has been shown to induce *PKIB* silencing [95], providing a potential tool for probing PKIB interactions.

In summary, this study elucidates the oncogenic role of PKIB and uncovers a novel MYCN-PKIB-PKA-HSP27 signaling axis in bladder cancer (BLCA), advancing our understanding of PKA-mediated regulatory mechanisms in tumorigenesis. Furthermore, our findings highlight PKIB’s potential as a therapeutic target and its unique function as an endogenous PKA-specific inhibitor, offering valuable translational implications for cancer research and targeted therapy.

## Material and methods

### Cell culture and reagents

Human bladder cancer cell lines T24, 5637, RT112 and human embryonic kidney 293 T (HEK293T) cell lines were obtained from the Cell Bank of the Chinese Academy of Sciences. Each cell line was then subjected to STR-based identification and was not contaminated by mycoplasma. All cell lines were used for less than half a year within 25 passages. HEK293T cells were grown in DMEM (Procell, PM150213), 5637 and RT112 cells were grown in RPMI 1640 (Procell, PM150110), T24 cells were grown in McCoy’s 5 A (Procell, PM150710). The medium was supplemented with 10% fetal bovine serum (FBS) (Gibco, 1099-141). The cells were cultured at 37 °C in a humidified 5% CO2 incubator.

### Transfection and lentiviral infection

Following the manufacturer’s protocol, short interfering RNAs (siRNAs) or plasmids were transiently introduced into cells using Lipofectamine 3000 (Invitrogen), with subsequent analysis performed 48 h post-transfection. Primer sequences for plasmid construction and siRNA sequences are detailed in Supplementary Table 1. For stable gene overexpression or knockdown, cells were infected with lentivirus from GenePharma Biotech (Shanghai) for 72 h and then selected with 2 μg/ml puromycin to establish stable cell lines.

### Western blot analysis

Cell protein was extracted using RIPA lysis (Beyotime Biotechnology, P0013B) supplemented with phosphatase inhibitor (Sangon Biotech, C500017) and protease inhibitor (Sangon Biotech, C600387) and was boiled with native sample loading buffer (Sangon Biotech, C506032) and DTT (Sangon Biotech, B645939) at 95 °C for 10 min. Then protein was subjected to electrophoresis with SDS-containing polyacrylamide gels, and was transferred to activated polyvinylidene fluoride (PVDF) membrane (Millipore). After 2 hours of blocking with 3% BSA, the membrane was incubated with the primary antibody overnight at 4 °C. TBST buffer was used for washing three times for 10 min each. The appropriate HRP-conjugated secondary antibody was selected for incubation for 1 hour at room temperature. Finally, using a chemiluminescent HRP substrate (Fdbioscience), the membrane was visualized with an enhanced chemiluminescence detection system. The following antibodies were used for Western blotting: rabbit anti-PKIB (Abcam, ab233521), anti-HSP27 (Abcam, ab109376), anti-HSP27 S15 (Abcam, ab76313), anti-HSP27 S78 (Abcam, ab32501), anti-HSP27 S82 (Abcam, ab155987), anti-MYCN (Abcam, ab227822), anti-HA (Abcam, ab236632), anti-FLAG (Abcam, ab205606), anti-PKA C-α (Cell Signaling Technology, #4782), anti-AKT S473 (Cell Signaling Technology, #4060), anti-AKT (Cell Signaling Technology, #4691), anti-Lamin B1 (Cell Signaling Technology, #13435), anti-β-actin (ABclonal, #AC004), anti-phospho-serine (Bioss, bs-11993R), mouse anti-N-cadherin (BD Biosciences, 610920), and anti-Vimentin (BD Biosciences, 550513).

### RNA extraction and real-time quantitative reverse transcriptase PCR (qRT–PCR)

Total RNA was extracted from cells or tissues using TRIzol (Invitrogen, 10296010) and converted to cDNA using HiScript II Q RT SuperMix (Vazyme, R223-01). qRT‒PCR experiments were performed in triplicate. To calculate the relative expression of *PKIA, PKIB*, and *PKIG*, the data were analysed using the ΔΔCt method. For each gene, the mRNA expression was unified to that of β-actin.

### Co-immunoprecipitation

The cells were cultured on 10-cm dishes and lysed in IP lysis buffer (Thermo Fisher Scientific, 87788) for 20 min. Fifteen percent of the lysates was reserved as the input control. The left lysates were mixed with antibodies against FLAG (Abcam, ab205606), HA (Abcam, ab236632), PKA (CST, #4782), HSP27 (Abcam, ab109376) or IgG (CST, #3900) and rotated overnight at 4 °C. To capture the immune complexes, protein A/G magnetic beads (Thermo Fisher Scientific, 88802) were used and rotated at room temperature for 1 h. After being washed 3 times, the protein was boiled with protein loading buffer at 95 °C for 10 min, separated from the magnetic beads and was subjected to further Western blot analysis.

### CCK-8 assay

In total, 2000 cells were plated in 100 μl medium in a 96-well plate per well. After culturing the cells for 24, 48, 72, 96 and 120 h, 10 μl of CCK-8 solution (Vazyme, A311, China) was added to each well and incubated for 2 h, after which the absorbance was measured at 450 nm.

### Transwell assay of migration and invasion

For the migration assay, 600 μl serum-free medium was first added under the bottom of chamber in a 24-well plate (Corning, 353504), and 40,000 cells in 200 μl medium supplemented with 25% FBS were added to the upper chamber of transwell inserts (Corning, 353097). After cultivation for 48 h, the cells on the membrane were washed twice with PBS buffer, fixed with methanol for 30 min and stained with crystal violet. Finally, the cells on the upper surface were removed, and the positively stained cells were observed under an inverted microscope. For the invasion assay, Matrigel Matrix (Corning, 354248) was initially added on the upper chamber and incubated at 37 °C for 30 min.

### Cell cycle assay

After digestion with trypsin solution (Beyotime Biotechnology, C0207), the cells were washed twice with cold PBS buffer and fixed with 75% ethanol for 24 h at 4 °C. Subsequently, the cells were washed twice with PBS buffer again and incubated with propidium iodide (PI) (Beyotime Biotechnology, C1052) at 37 °C for 30 min. The cell cycle was detected by flow cytometry (Beckman Coulter).

### Wound-healing assay

T24 and 5637 cell lines were seeded in 6-well plates until about 80–90% confluence. Then, a 100-μl pipette tip was used to make straight scratches. The cells were cultured in 0% serum medium for another 24 h. Images of cells were taken in three random microscopic fields at 0 h and 24 h.

### Chromatin immunoprecipitation (ChIP)

EZ CHIP KIT 22 ASSAYS (Millipore, 17-371RF) was used for the ChIP assay according to the manufacturer’s instructions. Briefly, T24 cells were grown on 6-cm dishes and fixed with 1% formaldehyde for 10 min at room temperature. Glycine was then added to quench the unreacted formaldehyde. After being washed twice with PBS buffer and lysed with SDS buffer, the nuclear pellet was collected and sonicated to yield cross-linked DNA fragments 400–1000 bp in length. Chromatin was incubated with anti-MYCN antibody and Protein G magnetic beads overnight at 4 °C. Mouse IgG antibody (ab190475) was used as a negative control. The DNA- protein complexes were eluted and decrosslinked to collect ChIP DNA. The DNA was subjected to PCR amplification. The primers used for ChIP-PCR are listed in Supplementary Table 1.

### GST pull-down assay

The GST-HSP27 fusion protein was induced in *E. coli* BL21 and purified using a GST 4FF chromatographic column (Sangon Biotech, C600912) according to the manufacturer’s instructions. FLAG-PKA fusion protein was expressed in 293 T cells and purified using anti-FLAG magnetic beads (Bimake, B26102). Then, the GST-HSP27 fusion protein or GST protein was incubated with anti-FLAG magnetic beads overnight at 4 °C. The GST fusion proteins were subjected to SDS‒PAGE and confirmed by Coomassie blue staining. The bound proteins were detected by Western blotting.

### In vitro phosphorylation assay

In total, 80 μl recombinant GST-HSP27 product from *E. coli* BL21 was added with 0.3 μg purified FLAG-PKA Cα protein in kinase buffer (35 mM Tris-HCl, pH 7.5; 10 mM MgCl2; 0.5 mM EGTA; and 0.2 mM CaCl2) with 200 μM ATP for 30 min at 30 °C in a final volume of 100 μl. Proteins were subjected to Western blotting after the reactions were stopped at 99 °C for 5 min.

### PKA kinase activity assay

For some experiments, T24 cells were pretreated with 10 μM H89 (Selleck, S1582) for 1 h. After being washed with PBS buffer for 2 times, the cells were lysed with RIPA buffer. According to the manufacturer’s instructions, an ELISA was used to measure PKA activity (Arbor Assays, k027-h1). The absorbance was measured at 450 nm. The PKA concentration (U/ml) was calculated with an internal calibration curve.

### Immunofluorescence staining

T24 cells were cultured on round coverslips, fixed for 20 min with 4% paraformaldehyde, washed 3 times with PBS buffer and incubated with 3% BSA in PBST buffer for 1 h. Following incubation with rabbit anti-PKA C-alpha (Cell Signaling Technology, #4782) and mouse anti-HSP27 (Santa Cruz, sc-13132) antibodies overnight at 4 °C. After being washed, the cells were incubated with goat anti-rabbit IgG H&L (FITC) antibody (Abcam, ab6716) and goat anti-mouse IgG H&L (Cy3) antibody (Abcam, ab97035) at room temperature for 1.5 h. Cells nuclei were stained with DAPI (Beyotime Biotechnology, C1006). Finally, images were captured using a confocal laser microscope (Leica).

### Colony formation assay

The treated bladder cancer cells were seeded in a 6-well plate (1500 cells per well) and cultivated for approximately 3 weeks. Cells were then fixed for 20 min with 1 ml methanol and stained for 30 min with crystal violet.

### Dual-luciferase reporter assay

To determine the effect of MYCN on the luciferase activity of the *PKIB* promoter, the sequences of the *PKIB* promoter and MYCN were constructed and inserted into the pGL3-Luc reporter vector and pCDNA3.1-HA vector, respectively. The plasmids were then co-transfected into HEK293T cells for 48 h, and a Dual-Luciferase Reporter Assay System (Promega) was used for luciferase reporter assays. The relative luciferase activity was unified to the corresponding Renilla luciferase activity.

### RNA-seq analysis

The mRNA expression profiles of *HSP27*-overexpressing and HSP27-overphosphorylating T24 cells were determined via transcriptome sequencing on an Illumina HiSeq platform (Origingen). Briefly, total RNA was extracted, quantified and qualified using TRIzol Reagent (Invitrogen), TBS380 PicoGreen (Invitrogen) and NanoDrop spectrophotometer (Thermo Fisher Scientific). Sequencing libraries were prepared with the TruSeq RNA Sample Preparation Kit and then were loaded onto an Illumina HiSeq instrument, and sequenced using 2 × 150 bp paired-end reads. HISAT2 software was used to map the reads to the human genome (GRCh38). Differentially expressed genes (DEGs) with a *P* value < 0.05 and a fold change (FC) > 2 were chosen for further investigation. The cells in each group were analysed in triplicate.

### Phosphopeptide correlation profiling

Phosphopeptide correlation profiling was performed for three biological replicates at Shanghai GenePharma Biotech. Briefly, 100 μg of protein was combined with 15 μl of SDT buffer. Subsequently, DTT, the detergent and other low-molecular-weight components were removed by two rounds of ultrafiltration (Sartorius, 30 kD) using UA buffer. Next, 50 μl of iodoacetamide was added for 30 minutes in darkness. The membranes were washed three times with UA buffer and subsequently washed two times with TEAB buffer. The proteins were digested with 4 μg of trypsin (Promega) in TEAB buffer overnight at 37 °C. The peptide mixture (100 μg) was labeled with TMT reagent (Thermo Fisher Scientific). Subsequently, the labeled peptides were subjected to desalting using a C18 cartridge and was subjected to enrichment for phosphopeptides. The pooled TiO2 flow-through (FT) and wash fractions were combined, and further phosphopeptide enrichment was carried out. The eluents from both the TiO2 and Fe-NTA processes were dried and subsequently reconstituted in formic acid buffer. LC‒MS/MS analysis was conducted using a mass spectrometer coupled to an Easy nLC system (Thermo Fisher Scientific) for 4 h. Phosphopeptides exhibiting a fold change >1.2 and a *P* value (Student’s *t* test) <0.05 were considered differentially expressed phosphorylated peptides.

### Structural modeling, molecular docking and molecular dynamics simulation

The protein homology models for human PRKACA (PKA-Cα) and HSP27 were constructed by using the MODELLER V9.19 [96] platform and templates derived from the protein data bank (www.rcsb.org) with PRKACA (4W5B) and HSP27 from their respective Homo 24-mer structure (6DV5, chain A), respectively, with very slow refinement. The quality of the modeled structure was evaluated by PROCHECK [97]. The PRKACA and HSP27 complex was docked using ZDOCK 2.3.2 [98, 99]. Protein complexes were ranked using a scoring function that included pairwise shape complementarity, desolvation and electrostatic terms (for quality scoring, see [100, 101]). The constructed complex was then subjected to MD simulations to study the conformational dynamics. The non-bonded interaction potential was terminated in a smooth and controlled manner at 10–12 Å. Furthermore, the Coulomb interactions were treated using the particle-mesh Ewald method [102]. The LINCS algorithm [103] was employed to impose constraints on the hydrogen-containing bonds. The minimized structure was then heated gradually from 0 to 303.15 K over 100 ps, followed by a two-step equilibration process (each 100 ps). The temperature was initially set at 303.15 K. Following this, the system was subjected to an equilibration process at 1 atm, utilizing the Parrinello-Rahman barostat [104]. The system was then subjected to 500 ns of MD production using the GPU-enabled version of the GROMACS software package (version 2021.4) [105] with the AMBER99SB-ILDN force field [106] with an integration step of 2 fs. The GROMACS analysis toolkit was employed to analyse MD trajectories, with root mean square deviations (RMSDs) subsequently calculated.

### Bioinformatic analysis of public datasets

The TCGA_BLCA dataset containing 408 BLCA and 19 normal tissues from the Genomic Data Commons (GDC) database (https://gdc.cancer.gov/) was downloaded to detect the mRNA expression of PKIs. The GSE13507 dataset from the Gene Expression Omnibus (GEO) database (https://www.ncbi.nlm.nih.gov/geo/) was downloaded, and the KEGG Rest API (https://www.kegg.jp/kegg/rest/keggapi.html) was constructed with clusterProfiler (version 3.14.3) in the R language to screen for upregulated pathways in BLCA tissues overexpressing PKIB. Differentially expressed genes (DEGs) with an FDR < 0.25, an abs. log2FC > 1 and an adjusted *P* value < 0.01 were considered to be significantly different in the GEO database.

### Animal experiments

All animal procedures were approved by the Animal Care and Use Committee of Soochow University. Male BALB/c nude mice, aged 4–6 weeks, were procured from the Laboratory Animal Center of Suzhou Medical College, Soochow University. To minimize potential bias, all animals were randomly allocated to experimental groups, and investigators remained blinded to group assignments throughout data collection and analysis. No sex-related differences were observed in any experimental outcomes. For the xenograft studies, sample sizes were determined based on established protocols from previous experiments rather than predetermined statistical methods.

For the in vivo metastasis assay, *PKIB*-silenced and control T24 cells (2×10^6^) in 0.15 ml PBS were injected intravenously into the tail vein of mice. For macroscopic analysis of metastatic nodules, mice were euthanised after 8 weeks of inoculation and their lungs were fixed in Bouin’s solution. Lung tissues were histologically examined using H&E staining for micrometastatic foci analysis.

For the in vivo proliferation assay, *PKIB*-silenced and control 5637 cells (3×10^6^) in 0.15 ml of PBS were injected subcutaneously into the axilla of the mice. Every four days, a vernier calliper was used to measure the tumor formed by the cells. Tumor volume (V) was estimated by measuring the longest diameter (L) and the shortest diameter (W) of the tumor. V = 0.5×W^2^×L.

### Human BLCA tissue specimens

Fresh BLCA tumor and corresponding paracarcinoma tissue specimens were obtained after informed consent from 296 patients with BLCA authorized by the Second Affiliated Hospital of Soochow University (Suzhou, China) according to the principles of the Declaration of Helsinki. Detailed clinical information of the patients is listed in Tables S2, S3, S4, S6. None of patients received chemotherapy or radiotherapy before surgical operation. The study was approved by the Academic and Ethics Advisory Committee of the Second Affiliated Hospital of Soochow University (JD-LK-2019-104-01).

### Tissue microarray (TMA) analysis

The TMA slides were systematically evaluated through dual-pathologist review, with all tissue cores (1.5 mm diameter, each representing an individual BLCA patient) being independently scored by two experienced pathologists. For both PKIB expression and HSP27-Ser15 phosphorylation status, immunohistochemical staining intensity was semi-quantitatively assessed using a four-tiered grading system: 0 (Negative): No detectable staining; 1 (Weak): Faint yellow cytoplasmic signal; 2 (Moderate): Distinct brownish-yellow coloration; 3 (Strong): Intense dark brown deposition.

### Immunohistochemistry (IHC) analysis

Tissues from the indicated mice were isolated, rinsed in PBS, fixed in 10% neutral buffered formalin for 24 h, dehydrated, and embedded in paraffin. Tissue sections of 5 mm thickness were deparaffinized, rehydrated, and then treated for antigen retrieval. After blocking in protein blocking buffer, tissues were incubated with primary antibody overnight at 4 °C followed by incubation with biotinylated secondary antibody. Endogenous peroxidase was quenched in 3% H_2_O_2_ in water for 10 min at room temperature. Antibodies were visualized with avidin-biotin complex using diaminobenzidine as the chromogen.

### Statistical analysis

All the data were analyzed using GraphPad Prism 5.02 software. For statistical significance between two groups, an unpaired Student’s *t* test was applied. Variance between groups was tested by one-way analysis of variance (ANOVA) with Tukey’s multiple comparison tests. All the data in the graphs are presented as the means ± SDs from at least three independent experiments except where otherwise stated. *P* < 0.05 was considered to indicate statistical significance (*P < 0.05; **P < 0.01; ***P < 0.001; ns, not significant).

## Supplementary information

Supplementary materials

Supplementary Table S5

Supplementary Table S7

Supplementary Table S8

Supplementary original Western blots

## Data Availability

All data generated or analyzed during the course of this study are included in the published article information file, as well as in the supplementary information file.

## References

[1] Kolch W, Halasz M, Granovskaya M, Kholodenko BN. The dynamic control of signal transduction networks in cancer cells. Nat Rev Cancer. 2015;15:515–27. 26289315 10.1038/nrc3983

[2] Gelens L, Qian J, Bollen M, Saurin AT. The importance of kinase-phosphatase integration: lessons from mitosis. Trends Cell Biol. 2018;28:6–21. 29089159 10.1016/j.tcb.2017.09.005

[3] Naviglio S, Caraglia M, Abbruzzese A, Chiosi E, Di Gesto D, Marra M, et al. Protein kinase A as a biological target in cancer therapy. Expert Opin Ther Targets. 2009;13:83–92. 19063708 10.1517/14728220802602349

[4] Tasken K, Skalhegg BS, Tasken KA, Solberg R, Knutsen HK, Levy FO, et al. Structure, function, and regulation of human cAMP-dependent protein kinases. Adv Second Messenger Phosphoprot Res. 1997;31:191–204. 10.1016/s1040-7952(97)80019-5 9344252

[5] Howe AK, Baldor LC, Hogan BP. Spatial regulation of the cAMP-dependent protein kinase during chemotactic cell migration. Proc Natl Acad Sci USA. 2005;102:14320–5. 16176981 10.1073/pnas.0507072102 PMC1242330

[6] Lim CJ, Kain KH, Tkachenko E, Goldfinger LE, Gutierrez E, Allen MD, et al. Integrin-mediated protein kinase A activation at the leading edge of migrating cells. Mol Biol Cell. 2008;19:4930–41. 18784251 10.1091/mbc.E08-06-0564 PMC2575143

[7] Paulucci-Holthauzen AA, Vergara LA, Bellot LJ, Canton D, Scott JD, O’Connor KL. Spatial distribution of protein kinase A activity during cell migration is mediated by A-kinase anchoring protein AKAP Lbc. J Biol Chem. 2009;284:5956–67. 19106088 10.1074/jbc.M805606200 PMC2645839

[8] Reggi E, Diviani D. The role of A-kinase anchoring proteins in cancer development. Cell Signal. 2017;40:143–55. 28927666 10.1016/j.cellsig.2017.09.011

[9] Zhang H, Kong Q, Wang J, Jiang Y, Hua H. Complex roles of cAMP-PKA-CREB signaling in cancer. Exp Hematol Oncol. 2020;9:32. 33292604 10.1186/s40164-020-00191-1 PMC7684908

[10] Kwiecinska P, Ptak A, Wrobel A, Gregoraszczuk EL. Hydroxylated estrogens (2-OH-E2 AND 4-OH-E2) do not activate cAMP/PKA and ERK1/2 pathways activation in a breast cancer MCF-7 cell line. Endocr Regul. 2012;46:3–12. 22329816

[11] Gong S, Chen Y, Meng F, Zhang Y, Wu H, Wu F. Roflumilast restores cAMP/PKA/CREB signaling axis for FtMt-mediated tumor inhibition of ovarian cancer. Oncotarget. 2017;8:112341–53. 29348829 10.18632/oncotarget.22866 PMC5762514

[12] Peverelli E, Giardino E, Mangili F, Treppiedi D, Catalano R, Ferrante E, et al. cAMP/PKA-induced filamin A (FLNA) phosphorylation inhibits SST2 signal transduction in GH-secreting pituitary tumor cells. Cancer Lett. 2018;435:101–9. 30098401 10.1016/j.canlet.2018.08.002

[13] Wang S, Zhang Z, Qian W, Ji D, Wang Q, Ji B, et al. Angiogenesis and vasculogenic mimicry are inhibited by 8-Br-cAMP through activation of the cAMP/PKA pathway in colorectal cancer. Onco Targets Ther. 2018;11:3765–74. 29997437 10.2147/OTT.S164982 PMC6033084

[14] Jiang K, Yao G, Hu L, Yan Y, Liu J, Shi J, et al. MOB2 suppresses GBM cell migration and invasion via regulation of FAK/Akt and cAMP/PKA signaling. Cell Death Dis. 2020;11:230. 32286266 10.1038/s41419-020-2381-8 PMC7156523

[15] Huang F, Ma G, Zhou X, Zhu X, Yu X, Ding F, et al. Depletion of LAMP3 enhances PKA-mediated VASP phosphorylation to suppress invasion and metastasis in esophageal squamous cell carcinoma. Cancer Lett. 2020;479:100–11. 32200035 10.1016/j.canlet.2020.03.014

[16] Chen TC, Hinton DR, Zidovetzki R, Hofman FM. Up-regulation of the cAMP/PKA pathway inhibits proliferation, induces differentiation, and leads to apoptosis in malignant gliomas. Lab Invest. 1998;78:165–74. 9484714

[17] Chen TC, Wadsten P, Su S, Rawlinson N, Hofman FM, Hill CK, et al. The type IV phosphodiesterase inhibitor rolipram induces expression of the cell cycle inhibitors p21(Cip1) and p27(Kip1), resulting in growth inhibition, increased differentiation, and subsequent apoptosis of malignant A-172 glioma cells. Cancer Biol Ther. 2002;1:268–76. 12432276 10.4161/cbt.80

[18] Moon EY, Lee GH, Lee MS, Kim HM, Lee JW. Phosphodiesterase inhibitors control A172 human glioblastoma cell death through cAMP-mediated activation of protein kinase A and Epac1/Rap1 pathways. Life Sci. 2012;90:373–80. 22227470 10.1016/j.lfs.2011.12.010

[19] Cho EA, Kim EJ, Kwak SJ, Juhnn YS. cAMP signaling inhibits radiation-induced ATM phosphorylation leading to the augmentation of apoptosis in human lung cancer cells. Mol Cancer. 2014;13:36. 24568192 10.1186/1476-4598-13-36 PMC4234305

[20] Choi YJ, Kim SY, Oh JM, Juhnn YS. Stimulatory heterotrimeric G protein augments gamma ray-induced apoptosis by up-regulation of Bak expression via CREB and AP-1 in H1299 human lung cancer cells. Exp Mol Med. 2009;41:592–600. 19381065 10.3858/emm.2009.41.8.065 PMC2739899

[21] Chen Y, Sabatini BL. The Kinase Specificity of Protein Kinase Inhibitor Peptide. Front Pharm. 2021;12:632815. 10.3389/fphar.2021.632815 PMC7878667 33584320

[22] Johnson JL, Yaron TM, Huntsman EM, Kerelsky A, Song JH, Regev A, et al. An atlas of substrate specificities for the human serine/threonine kinome. Nature. 2023;613:759. 36631611 10.1038/s41586-022-05575-3 PMC9876800

[23] Musket A, Moorman JP, Zhang JY, Jiang Y. PKIB, a novel target for cancer therapy. Int J Mol Sci. 2024;25:4664. 10.3390/ijms25094664 PMC11083500 38731883

[24] Zhang HY, Liu YL, Liu JY, Chen JZ, Wang J, Hua H, et al. cAMP-PKA/EPAC signaling and cancer: the interplay in tumor microenvironment. J Hematol Oncol. 2024;17:5. 10.1186/s13045-024-01524-x PMC10792844 38233872

[25] Taylor SS, Kim C, Vigil D, Haste NM, Yang J, Wu J, et al. Dynamics of signaling by PKA. Biochim Biophys Acta. 2005;1754:25–37. 16214430 10.1016/j.bbapap.2005.08.024

[26] Ashby CD, Walsh DA. Characterization of the interaction of a protein inhibitor with adenosine 3’,5’-monophosphate-dependent protein kinases. I. Interaction with the catalytic subunit of the protein kinase. J Biol Chem. 1972;247:6637–42. 4342607

[27] Demaille JG, Peters KA, Fischer EH. Isolation and properties of the rabbit skeletal muscle protein inhibitor of adenosine 3’,5’-monophosphate dependent protein kinases. Biochemistry. 1977;16:3080–6. 196623 10.1021/bi00633a006

[28] Dalton GD, Dewey WL. Protein kinase inhibitor peptide (PKI): a family of endogenous neuropeptides that modulate neuronal cAMP-dependent protein kinase function. Neuropeptides. 2006;40:23–34. 16442618 10.1016/j.npep.2005.10.002

[29] Chung S, Furihata M, Tamura K, Uemura M, Daigo Y, Nasu Y, et al. Overexpressing PKIB in prostate cancer promotes its aggressiveness by linking between PKA and Akt pathways. Oncogene. 2009;28:2849–59. 19483721 10.1038/onc.2009.144

[30] Dou P, Zhang D, Cheng Z, Zhou G, Zhang L. PKIB promotes cell proliferation and the invasion-metastasis cascade through the PI3K/Akt pathway in NSCLC cells. Exp Biol Med (Maywood). 2016;241:1911–8. 27325557 10.1177/1535370216655908 PMC5068460

[31] Wan R, Yang G, Liu Q, Fu X, Liu Z, Miao H, et al. PKIB involved in the metastasis and survival of osteosarcoma. Front Oncol. 2022;12:965838. 36072791 10.3389/fonc.2022.965838 PMC9441607

[32] Dabanaka K, Chung S, Nakagawa H, Nakamura Y, Okabayashi T, Sugimoto T, et al. PKIB expression strongly correlated with phosphorylated Akt expression in breast cancers and also with triple-negative breast cancer subtype. Med Mol Morphol. 2012;45:229–33. 23224602 10.1007/s00795-011-0565-0

[33] Zhang JB, Song W, Wang YY, Liu MG, Sun MM, Liu H. Study on correlation between PKIB and pAkt expression in breast cancer tissues. Eur Rev Med Pharm Sci. 2017;21:1264–9. 28387904

[34] Dahlman-Wright K, Qiao Y, Jonsson P, Gustafsson JA, Williams C, Zhao C. Interplay between AP-1 and estrogen receptor alpha in regulating gene expression and proliferation networks in breast cancer cells. Carcinogenesis. 2012;33:1684–91. 22791811 10.1093/carcin/bgs223

[35] Wang HW, Duan ZJ, Hu SS, Wang S. Expression of cAMP-dependent protein kinase inhibitor beta in colorectal carcinoma and its clinical significance]. Nan Fang Yi Ke Da Xue Xue Bao. 2017;37:744–9. 28669946 10.3969/j.issn.1673-4254.2017.06.05 PMC6744148

[36] Sung H, Ferlay J, Siegel RL, Laversanne M, Soerjomataram I, Jemal A, et al. Global Cancer Statistics 2020: GLOBOCAN Estimates of Incidence and Mortality Worldwide for 36 Cancers in 185 Countries. CA Cancer J Clin. 2021;71:209–49. 33538338 10.3322/caac.21660

[37] Prasad SM, Decastro GJ, Steinberg GD. Medscape. Urothelial carcinoma of the bladder: definition, treatment and future efforts. Nat Rev Urol. 2011;8:631–42. 21989305 10.1038/nrurol.2011.144

[38] Fonteyne V, Ost P, Bellmunt J, Droz JP, Mongiat-Artus P, Inman B, et al. Curative treatment for muscle invasive bladder cancer in elderly patients: a systematic review. Eur Urol. 2018;73:40–50. 28478043 10.1016/j.eururo.2017.03.019

[39] Patel VG, Oh WK, Galsky MD. Treatment of muscle-invasive and advanced bladder cancer in 2020. CA Cancer J Clin. 2020;70:404–23. 32767764 10.3322/caac.21631

[40] Zheng X, Ou Y, Shu M, Wang Y, Zhou Y, Su X, et al. Cholera toxin, a typical protein kinase A activator, induces G1 phase growth arrest in human bladder transitional cell carcinoma cells via inhibiting the c-Raf/MEK/ERK signaling pathway. Mol Med Rep. 2014;9:1773–9. 24626525 10.3892/mmr.2014.2054

[41] Ou Y, Zheng X, Gao Y, Shu M, Leng T, Li Y, et al. Activation of cyclic AMP/PKA pathway inhibits bladder cancer cell invasion by targeting MAP4-dependent microtubule dynamics. Urol Oncol. 2014;32:47.e21–8. 24140250 10.1016/j.urolonc.2013.06.017

[42] Kim WT, Seo SP, Byun YJ, Kang HW, Kim YJ, Lee SC, et al. Garlic extract in bladder cancer prevention: Evidence from T24 bladder cancer cell xenograft model, tissue microarray, and gene network analysis. Int J Oncol. 2017;51:204–12. 28498422 10.3892/ijo.2017.3993

[43] Morton DM, Tchao R. Regulation of motility and cytoskeletal organization of rat bladder-carcinoma cells by cyclic-Amp. Cell Motil Cytoskel. 1994;29:375–82. 10.1002/cm.970290410 7859299

[44] Liu C, Ke P, Zhang J, Zhang X, Chen X. Protein kinase inhibitor peptide as a tool to specifically inhibit protein kinase A. Front Physiol. 2020;11:574030. 33324237 10.3389/fphys.2020.574030 PMC7723848

[45] Kostenko S, Moens U. Heat shock protein 27 phosphorylation: kinases, phosphatases, functions and pathology. Cell Mol Life Sci. 2009;66:3289–307. 19593530 10.1007/s00018-009-0086-3 PMC11115724

[46] Katsogiannou M, Andrieu C, Rocchi P. Heat shock protein 27 phosphorylation state is associated with cancer progression. Front Genet. 2014;5:346. 25339975 10.3389/fgene.2014.00346 PMC4186339

[47] Moal IH, Jiménez-García B, Fernández-Recio J. CCharPPI web server: computational characterization of protein-protein interactions from structure. Bioinformatics. 2015;31:123–5. 25183488 10.1093/bioinformatics/btu594

[48] Zheng C, Lin Z, Zhao ZJ, Yang Y, Niu H, Shen X. MAPK-activated protein kinase-2 (MK2)-mediated formation and phosphorylation-regulated dissociation of the signal complex consisting of p38, MK2, Akt, and Hsp27. J Biol Chem. 2006;281:37215–26. 17015449 10.1074/jbc.M603622200

[49] Wang X, Chang X, He C, Fan Z, Yu Z, Yu B, et al. ATP5B promotes the metastasis and growth of gastric cancer by activating the FAK/AKT/MMP2 pathway. FASEB J. 2021;35:e20649. 33715234 10.1096/fj.202000608R

[50] Chen YT, Yang CC, Shao PL, Huang CR, Yip HK. Melatonin-mediated downregulation of ZNF746 suppresses bladder tumorigenesis mainly through inhibiting the AKT-MMP-9 signaling pathway. J Pineal Res. 2019;66:e12536. 30372570 10.1111/jpi.12536

[51] Coen JJ, Zhang P, Saylor PJ, Lee CT, Wu CL, Parker W, et al. Bladder Preservation With Twice-a-Day Radiation Plus Fluorouracil/Cisplatin or Once Daily Radiation Plus Gemcitabine for Muscle-Invasive Bladder Cancer: NRG/RTOG 0712-A Randomized Phase II Trial. J Clin Oncol. 2019;37:44–51. 30433852 10.1200/JCO.18.00537 PMC6354769

[52] Kelley RK, Ueno M, Yoo C, Finn RS, Furuse J, Ren Z, et al. Pembrolizumab in combination with gemcitabine and cisplatin compared with gemcitabine and cisplatin alone for patients with advanced biliary tract cancer (KEYNOTE-966): a randomised, double-blind, placebo-controlled, phase 3 trial. Lancet. 2023;401:1853–65. 37075781 10.1016/S0140-6736(23)00727-4

[53] Liu J, Jing W, Wang T, Hu Z, Lu H. Functional metabolomics revealed the dual-activation of cAMP-AMP axis is a novel therapeutic target of pancreatic cancer. Pharm Res. 2023;187:106554. 10.1016/j.phrs.2022.106554 36379357

[54] Okuno M, Adachi S, Kozawa O, Shimizu M, Yasuda I. The clinical significance of phosphorylated heat shock protein 27 (HSPB1) in pancreatic cancer. Int J Mol Sci. 2016;17:137. 10.3390/ijms17010137 PMC4730376 26805817

[55] Wiley JC, Wailes LA, Idzerda RL, McKnight GS. Role of regulatory subunits and protein kinase inhibitor (PKI) in determining nuclear localization and activity of the catalytic subunit of protein kinase A. J Biol Chem. 1999;274:6381–7. 10037729 10.1074/jbc.274.10.6381

[56] Doppler H, Storz P, Li J, Comb MJ, Toker A. A phosphorylation state-specific antibody recognizes Hsp27, a novel substrate of protein kinase D. J Biol Chem. 2005;280:15013–9. 15728188 10.1074/jbc.C400575200

[57] Gaestel M, Schroder W, Benndorf R, Lippmann C, Buchner K, Hucho F, et al. Identification of the phosphorylation sites of the murine small heat shock protein hsp25. J Biol Chem. 1991;266:14721–4. 1860870

[58] Cairns J, Qin S, Philp R, Tan YH, Guy GR. Dephosphorylation of the small heat shock protein Hsp27 in vivo by protein phosphatase 2A. J Biol Chem. 1994;269:9176–83. 7510704

[59] Butt E, Immler D, Meyer HE, Kotlyarov A, Laass K, Gaestel M. Heat shock protein 27 is a substrate of cGMP-dependent protein kinase in intact human platelets: phosphorylation-induced actin polymerization caused by HSP27 mutants. J Biol Chem. 2001;276:7108–13. 11383510 10.1074/jbc.m009234200

[60] Huang SY, Tsai ML, Chen GY, Wu CJ, Chen SH. A systematic MS-based approach for identifying in vitro substrates of PKA and PKG in rat uteri. J Proteome Res. 2007;6:2674–84. 17564427 10.1021/pr070134c

[61] Zhou M, Lambert H, Landry J. Transient activation of a distinct serine protein kinase is responsible for 27-kDa heat shock protein phosphorylation in mitogen-stimulated and heat-shocked cells. J Biol Chem. 1993;268:35–43. 8380159

[62] Stokoe D, Engel K, Campbell DG, Cohen P, Gaestel M. Identification of MAPKAP kinase 2 as a major enzyme responsible for the phosphorylation of the small mammalian heat shock proteins. FEBS Lett. 1992;313:307–13. 1332886 10.1016/0014-5793(92)81216-9

[63] Saklatvala J, Guesdon F. Interleukin 1 signal transduction. Agents Actions Suppl. 1991;35:35–40. 1838232

[64] Nakajima K, Hirade K, Ishisaki A, Matsuno H, Suga H, Kanno Y, et al. Akt regulates thrombin-induced HSP27 phosphorylation in aortic smooth muscle cells: function at a point downstream from p38 MAP kinase. Life Sci. 2005;77:96–107. 15848222 10.1016/j.lfs.2004.12.017

[65] Al-Madhoun AS, Chen YX, Haidari L, Rayner K, Gerthoffer W, McBride H, et al. The interaction and cellular localization of HSP27 and ERβ are modulated by 17β-estradiol and HSP27 phosphorylation. Mol Cell Endocrinol. 2007;270:33–42. 17350752 10.1016/j.mce.2007.02.002

[66] Singh MK, Sharma B, Tiwari PK. The small heat shock protein Hsp27: Present understanding and future prospects. J Therm Biol. 2017;69:149–54. 29037376 10.1016/j.jtherbio.2017.06.004

[67] Lucijanic M, Livun A, Tupek KM, Stoos-Veic T, Aralica G, Gecek I, et al. Heat shock protein 27 (HSP27/HSPB1) expression is increased in patients with primary and secondary myelofibrosis and may be affecting their survival. Leuk Lymphoma. 2017;58:2497–500. 28278711 10.1080/10428194.2017.1296146

[68] Ischia J, So AI. The role of heat shock proteins in bladder cancer. Nat Rev Urol. 2013;10:386–95. 23670183 10.1038/nrurol.2013.108

[69] Chipumuro E, Marco E, Christensen CL, Kwiatkowski N, Zhang T, Hatheway CM, et al. CDK7 inhibition suppresses super-enhancer-linked oncogenic transcription in MYCN-driven cancer. Cell. 2014;159:1126–39. 25416950 10.1016/j.cell.2014.10.024 PMC4243043

[70] Wang H, Hong B, Li X, Deng K, Li H, Yan Lui VW, et al. JQ1 synergizes with the Bcl-2 inhibitor ABT-263 against MYCN-amplified small cell lung cancer. Oncotarget. 2017;8:86312–24. 29156797 10.18632/oncotarget.21146 PMC5689687

[71] Qin XY, Suzuki H, Honda M, Okada H, Kaneko S, Inoue I, et al. Prevention of hepatocellular carcinoma by targeting MYCN-positive liver cancer stem cells with acyclic retinoid. Proc Natl Acad Sci USA. 2018;115:4969–74. 29686061 10.1073/pnas.1802279115 PMC5949003

[72] Hossain S, Takatori A, Nakamura Y, Suenaga Y, Kamijo T, Nakagawara A. NLRR1 enhances EGF-mediated MYCN induction in neuroblastoma and accelerates tumor growth in vivo. Cancer Res. 2012;72:4587–96. 22815527 10.1158/0008-5472.CAN-12-0943

[73] Zhang W, Liu B, Wu W, Li L, Broom BM, Basourakos SP, et al. Targeting the MYCN-PARP-DNA damage response pathway in neuroendocrine prostate cancer. Clin Cancer Res. 2018;24:696–707. 29138344 10.1158/1078-0432.CCR-17-1872 PMC5823274

[74] Tong Q, Ouyang S, Chen R, Huang J, Guo L. MYCN-mediated regulation of the HES1 promoter enhances the chemoresistance of small-cell lung cancer by modulating apoptosis. Am J Cancer Res. 2019;9:1938–56. 31598396 PMC6780666

[75] Wu S, Xu H, Zhang R, Wang X, Yang J, Li X, et al. Circular RNA circLAMA3 inhibits the proliferation of bladder cancer by directly binding an mRNA. Mol Ther Oncolytics. 2022;24:742–54. 35317525 10.1016/j.omto.2022.02.020 PMC8908064

[76] Nakashima M, Adachi S, Yasuda I, Yamauchi T, Kawaguchi J, Itani M, et al. Phosphorylation status of heat shock protein 27 plays a key role in gemcitabine-induced apoptosis of pancreatic cancer cells. Cancer Lett. 2011;313:218–25. 21999932 10.1016/j.canlet.2011.09.008

[77] Matsui Y, Hadaschik BA, Fazli L, Andersen RJ, Gleave ME, So AI. Intravesical combination treatment with antisense oligonucleotides targeting heat shock protein-27 and HTI-286 as a novel strategy for high-grade bladder cancer. Mol Cancer Ther. 2009;8:2402–11. 19625496 10.1158/1535-7163.MCT-09-0148

[78] Abdel-Rahman O, Elsayed Z, Elhalawani H. Gemcitabine-based chemotherapy for advanced biliary tract carcinomas. Cochrane Db Syst Rev. 2018;4:CD011746. 10.1002/14651858.CD011746.pub2 PMC6494548 29624208

[79] Cathomas R, Lorch A, Bruins HM, Compérat EM, Cowan NC, Efstathiou JA, et al. The 2021 Updated European association of urology guidelines on metastatic urothelial carcinoma. Eur Urol. 2022;81:95–103. 34742583 10.1016/j.eururo.2021.09.026

[80] Lindgren D, Frigyesi A, Gudjonsson S, Sjödahl G, Hallden C, Chebil G, et al. Combined gene expression and genomic profiling define two intrinsic molecular subtypes of urothelial carcinoma and gene signatures for molecular grading and outcome. Cancer Res. 2010;70:3463–72. 20406976 10.1158/0008-5472.CAN-09-4213

[81] Sjödahl G, Lauss M, Lövgren K, Chebil G, Gudjonsson S, Veerla S, et al. A molecular taxonomy for urothelial carcinoma. Clin Cancer Res. 2012;18:3377–86. 22553347 10.1158/1078-0432.CCR-12-0077-T

[82] Cancer Genome Atlas Research N. Comprehensive molecular characterization of urothelial bladder carcinoma. Nature. 2014;507:315–22. 24476821 10.1038/nature12965 PMC3962515

[83] Choi W, Porten S, Kim S, Willis D, Plimack ER, Hoffman-Censits J, et al. Identification of distinct basal and luminal subtypes of muscle-invasive bladder cancer with different sensitivities to frontline chemotherapy. Cancer Cell. 2014;25:152–65. 24525232 10.1016/j.ccr.2014.01.009 PMC4011497

[84] Robertson AG, Kim J, Al-Ahmadie H, Bellmunt J, Guo GW, Cherniack AD, et al. Comprehensive molecular characterization of muscle-invasive bladder cancer. Cell. 2017;171:540. 28988769 10.1016/j.cell.2017.09.007 PMC5687509

[85] Sjödahl G, Eriksson P, Liedberg F, Höglund M. Molecular classification of urothelial carcinoma: global mRNA classification versus tumour-cell phenotype classification. J Pathol. 2017;242:113–25. 28195647 10.1002/path.4886 PMC5413843

[86] Guo CC, Bondaruk J, Yao H, Wang ZQ, Zhang L, Lee S, et al. Assessment of luminal and basal phenotypes in bladder cancer. Sci Rep. 2020;10:9743. 10.1038/s41598-020-66747-7 PMC7298008 32546765

[87] Guo CC, Lee SKY, Lee JG, Chen HQ, Zaleski M, Choi W, et al. Molecular profile of bladder cancer progression to clinically aggressive subtypes. Nat Rev Urol. 2024;21:391-405. 10.1038/s41585-023-00847-7 38321289

[88] Seiler R, Ashab HA, Erho N, van Rhijn BWG, Winters B, Douglas J, et al. Impact of molecular subtypes in muscle-invasive bladder cancer on predicting response and survival after neoadjuvant chemotherapy. Eur Urol. 2017;72:544–54. 28390739 10.1016/j.eururo.2017.03.030

[89] Seiler R, Gibb EA, Wang NQ, Oo HZ, Lam HM, van Kessel KE, et al. Divergent biological response to neoadjuvant chemotherapy in muscle-invasive bladder cancer. Clin Cancer Res. 2019;25:5082–93. 30224344 10.1158/1078-0432.CCR-18-1106

[90] Warrick JI, Walter V, Yamashita H, Chung E, Shuman L, Amponsa VO, et al. FOXA1, GATA3 and PPARγ cooperate to drive luminal subtype in bladder cancer: a molecular analysis of established human cell lines. Sci Rep. 2016;6:38531. 10.1038/srep38531 PMC5141480 27924948

[91] Cheng HC, Kemp BE, Pearson RB, Smith AJ, Misconi L, Vanpatten SM, et al. A potent synthetic peptide inhibitor of the camp-dependent protein-kinase. J Biol Chem. 1986;261:989–92. 3511044

[92] Glass DB, Cheng HC, Kemp BE, Walsh DA. Differential and common recognition of the catalytic sites of the Cgmp-dependent and camp-dependent protein-kinases by inhibitory peptides derived from the heat-stable inhibitor protein. J Biol Chem. 1986;261:2166–71. 3017964

[93] Day RN, Walder JA, Maurer RAA. Protein-kinase inhibitor gene reduces both basal and multihormone-stimulated prolactin gene-transcription. J Biol Chem. 1989;264:431–6. 2535842

[94] Smith MK, Colbran RJ, Soderling TR. Specificities of autoinhibitory domain peptides for 4 protein-kinases - implications for intact cell studies of protein-kinase function. J Biol Chem. 1990;265:1837–40. 2153665

[95] Fei L, Wang Y. microRNA-495 reduces visceral sensitivity in mice with diarrhea-predominant irritable bowel syndrome through suppression of the PI3K/AKT signaling pathway via PKIB. IUBMB Life. 2020;72:1468–80. 32187820 10.1002/iub.2270

[96] Martí-Renom MA, Stuart AC, Fiser A, Sánchez R, Melo F, Sali A. Comparative protein structure modeling of genes and genomes. Annu Rev Bioph Biom. 2000;29:291–325. 10.1146/annurev.biophys.29.1.291 10940251

[97] Laskowski RA, Macarthur MW, Moss DS, Thornton JM. Procheck - a program to check the stereochemical quality of protein structures. J Appl Crystallogr. 1993;26:283–91.

[98] Chen R, Li L, Weng ZP. ZDOCK: An initial-stage protein-docking algorithm. Proteins. 2003;52:80–7. 12784371 10.1002/prot.10389

[99] Pierce BG, Hourai Y, Weng ZP. Accelerating protein docking in ZDOCK using an advanced 3D convolution library. Plos One. 2011;6:e24657. 10.1371/journal.pone.0024657 PMC3176283 21949741

[100] Wiehe K, Pierce B, Mintseris J, Tong WW, Anderson R, Chen R, et al. ZDOCK and RDOCK performance in CAPRI rounds 3, 4, and 5. Proteins. 2005;60:207–13. 15981263 10.1002/prot.20559

[101] Vreven T, Pierce BG, Hwang H, Weng ZP. Performance of ZDOCK in CAPRI Rounds 20-26. Proteins. 2013;81:2175–82. 24123140 10.1002/prot.24432 PMC3975700

[102] Essmann U, Perera L, Berkowitz ML, Darden T, Lee H, Pedersen LG. A smooth particle Mesh Ewald Method. J Chem Phys. 1995;103:8577–93.

[103] Hess B, Bekker H, Berendsen HJC, Fraaije JGEM. LINCS: A linear constraint solver for molecular simulations. J Comput Chem. 1997;18:1463–72.

[104] Bussi G, Donadio D, Parrinello M. Canonical sampling through velocity rescaling. J Chem Phys. 2007;126:014101. 10.1063/1.2408420 17212484

[105] Van der Spoel D, Lindahl E, Hess B, Groenhof G, Mark AE, Berendsen HJC. GROMACS: Fast, flexible, and free. J Comput Chem. 2005;26:1701–18. 16211538 10.1002/jcc.20291

[106] Lindorff-Larsen K, Piana S, Palmo K, Maragakis P, Klepeis JL, Dror RO, et al. Improved side-chain torsion potentials for the Amber ff99SB protein force field. Proteins. 2010;78:1950–8. 20408171 10.1002/prot.22711 PMC2970904

