# Supplementary material for: PKIB facilitates bladder cancer proliferation and metastasis through mediation of HSP27 phosphorylation by PKA
Source: Cell Death Dis. 2025 Jul 1;16(1):470. doi: 10.1038/s41419-025-07814-7 (PMC12219054; doi:10.1038/s41419-025-07814-7)
Supplement: Supplementary file 1 — Supplementary materials [file 41419_2025_7814_MOESM1_ESM.docx]

**Supplementary Figures and Figure Legends**


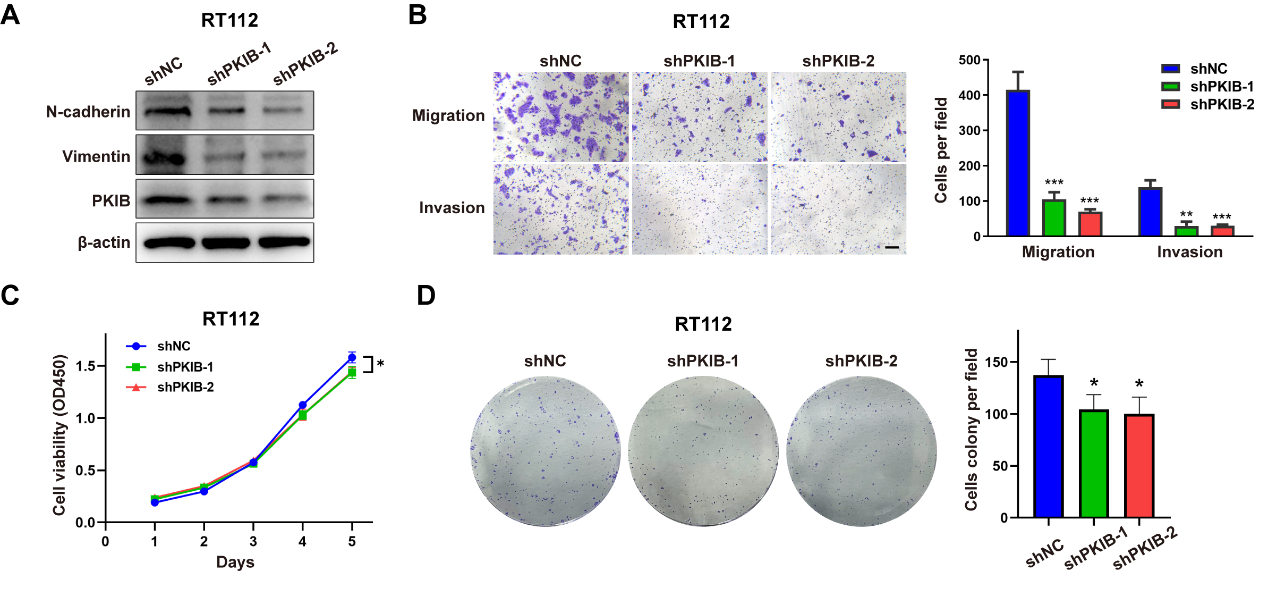


**Figure S1. Knockdown of *PKIB* inhibits the proliferation, EMT, migration and invasion of RT112 cells.**

1. The expression of the EMT markers N-cadherin and Vimentin was analysed via Western blotting. β-actin served as an internal control.
2. The migratory and invasive capabilities of RT112 cells with *PKIB* knockdown were evaluated via Transwell assays. The data are shown as the mean ± SD (n = 3). Migration shPKIB-1, *** P=0.0006; shPKIB-2, *** P=0.0003; Invasion shPKIB-1, ** P=0.0012; shPKIB-2, *** P=0.0006 by unpaired Student’s t test. Scale bar, 100 μm.
3. Relative growth of indicated RT112 cells was determined by the CCK-8 assay. The data are shown as the means ± SD (n=3). shPKIB-1 * P=0.0321; shPKIB-2 * P=0.0228 by unpaired Student’s t test.
4. Representative images of colony formation assay of the indicated RT112 cells. The data are shown as the means ± SD (n=3). shPKIB-1 * P=0.0151; shPKIB-2 ** P=0.0033 by unpaired Student’s t test.


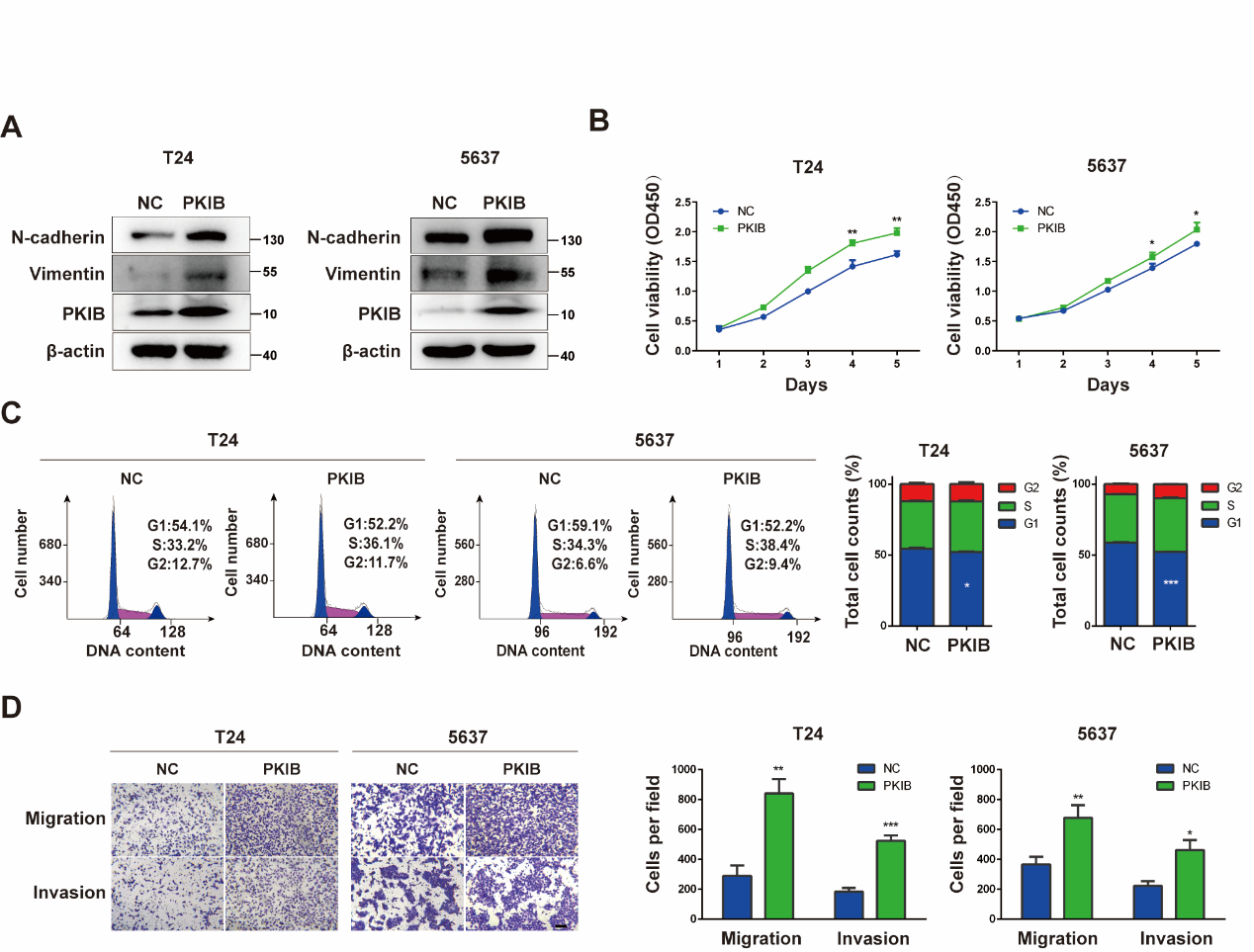


**Figure S2. *PKIB* overexpression promotes BLCA cell proliferation, migration and invasion *in vitro*.**

1. Western blot analysis of PKIB and EMT markers (N-cadherin and Vimentin) protein levels in T24 and 5637 cells stably transfected with a negative control vector (NC) or *PKIB* overexpression vector (PKIB).
2. Relative growth of T24 and 5637 cells overexpressing *PKIB* or empty vector as indicated. The data are shown as the means ± SD (n=3). T24 4 days, ** P = 0.0046; 5 days, ** P = 0.0029; 5637 4 days, * P =0.0435; 5 days, * P = 0.0321 by unpaired Student’s t test.
3. The cell cycle distribution was detected by PI staining in *PKIB*-overexpressing and control T24 and 5637 cells. The data are shown as the means ± SD (n=3). T24 cell, * P =0.0114; 5637 cell *** P < 0.0001 by unpaired Student’s test.
4. Transwell assays were performed to assess the ability of migration and invasion of *PKIB*-overexpressing T24 and 5637 cells. Migrated and invasive cells were stained and counted in at least three microscopic fields. The data are shown as the means ± SD (n=3). T24 migration ** P =0.0013; T24 invasion *** P= 0.0002; 5637 migration ** P = 0.0050; 5637 invasion * P =0.0129 by unpaired Student’s t test. Scale bar, 100 μm.


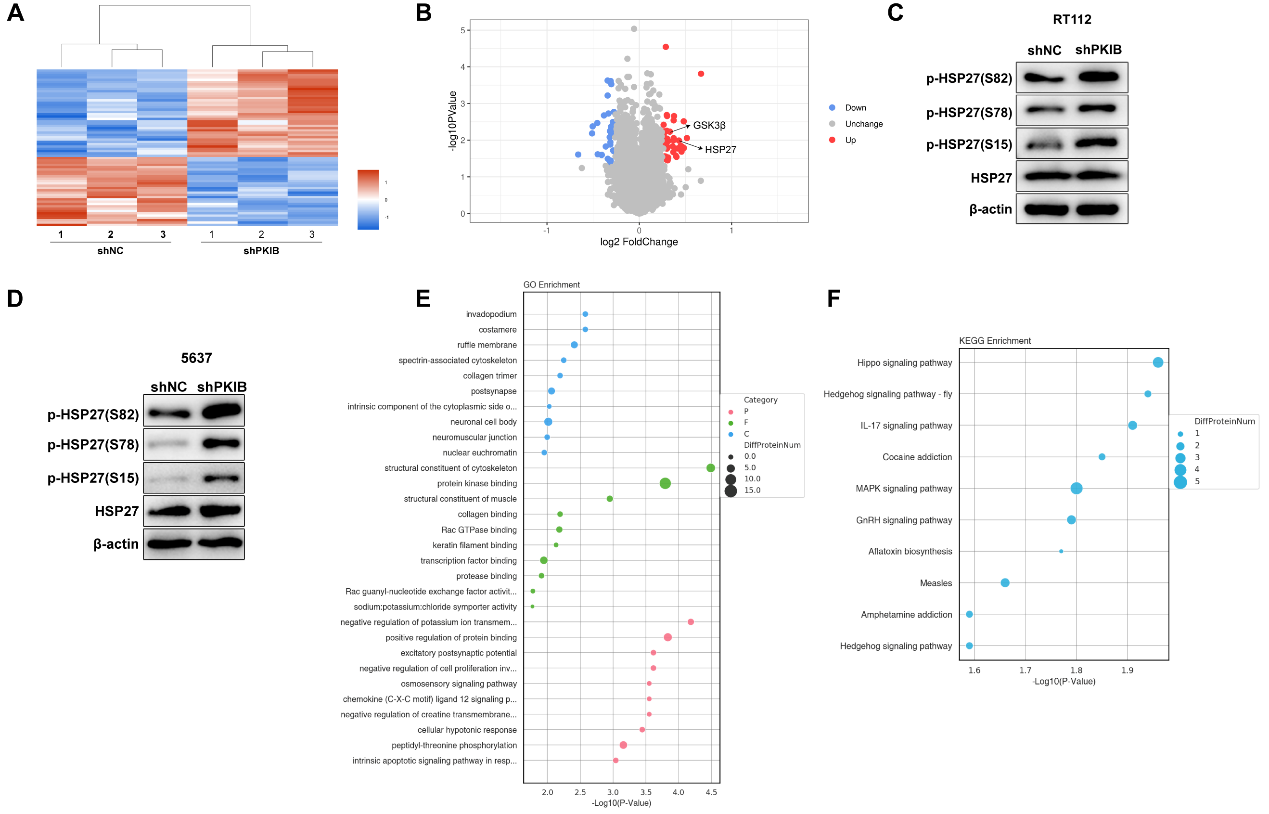
**Figure S****3. Phosphoproteomic analysis of *PKIB*-knockdown and control T24 cells.**

1. Hierarchical cluster analysis (heatmap) was used to visualize phosphopeptides with p values less than 0.05. Three independent samples were analysed in each group. Each column and row indicate a test sample and a phosphorylated peptide, respectively. Red and blue indicate up- and downregulated phosphorylation, respectively.
2. A volcano plot was generated to show phosphopeptides with a fold change >1.2 or <0.8 and a p value of less than 0.05. The red dots indicate phosphopeptides with a fold change >1.2, including GSK3β and HSP27. The blue dots indicate phosphopeptides with a fold change < 0.8.
3. Western blot analysis of the phosphorylation level of HSP27 in RT112 cells with *PKIB* knockdown.
4. Western blot analysis of the phosphorylation level of HSP27 in 5637 cells with *PKIB* knockdown.
5. GO enrichment analysis showing the top 10 significant biological processes (P), molecular functions (F) and cellular components (C).
6. KEGG analysis showing the top 10 pathways.


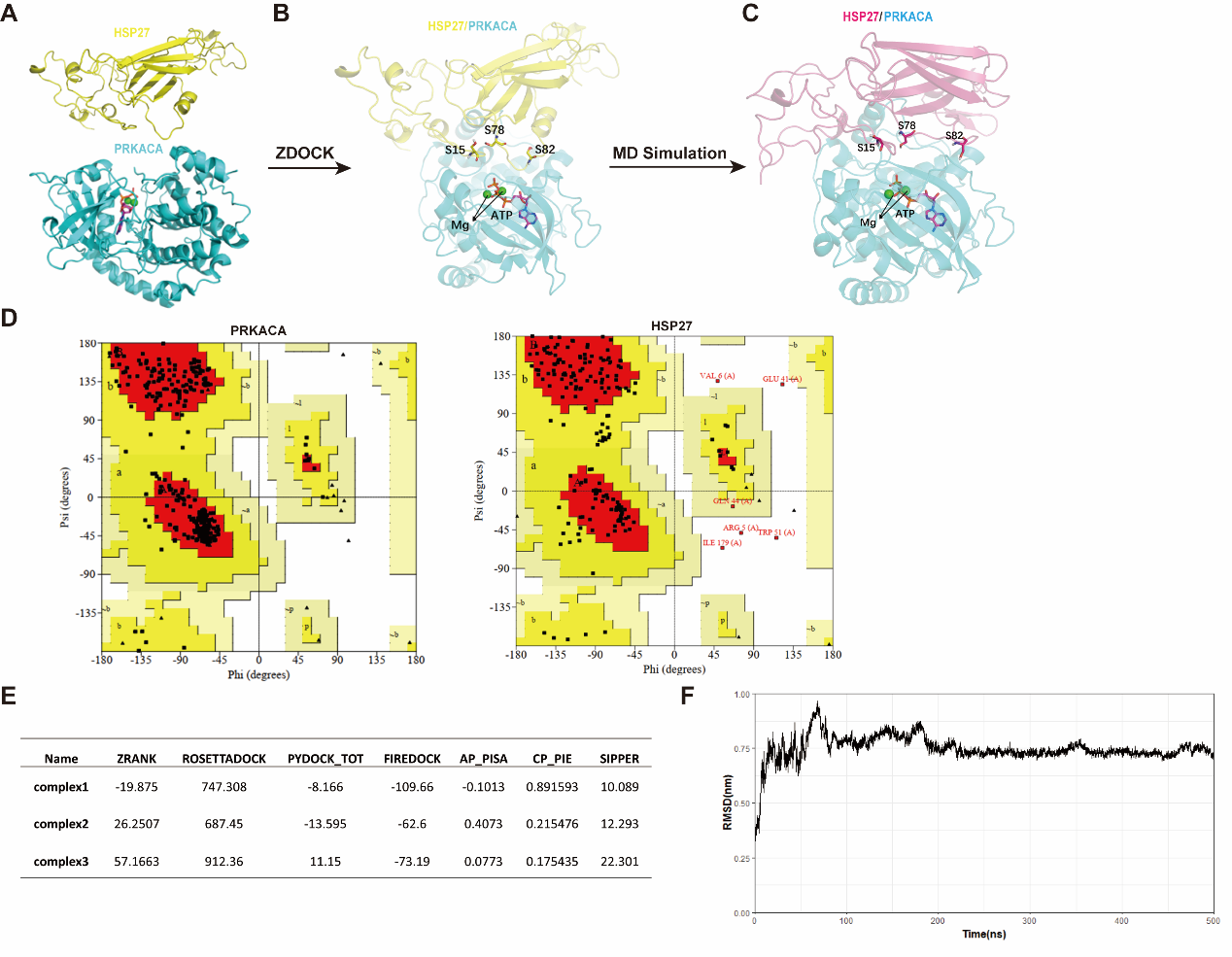


**Figure S4. Structural annotation of the HSP27/PRKACA complex structure.**

1. The modelled structures of HSP27 (upper panel, yellow cartoon) and PRKACA (lower panel, cyan cartoon).
2. The HSP27/PRKACA complex structure constructed by ZDOCK.
3. The ending conformation of the complex structure after 500 ns of MD simulation and comparisons of the initial (yellow) and ending (warmpink) conformations of the phosphorylation active site and the three serine residues from the 500 ns MD simulation are highlighted.
4. Ramachandran plot of the PRKACA (left) and HSP27 (right) structures.
5. The weighted binding free energy of the three complexes given by the seven scoring functions in CCharPPI. The lower the value is, the tighter the binding affinity of the complex.
6. RMS deviation for the α-carbon atoms of the HSP27/PRKACA complex during the MD simulation.


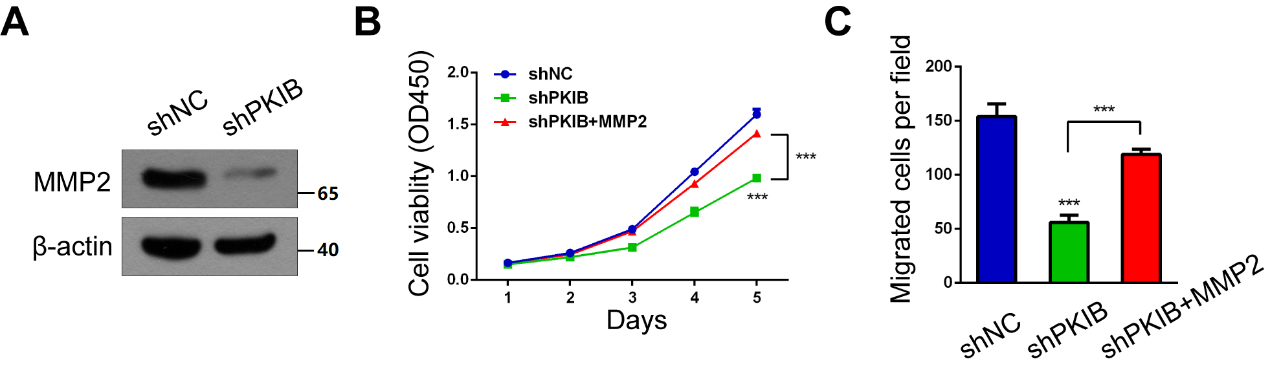


**Figure S5. *MMP2* overexpression partially rescues the PKIB-mediated inhibition of proliferation and migration in T24 cells.**

1. Western blot analysis of MMP2 expression in *PKIB*-knockdown T24 cells (shPKIB) or negative control cells (shNC).
2. PKIB-knockdown T24 cells were transfected with *MMP2*, and a CCK8 assay was performed to assess their proliferative ability. The data are shown as the mean ± SD (n=3). *** P < 0.0001 by unpaired Student’s t test.
3. Migratory capacity of the above treated T24 cells. The data are shown as the mean ± SD (n=3). shPKIB *** P = 0.0009; shPKIB+MMP2 *** P = 0.0002 by unpaired Student’s t test.


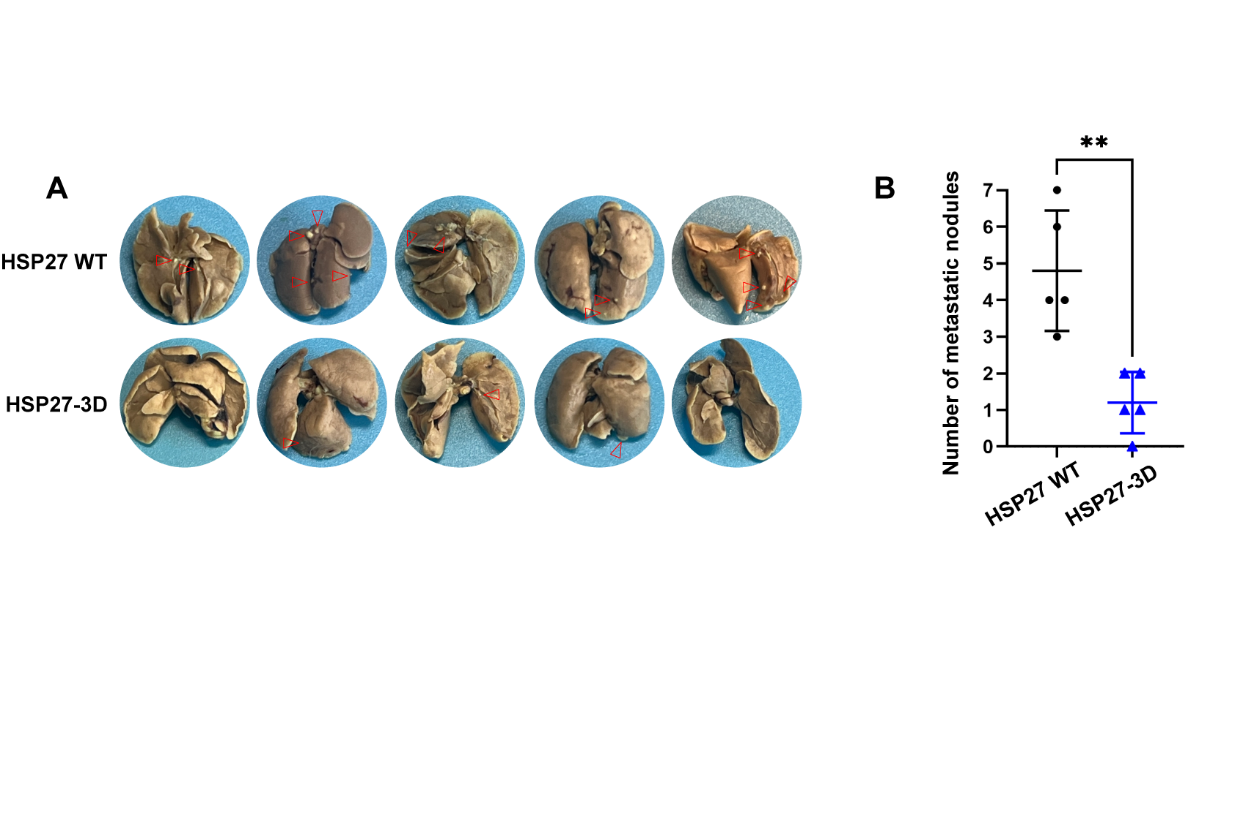


**Figure S6. HSP27 phosphorylation suppresses BLCA cell metastasis *in vivo*.**

1. Photographs of metastatic nodules established in mice after injection of indicated T24 cells for 8 weeks. Red arrowheads indicate metastatic nodules formed in lungs.
2. Plots showing the difference in lung metastatic nodules between the HSP27 WT group and HSP27-3D group (5 mice per group). ^**^ P =0.0048 by unpaired Student’s t test.


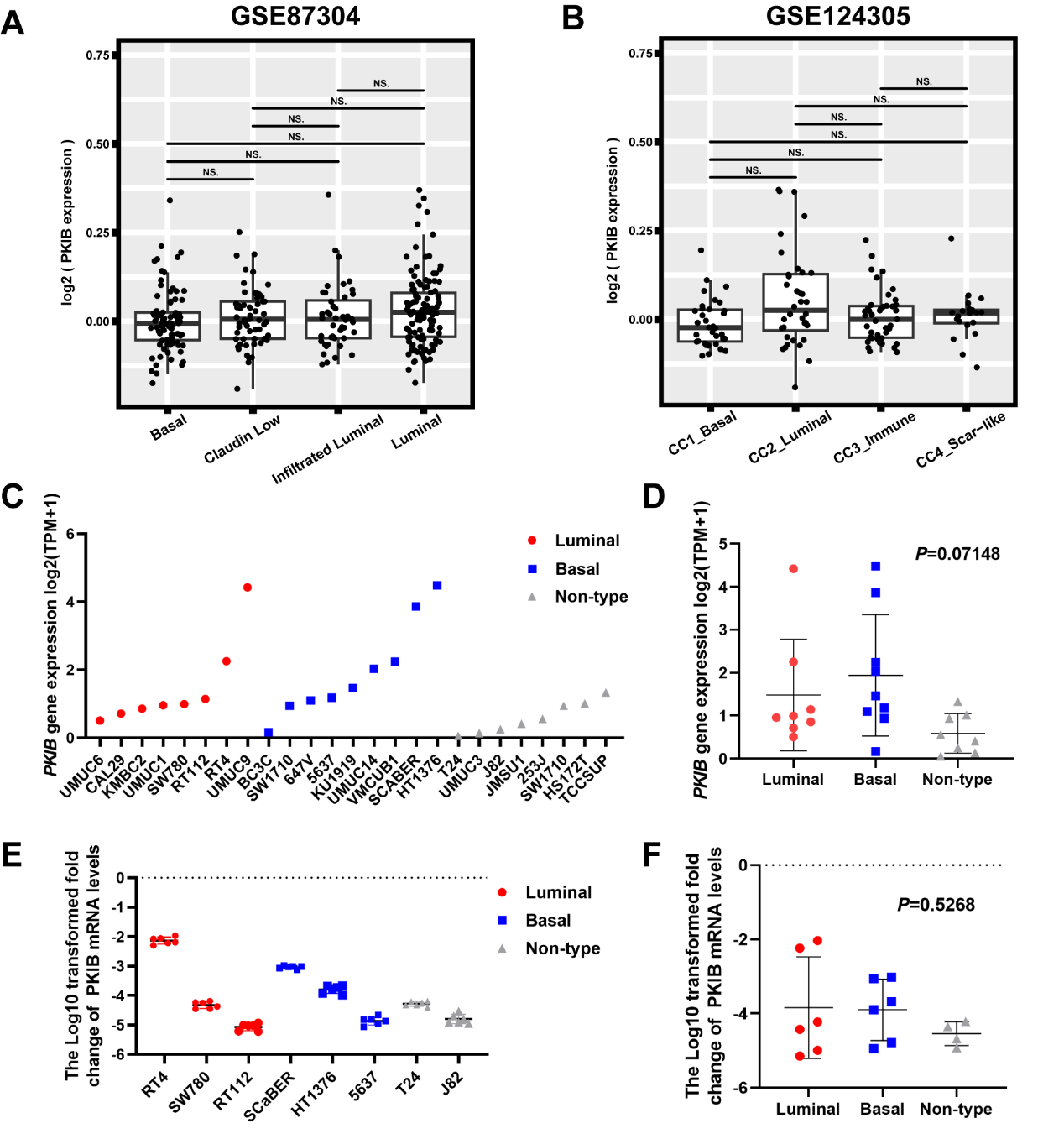


**Figure S7. Expression of *PKIB* in BLCA of different molecular subtypes.**

A. *PKIB* mRNA expression in BLCA using the GSE87304 dataset.

B. *PKIB* mRNA expression in BLCA using the GSE124305 dataset.

C. *PKIB* mRNA expression for individual bladder cancer cell lines are shown. Red denotes luminal cell lines, blue denotes basal cell lines and gray denotes non-type cell lines (Source: DepMap database).

D. *PKIB* mRNA expression of above individual bladder cancer cell lines. Statistical analysis was performed using one-way ANOVA. P = 0.07148.

E. qRT‒PCR analysis of *PKIB* mRNA expression in RT4, SW780, RT112, SCaBER, HT1376, 5637, T24 and J82 cell lines. Red denotes luminal cell lines, blue denotes basal cell lines and gray denotes non-type cell lines. Data are shown as the mean ± SD (n=6).

F. *PKIB* mRNA expression of the above 8 individual bladder cancer cell lines. Statistical analysis was performed using one-way ANOVA. P = 0.5268. Data are shown as the mean ± SD (n=2).

**Supplementary Tables S1-S4, S6**

**Table S1. Primers used for plasmid construction, qRT‒PCR, and ChIP, as well as sequences of siRNAs.**

| Primers for plasmid construction Sequence, 5' – 3' | |
| --- | --- |
| FLAG-PKA-F | AGACCCAAGCTGGCTAGCATGGGCAACGCCGCC |
| FLAG-PKA-R | AACATCGTATGGGTAACCGGTAAACTCAGAAAACTCCTTGCCACAC |
| HA-HSP27-F | AGACCCAAGCTGGCTAGCATGACCGAGCGCCGCGTC |
| HA-HSP27-R  HA-NTD-F  HA-NTD-R HA-△NTD-F  HA-△NTD-R  HA-ACD-F  HA-ACD-R | AACATCGTATGGGTAACCGGTCTTGGCGGCAGTCTCATCG  AGACCCAAGCTGGCTAGCATGACCGAGCGCCGCGTC  AACATCGTATGGGTAACCGGTGACCCCGCTGCTGAGTTGCC  AGACCCAAGCTGGCTAGCTCGGAGATCCGGCACACTGCG  AACATCGTATGGGTAACCGGTCTTGGCGGCAGTCTCATCG  AGACCCAAGCTGGCTAGCTCGGAGATCCGGCACACTGCG  AACATCGTATGGGTAACCGGTGGACTGCGTGGCTAGCTTGGGC |

| Primers for qRT‒PCR Sequence, 5' – 3' |
| --- |
| PKIA-F GCCTTGAAATTAGCAGGTCTTGA  PKIA-R GCTTCCCCACTTTGTTCTGTAG  PKIB-F GAGTCTGGGGTCGCCAATTTT  PKIB-R TGAACTCTGGATGTCTGGTAAGG  PKIG-F GTCCTCCTACTCGGACTTCAT  PKIG-R CTTCCCTCCACCTGTCCTTCT  MYCN-F ACCCGGACGAAGATGACTTCT  MYCN-R CAGCTCGTTCTCAAGCAGCAT  β-actin-F CATGTACGTTGCTATCCAGGC  β-actin-R CTCCTTAATGTCACGCACGAT |

| Primers for ChIP Sequence, 5' – 3' |
| --- |
| MYCN 1-F ACGCGGGTTGTGTGACGATCAC  MYCN 1-R GTCCCGCCCCCAGGCTACGAG  MYCN 2-F TTGGGCGACCCGATCCATC  MYCN 2-R AAAGACTTGGCATTGCTGG |

| siRNAs Sequence, 5' – 3' |
| --- |
| siMYCN-1 CCUCGAGUUUGACUCGCUATT  siMYCN-2 UCUUUAGCAACUGCUGCUGTT  siPKIB-1 TTGCCCUAAGCAGCAUGUAUA  siPKIB-2 TTUACACAUGCUGCUUAGGGC  siNC UUCUCCGAACGUGUCACGUTT |

| shRNAs Sequence, 5' – 3' |
| --- |
| shPKIB-1 CCGGCCACAGACGGAACCTCAGATTCTCGAGAATCTGAGGTTCCGTCTGTGGTTTTTG  shPKIB-2  CCGGCACAGACGGAACCTCAGATTTCTCGAGAAATCTGAGGTTCCGTCTGTGTTTTTG  shNC  CCGGTTCTCCGAACGTGTCACGTTTCTCGAGAAACGTGACACGTTCGGAGAATTTTTG |

**Table S2. Demographic and clinical characteristics of 40 BLCA patients and relative mRNA expression of *PKIA, PKIB* and *PKIG* in 40 paired BLCA tissues.**

| Case | Age | Gender | Tissue  source | TNM | Depth of invasion | PKIA  (T/N) | PKIB  (T/N) | PKIG  (T/N) |
| --- | --- | --- | --- | --- | --- | --- | --- | --- |
| \| 1 \| \| --- \| \| 2 \| \| 3 \| \| 4 \| \| 5 \| \| 6 \| \| 7 \| \| 8 \| \| 9 \| \| 10 \| \| 11 \| \| 12 \| \| 13 \| \| 14 \| \| 15 \| \| 16 \| \| 17 \| \| 18 \| \| 19 \| \| 20 \| \| 21 \| \| 22 \| \| 23 \| \| 24 \| \| 25 \| \| 26 \| \| 27 \| \| 28 \| \| 29 \| \| 30 \| \| 31 \| \| 32 \| \| 33 \| \| 34 \| \| 35 \| \| 36 \| \| 37 \| \| 38 \| \| 39 \| \| 40 \| | \| 62 \| \| --- \| \| 79 \| \| 76 \| \| 86 \| \| 56 \| \| 74 \| \| 70 \| \| 55 \| \| 63 \| \| 59 \| \| 82 \| \| 69 \| \| 79 \| \| 69 \| \| 35 \| \| 82 \| \| 68 \| \| 77 \| \| 57 \| \| 90 \| \| 74 \| \| 55 \| \| 81 \| \| 84 \| \| 86 \| \| 43 \| \| 53 \| \| 55 \| \| 57 \| \| 78 \| \| 76 \| \| 76 \| \| 65 \| \| 66 \| \| 81 \| \| 55 \| \| 46 \| \| 73 \| \| 79 \| \| 62 \| | \| male \| \| --- \| \| male \| \| female \| \| male \| \| male \| \| male \| \| male \| \| male \| \| male \| \| male \| \| male \| \| male \| \| female \| \| male \| \| male \| \| female \| \| male \| \| male \| \| male \| \| female \| \| female \| \| male \| \| male \| \| male \| \| female \| \| male \| \| male \| \| male \| \| male \| \| male \| \| female \| \| female \| \| male \| \| male \| \| female \| \| male \| \| male \| \| male \| \| male \| \| female \| | TURBT  TURBT  TURBT  TURBT  TURBT  TURBT  TURBT  TURBT  TURBT  TURBT  TURBT  TURBT  TURBT  TURBT  TURBT  TURBT  TURBT  TURBT  TURBT  TURBT TURBT  TURBT  TURBT  TURBT  TURBT  TURBT  TURBT  TURBT  TURBT  TURBT  TURBT  TURBT  TURBT  TURBT  TURBT  TURBT  TURBT  TURBT  TURBT  TURBT | \| TaN0M0 \| \| --- \| \| TaN0M0 \| \| T1N0M0 \| \| T1N2M0 \| \| T1N0M0 \| \| T2N0M0 \| \| T1N0M0 \| \| TaN0M1 \| \| T1N0M0 \| \| T1N0M0 \| \| T1N0M0 \| \| T1N0M0 \| \| T1N0M0 \| \| T1N0M1 \| \| T1N0M0 \| \| TaN0M0 \| \| T1N0M0 \| \| T1N0M0 \| \| T2N0M0 \| \| TaN0M0 \| \| T1N0M0 \| \| T2N0M0 \| \| T1N0M0 \| \| TaN0M0 \| \| TaN0M0 \| \| T1N0M0 \| \| T1N0M0 \| \| TaN0M0 \| \| T1N0M0 \| \| T1N0M0 \| \| T1N0M0  TaN0M0  T1N0M0  T1N0M0  T1N0M0  T1N0M0  T1N0M0  TaN0M0  T1N0M0  T1N0M0 \| | \| NMIBC \| \| --- \| \| NMIBC \| \| NMIBC \| \| MIBC \| \| NMIBC \| \| NMIBC \| \| NMIBC \| \| NMIBC \| \| NMIBC \| \| MIBC \| \| MIBC \| \| NMIBC \| \| NMIBC \| \| NMIBC \| \| NMIBC \| \| NMIBC \| \| NMIBC \| \| MIBC \| \| MIBC \| \| NMIBC \| \| NMIBC \| \| MIBC \| \| NMIBC \| \| MIBC \| \| NMIBC \| \| NMIBC \| \| MIBC \| \| NMIBC \| \| NMIBC \| \| MIBC \| \| NMIBC  NMIBC  NMIBC  NMIBC  NMIBC  NMIBC  NMIBC  NMIBC  NMIBC  NMIBC \| | \| 0.22596 \| \| --- \| \| 0.00167 \| \| 0.04775 \| \| 0.38113 \| \| 6.20364 \| \| 2.32468 \| \| 2.92032 \| \| 6.41410 \| \| 0.21697 \| \| 0.22915 \| \| 0.78613 \| \| 3.00196 \| \| 7.69987 \| \| 2.29124 \| \| 14.8114 \| \| 6.11614 \| \| 0.20102 \| \| 6.39920 \| \| 3.15742 \| \| 1.04508 \| \| 1.20609 \| \| 0.95282 \| \| 9.51951 \| \| 2.30169 \| \| 9.02251 \| \| 3.34516 \| \| 5.14493 \| \| 0.39837 \| \| 9.50178 \| \| 0.10155 \| \| 0.14057 \| \| 0.38026 \| \| 0.71961 \| \| 0.13271 \| \| 1.21821 \| \| 0.38821 \| \| 0.60146 \| \| 5.07829 \| \| 1.85068 \| \| 24.6581 \| | \| 0.41466 \| \| --- \| \| 3.47156 \| \| 7.86903 \| \| 2.38963 \| \| 0.39208 \| \| 1.17849 \| \| 3.32929 \| \| 10.7112 \| \| 0.17906 \| \| 0.18794 \| \| 0.82216 \| \| 2.83617 \| \| 0.52777 \| \| 0.68852 \| \| 3.95055 \| \| 4.44981 \| \| 1.23917 \| \| 0.34252 \| \| 9.94905 \| \| 0.57714 \| \| 0.68152 \| \| 0.35063 \| \| 150.622 \| \| 2.38212 \| \| 16.7059 \| \| 1.73452 \| \| 0.06594 \| \| 0.35872 \| \| 20.4914 \| \| 0.01327 \| \| 1.94510 \| \| 12.3096 \| \| 2.74556 \| \| 0.42750 \| \| 2.43558 \| \| 3.72877 \| \| 1.43784 \| \| 2.66674 \| \| 12.3778 \| \| 4.63542 \| | \| 0.14906 \| \| --- \| \| 5.60633 \| \| 0.19617 \| \| 0.30654 \| \| 1.14792 \| \| 4.85666 \| \| 1.11610 \| \| 1.50855 \| \| 0.73742 \| \| 4.18230 \| \| 0.33289 \| \| 0.36072 \| \| 0.55284 \| \| 3.41497 \| \| 0.90865 \| \| 0.51570 \| \| 0.01003 \| \| 0.17683 \| \| 3.74942 \| \| 0.44691 \| \| 2.81753 \| \| 0.66443 \| \| 23.3702 \| \| 0.05746 \| \| 0.14282 \| \| 0.18301 \| \| 0.03628 \| \| 0.11052 \| \| 0.11280 \| \| 0.09587 \| \| 0.61513 \| \| 7.50557 \| \| 0.32507 \| \| 1.13365 \| \| 0.54159 \| \| 0.53961 \| \| 0.22130 \| \| 0.40690 \| \| 0.51190 \| \| 22.8462 \| |

**Table S3. Demographic and clinical characteristics of 80 BLCA patients and PKIB protein expression in 80 paired BLCA tissues.**

| Case | Age | Gender | Overall survival | Alive(0) or dead(1) | TNM | Depth of invasion | Normal | Tumor |
| --- | --- | --- | --- | --- | --- | --- | --- | --- |
| \| 1 \| \| --- \| \| 2 \| \| 3 \| \| 4 \| \| 5 \| \| 6 \| \| 7 \| \| 8 \| \| 9 \| \| 10 \| \| 11 \| \| 12 \| \| 13 \| \| 14 \| \| 15 \| \| 16 \| \| 17 \| \| 18 \| \| 19 \| \| 20 \| \| 21 \| \| 22 \| \| 23 \| \| 24 \| \| 25 \| \| 26 \| \| 27 \| \| 28 \| \| 29 \| \| 30 \| \| 31 \| \| 32 \| \| 33 \| \| 34 \| \| 35 \| \| 36 \| \| 37 \| \| 38 \| \| 39 \| \| 40 \| \| 41 \| \| 42 \| \| 43 \| \| 44 \| \| 45 \| \| 46 \| \| 47 \| \| 48 \| \| 49 \| \| 50 \| \| 51 \| \| 52 \| \| 53 \| \| 54 \| \| 55 \| \| 56 \| \| 57 \| \| 58 \| \| 59 \| \| 60 \| \| 61 \| \| 62 \| \| 63 \| \| 64 \| \| 65 \| \| 66  67  68  69  70  71  72  73  74  75  76  77  78  79  80 \| | 61  61  64  80  71  60  72  65  70  83  61  63  79  74  68  91  49  72  59  69  79  76  66  70  63  80  67  68  73  57  77  81  60  67  74  63  59  71  57  59  76  83  64  65  61  63  64  67  68  73  59  51  73  57  69  68  65  67  67  61  67  73  69  63  75  63  65  69  62  69  68  65  74  71  66  69  79  63  71  78 | \| male \| \| --- \| \| male \| \| male \| \| male \| \| male \| \| male \| \| male \| \| male \| \| female \| \| male \| \| male \| \| male \| \| male \| \| male \| \| male \| \| male \| \| male \| \| male \| \| male \| \| male \| \| male \| \| male \| \| male \| \| male \| \| male \| \| male \| \| male \| \| male \| \| male \| \| female \| \| male \| \| male \| \| male \| \| male \| \| male \| \| male \| \| male \| \| male \| \| male \| \| male \| \| male \| \| male \| \| male \| \| male \| \| male \| \| male \| \| male \| \| male \| \| male \| \| male \| \| male \| \| male \| \| male \| \| male \| \| male \| \| male \| \| male \| \| male \| \| male \| \| male \| \| male \| \| male \| \| male \| \| male \| \| male \| \| male \| \| male \| \| male \| \| male \| \| male \| \| male \| \| male \| \| male \| \| male \| \| male \| \| male \| \| male \| \| male \| \| male \| \| male \| | 3615  601  1423  800  1856  3320  2961  1361  292  3832  3185  1356  2572  2109  1772  2362  1105  1044  1922  742  3593  464  3292  746  2662  1515  3252  1311  1021  2318  1599  2670  2002  2235  820  480  2577  1193  339  2465  1159  2519  1677  1313  2411  955  724  1086  2413  908  1505  771  2096  3160  1048  3029  1406  490  3211  1386  3512  1476  1094  3519  1400  1427  2399  1339  3511  836  2396  1353  570  3225  1999  906  3287  1556  2434  933 | 0  1  1  1  1  1  0  1  1  0  0  1  0  1  1  1  1  1  1  1  0  1  0  1  0  1  0  1  1  0  1  0  1  0  1  1  0  1  1  1  1  0  1  1  0  1  1  1  0  1  1  1  1  0  1  1  1  1  0  1  0  1  1  0  1  1  0  1  0  1  0  1  1  0  0  1  0  1  1  1 | T2N0M0  T4N0M1  T2N0M0  T4N0M1  T4N0M0  T2N2M0  T3N0M0  T4N2M1  T2N1M0  T2N0M0  T1N0M0  T1N0M0  T3N0M0  T4N2M1  T4N2M1  T2N2M0  T4N0M1  T2N0M1  T3N2M0  T2N0M0  T2N0M0  T3N0M1  T1N0M0  T2N0M0  T2N1M0  T2N0M1  T2N0M0  T3N0M0  T2N0M0  T1N0M0  T3N0M0  T2N0M0  T2N0M0  T1N0M0  T3N0M0  T4N0M1  T2N0M0  T4N0M1  T4N2M1  T3N2M0  T4N2M1  T1N0M0  T3N0M0  T4N0M0  T1N0M0  T3N0M0  T3N0M1  T4N2M1  T1N0M0  T4N0M1  T4N2M1  T4N0M1  T2N2M0  T1N0M0  T2N0M0  T3N0M0  T4N2M1  T4N0M1  T1N0M0  T4N0M1  T2N0M0  T4N0M0  T4N2M1  T3N2M0  T3N0M0  T4N0M1  T1N0M0  T4N2M1  T1N0M0  T4N0M1  T4N0M0  T2N2M0  T4N2M1  T2N2M0  T2N0M0  T4N2M1  T1N0M0  T4N2M1  T4N2M1  T4N2M1 | \| MIBC \| \| --- \| \| MIBC \| \| MIBC \| \| MIBC \| \| MIBC \| \| MIBC \| \| MIBC \| \| MIBC \| \| MIBC \| \| MIBC \| \| NMIBC \| \| NMIBC \| \| MIBC \| \| MIBC \| \| MIBC \| \| MIBC \| \| MIBC MIBC \| \| MIBC \| \| MIBC \| \| MIBC \| \| MIBC \| \| NMIBC \| \| MIBC \| \| NMIBC \| \| MIBC \| \| MIBC \| \| MIBC \| \| MIBC \| \| NMIBC \| \| MIBC \| \| MIBC  MIBC  NMIBC  MIBC  MIBC  MIBC  MIBC  MIBC  MIBC  MIBC  NMIBC  MIBC  MIBC  NMIBC  MIBC  MIBC  MIBC  NMIBC  MIBC \| \| MIBC  MIBC  MIBC  NMIBC  MIBC  MIBC  MIBC  MIBC  NMIBC  MIBC  MIBC  MIBC  MIBC  MIBC  MIBC  MIBC  NMIBC  MIBC  NMIBC  MIBC  MIBC  MIBC  MIBC  MIBC  MIBC  MIBC  MIBC  MIBC  MIBC  MIBC \| | 2  1  2  2  1  2  2  2  0.5  1  1  1  1  1  1  1  1  1  5  1  1  1  1  0.5  2  1  1  0.5  2  1  1  1  1  0.5  1  1  1  0.5  1  1  1  1  1  0.5  2  1  1  0.5  1  1  1  1  0.5  1  1  0.5  0.5  1  0.5  2  1  0.5  1  0.5  0.5  0.5  1  0.5  0.5  0.5  1  0.5  0.5  0.5  1  0.5  1  0.5  2  0.5 | 1  2  2  2  3  1  1  1  3  1  1  2  1  2  1  2  1  1  0.5  1  1  2  1  1  1  2  1  1  1  1  0.5  2  2  3  3  2  3  2  2  2  2  1  3  2  1  0.5  0.5  1  1  2  1  1  1  1  3  2  1  2  1  1  1  2  2  3  1  1  1  1  1  1  1  1  0.5  1  2  1  0.5  0.5  2  1 |

**Table S4. Demographic and clinical characteristics of 97 BLCA patients and protein expression of PKIB in BLCA and normal urothelial tissues.**

| Case | Age | Gender | Grade | Tissue  source | TNM | Depth of invasion | PKIB |
| --- | --- | --- | --- | --- | --- | --- | --- |
| \| 1 \| \| --- \| \| 2 \| \| 3 \| \| 4 \| \| 5 \| \| 6 \| \| 7 \| \| 8 \| \| 9 \| \| 10 \| \| 11 \| \| 12 \| \| 13 \| \| 14 \| \| 15 \| \| 16 \| \| 17 \| \| 18 \| \| 19 \| \| 20 \| \| 21 \| \| 22 \| \| 23 \| \| 24 \| \| 25 \| \| 26 \| \| 27 \| \| 28 \| \| 29 \| \| 30 \| \| 31 \| \| 32 \| \| 33 \| \| 34 \| \| 35 \| \| 36 \| \| 37 \| \| 38 \| \| 39 \| \| 40 \| \| 41 \| \| 42 \| \| 43 \| \| 44 \| \| 45 \| \| 46 \| \| 47 \| \| 48 \| \| 49 \| \| 50 \| \| 51 \| \| 52 \| \| 53 \| \| 54 \| \| 55 \| \| 56 \| \| 57 \| \| 58 \| \| 59 \| \| 60 \| \| 61 \| \| 62 \| \| 63 \| \| 64 \| \| 65 \| \| 66  67  68  69  70  71  72  73  74  75  76  77  78  79  80  81  82  83  84  85  86  87  88  89  90  91  92  93  94  95  96  97 \| | \| 73 \| \| --- \| \| 74 \| \| 54 \| \| 81 \| \| 47 \| \| 85 \| \| 72 \| \| 56 \| \| 65 \| \| 82 \| \| 73 \| \| 60 \| \| 64 \| \| 53 \| \| 52 \| \| 56 \| \| 57 \| \| 30 \| \| 53 \| \| 61 \| \| 68 \| \| 69 \| \| 80 \| \| 86 \| \| 68 \| \| 67 \| \| 43 \| \| 64 \| \| 77 \| \| 76 \| \| 75 \| \| 63 \| \| 69 \| \| 64 \| \| 59 \| \| 69 \| \| 54 \| \| 67 \| \| 71 \| \| 73 \| \| 62 \| \| 79 \| \| 76 \| \| 55 \| \| 70 \| \| 74 \| \| 56 \| \| 86 \| \| 62 \| \| 82 \| \| 83 \| \| 72 \| \| 78 \| \| 55 \| \| 82 \| \| 63 \| \| 71 \| \| 72 \| \| 72 \| \| 85 \| \| 78 \| \| 61 \| \| 67 \| \| 73 \| \| 68 \| \| 68 \| \| 68 \| \| 60 \| \| 62 \| \| 79 \| \| 69 \| \| 79 \| \| 90 \| \| 69 \| \| 35 \| \| 82 \| \| 68 \| \| 77 \| \| 57 \| \| 90 \| \| 74 \| \| 55 \| \| 43 \| \| 55 \| \| 53 \| \| 86 \| \| 84 \| \| 81 \| \| 73 \| \| 46 \| \| 55 \| \| 66 \| \| 81 \| \| 65 \| \| 76 \| \| 78 \| \| 57 \| | \| male \| \| --- \| \| male \| \| male \| \| male \| \| male \| \| female \| \| male \| \| male \| \| male \| \| female \| \| male \| \| female \| \| male \| \| male \| \| male \| \| male \| \| male \| \| female \| \| male \| \| male \| \| male \| \| male \| \| male \| \| male \| \| male \| \| female \| \| male \| \| male \| \| male \| \| male \| \| male \| \| male \| \| male \| \| male \| \| male \| \| male \| \| female \| \| male \| \| male \| \| male \| \| male \| \| male \| \| female \| \| male \| \| male \| \| male \| \| male \| \| male \| \| male \| \| male \| \| male \| \| male \| \| male \| \| male \| \| male \| \| male \| \| male \| \| male \| \| male \| \| male \| \| male \| \| male \| \| male \| \| male \| \| male \| \| male \| \| male \| \| female \| \| female \| \| male \| \| male \| \| female \| \| female \| \| male \| \| male \| \| female \| \| male \| \| male \| \| male \| \| female \| \| female \| \| male \| \| male \| \| male \| \| male \| \| female \| \| male \| \| male \| \| male \| \| male \| \| male \| \| male \| \| female \| \| male \| \| female \| \| male \| \| male \| | \| normal \| \| --- \| \| normal \| \| normal \| \| normal \| \| normal \| \| normal \| \| normal \| \| normal \| \| normal \| \| normal \| \| normal \| \| normal \| \| normal \| \| normal \| \| normal \| \| normal \| \| normal \| \| normal \| \| normal \| \| normal \| \| normal \| \| normal \| \| low \| \| low \| \| low \| \| low \| \| low \| \| low \| \| low \| \| low \| \| low \| \| low \| \| low \| \| low \| \| low \| \| low \| \| low \| \| low \| \| low \| \| low \| \| low \| \| low \| \| low \| \| low \| \| low \| \| low \| \| low \| \| low \| \| low \| \| low \| \| high \| \| high \| \| high \| \| high \| \| high \| \| high \| \| high \| \| high \| \| high \| \| high \| \| high \| \| high \| \| high \| \| high \| \| high \| \| high \| \| high \| \| high \| \| high \| \| high \| \| high \| \| high \| \| high \| \| high \| \| high \| \| high \| \| high \| \| high \| \| high \| \| high \| \| high \| \| high \| \| high \| \| high \| \| high \| \| high \| \| high \| \| high \| \| high \| \| high \| \| high \| \| high \| \| high \| \| high \| \| high \| \| high \| \| high \| | TURBT  TURBT  TURBT  TURBT  TURBT  TURBT  TURBT  TURBT  TURBT  TURBT  TURBT  TURBT  TURBT  TURBT  TURBT  TURBT  TURBT  TURBT  TURBT  TURBT  TURBT  Radical cystectomy  TURBT  TURBT  TURBT  TURBT  TURBT  TURBT  TURBT  TURBT  Radical cystectomy  Radical cystectomy  TURBT  TURBT  Radical cystectomy  TURBT  TURBT  TURBT  TURBT  TURBT  TURBT  TURBT  TURBT  TURBT  TURBT  TURBT  TURBT  TURBT  TURBT  TURBT  TURBT  Radical cystectomy  TURBT  TURBT  TURBT  TURBT  TURBT  TURBT  TURBT  TURBT  Radical cystectomy  TURBT  Radical cystectomy  TURBT  TURBT  TURBT  TURBT  Radical cystectomy  TURBT  TURBT  Radical cystectomy  TURBT  Radical cystectomy  TURBT  Radical cystectomy  TURBT  Radical cystectomy  TURBT  TURBT  Radical cystectomy  TURBT  TURBT  TURBT  TURBT  TURBT  TURBT  Radical cystectomy  TURBT  TURBT  TURBT  TURBT  TURBT  TURBT  TURBT  Radical cystectomy  TURBT  TURBT | \| TaN0M0 \| \| --- \| \| TaN0M0 \| \| TaN0M0 \| \| TaN0M0 \| \| T1N0M0 \| \| T1N0M0 \| \| T2N0M0 \| \| TaN0M0 \| \| T1N0M0 \| \| T2N0M0 \| \| TaN0M0 \| \| T1N0M0  T1N0M0  T2N0M0  T1N0M0  T1N0M0  T1N0M0  T1N0M0  T1N0M0  T1N0M0  T1N0M0  T2N0M0  T2N0M0  T1N0M0  TaN0M0  TaN0M0  T1N0M0  TaN0M0  TaN0M0  TaN0M0  T1N0M0  T2N0M0  T1N0M0  TaN0M0  TaN0M0  T1N0M0  TaN0M0  TaN0M0  TaN0M0  TaN0M0  T1N0M0  TaN0M0  TaN0M0  TaN0M0  TaN0M0  TaN0M0  TaN0M0  TaN0M0  T1N0M0  T2N0M0  T1N0M0  T1N2M0  T1N0M0  T2N0M0  T1N0M0  T3N0M1  T1N0M0  T1N0M0  T1N0M0  T1N0M0  T4N0M1  T1N0M0  T2N0M0  T1N0M0  T1N0M0  T1N0M0  T1N0M0  T4bN0M1  T2N0M0  T1N0M0  T1N0M0  TaN0M0  TaN0M0  T2N0M0  T2N0M0  T2N0M0  T1N0M0  T1N0M0  T2N0M0  T1N0M0  T1N0M0  T1N0M0  T1N0M0  T2N0M0  T2N0M0  T1N0M0  T2aN0M0  T2N0M0  T2N0M0  T2N0M0  T1N0M0  T2N0M0  T2N0M0  T2N0M0  T1N0M0  T1N0M0  T1N0M0 \| | \| NMIBC \| \| --- \| \| NMIBC \| \| NMIBC \| \| NMIBC \| \| NMIBC \| \| MIBC \| \| MIBC \| \| NMIBC \| \| NMIBC \| \| MIBC \| \| NMIBC \| \| NMIBC \| \| NMIBC \| \| MIBC \| \| NMIBC \| \| NMIBC \| \| NMIBC NMIBC \| \| NMIBC \| \| NMIBC \| \| NMIBC \| \| MIBC \| \| NMIBC \| \| NMIBC \| \| NMIBC \| \| NMIBC \| \| NMIBC \| \| MIBC \| \| NMIBC \| \| NMIBC \| \| NMIBC \| \| MIBC  NMIBC  NMIBC  NMIBC  NMIBC  NMIBC  NMIBC  NMIBC  NMIBC  NMIBC  NMIBC  NMIBC  NMIBC  NMIBC  NMIBC  NMIBC  NMIBC  NMIBC  NMIBC \| \| NMIBC  MIBC  NMIBC  MIBC  MIBC  MIBC  MIBC  MIBC  NMIBC  MIBC  MIBC  NMIBC  MIBC  NMIBC  NMIBC  MIBC  NMIBC  MIBC  MIBC  NMIBC  NMIBC  NMIBC  NMIBC  MIBC  MIBC  MIBC  NMIBC  NMIBC  MIBC  NMIBC  NMIBC  NMIBC  NMIBC  MIBC  MIBC  NMIBC  MIBC  MIBC  MIBC  MIBC  NMIBC  MIBC  MIBC  MIBC  NMIBC  NMIBC  NMIBC \| | \| 1 \| \| --- \| \| 1 \| \| 1 \| \| 0 \| \| 0 \| \| 2 \| \| 1 \| \| 0 \| \| 1 \| \| 2 \| \| 0 \| \| 0 \| \| 0  0  1  0  1  2  0  0  3  3  3  1  3  3  3  3  1  1  1  1  2  1  0  2  2  2  1  2  0  2  0  1  1  2  1  0  3  2  3  2  3  1  2  1  3  3  3  3  2  2  3  3  2  3  1  2  2  3  2  2  0  2  2  2  0  3  2  1  2  0  2  0  1  0  1  2  3  0  2  0  1  1  3  3  1 \| |

**Table S6. Demographic and clinical characteristics of 79 BLCA patients and protein expression of** **HSP27-S15 in BLCA and normal urothelial tissues.**

| Case | Age | Gender | Grade | Tissue source | TNM | Depth of invasion | HSP27-S15 |
| --- | --- | --- | --- | --- | --- | --- | --- |
| \| 1 \| \| --- \| \| 2 \| \| 3 \| \| 4 \| \| 5 \| \| 6 \| \| 7 \| \| 8 \| \| 9 \| \| 10 \| \| 11 \| \| 12 \| \| 13 \| \| 14 \| \| 15 \| \| 16 \| \| 17 \| \| 18 \| \| 19 \| \| 20 \| \| 21 \| \| 22 \| \| 23 \| \| 24 \| \| 25 \| \| 26 \| \| 27 \| \| 28 \| \| 29 \| \| 30 \| \| 31 \| \| 32 \| \| 33 \| \| 34 \| \| 35 \| \| 36 \| \| 37 \| \| 38 \| \| 39 \| \| 40 \| \| 41 \| \| 42 \| \| 43 \| \| 44 \| \| 45 \| \| 46 \| \| 47 \| \| 48 \| \| 49 \| \| 50 \| \| 51 \| \| 52 \| \| 53 \| \| 54 \| \| 55 \| \| 56 \| \| 57 \| \| 58 \| \| 59 \| \| 60 \| \| 61 \| \| 62 \| \| 63 \| \| 64 \| \| 65 \| \| 66  67  68  69  70  71  72  73  74  75  76  77  78  79 \| | \| 52 \| \| --- \| \| 74 \| \| 56 \| \| 58 \| \| 73 \| \| 73 \| \| 74 \| \| 54 \| \| 81 \| \| 61 \| \| 68 \| \| 79 \| \| 66  65  71  54  85  69  66  82  72  66  75  72  80  61  70  64  75  81  66  64  62  73  71  81  85  72  60  69  67  52 \| \| 67  65  65  77  82  75  63  67  64  70  77  83  62  77  77  73  74  78  66  74  90  80  70  78  70  56  57  55  73  74  88  62  33  89  80  86  87 \| | \| male \| \| --- \| \| male \| \| female \| \| male \| \| male \| \| male \| \| male \| \| male \| \| male \| \| male \| \| male \| \| female \| \| female \| \| male \| \| male \| \| male \| \| female \| \| male \| \| male \| \| male \| \| male \| \| male \| \| male \| \| male \| \| male \| \| male \| \| male \| \| male \| \| male \| \| female \| \| male \| \| male \| \| male \| \| male \| \| male \| \| female \| \| male \| \| male \| \| male \| \| male \| \| male \| \| male \| \| male \| \| male \| \| male \| \| male \| \| male \| \| male \| \| male \| \| male \| \| female \| \| male \| \| female \| \| male \| \| male \| \| female \| \| male \| \| male \| \| male \| \| male \| \| male \| \| female \| \| male \| \| male \| \| female \| \| female \| \| male \| \| male \| \| male \| \| male \| \| female \| \| male \| \| female \| \| male \| \| male \| \| male \| \| male \| \| male \| \| male \| | \| normal \| \| --- \| \| normal \| \| normal \| \| normal \| \| normal \| \| normal \| \| normal \| \| normal \| \| normal \| \| normal \| \| normal \| \| normal \| \| low \| \| low \| \| low \| \| low \| \| low \| \| low \| \| low \| \| low \| \| low \| \| low \| \| low \| \| low \| \| low \| \| low \| \| low \| \| low \| \| low \| \| low \| \| low \| \| low \| \| low \| \| low \| \| low \| \| low \| \| low \| \| low \| \| low \| \| low \| \| low \| \| low \| \| high \| \| high \| \| high \| \| high \| \| high \| \| high \| \| high \| \| high \| \| high \| \| high \| \| high \| \| high \| \| high \| \| high \| \| high \| \| high \| \| high \| \| high \| \| high \| \| high \| \| high \| \| high \| \| high \| \| high \| \| high \| \| high \| \| high \| \| high \| \| high \| \| high \| \| high \| \| high \| \| high \| \| high \| \| high \| \| high \| \| high \| | TURBT  TURBT  TURBT  TURBT  TURBT  TURBT  TURBT  TURBT  TURBT  TURBT  TURBT  Radical cystectomy  TURBT  TURBT  TURBT  TURBT  TURBT  TURBT  TURBT  TURBT  TURBT  TURBT  TURBT  TURBT  TURBT  TURBT  TURBT  TURBT  TURBT  TURBT  TURBT  TURBT  TURBT  TURBT  TURBT  TURBT  TURBT  TURBT  TURBT  TURBT  TURBT  TURBT  TURBT  TURBT  TURBT  TURBT  TURBT  TURBT  TURBT  TURBT  Radical cystectomy  TURBT  TURBT  TURBT  TURBT  TURBT  TURBT  TURBT  TURBT  TURBT  TURBT  TURBT  TURBT  TURBT  TURBT  TURBT  TURBT  TURBT  TURBT  TURBT  TURBT  TURBT  Radical cystectomy  TURBT  TURBT  TURBT  Radical cystectomy  TURBT  Radical cystectomy | \| T1N0M0 \| \| --- \| \| TaN0M0 \| \| TaN0M0 \| \| TaN0M0 \| \| T1N0M0 \| \| TaN0M0 \| \| T1N0M0 \| \| TaN0M0 \| \| T1N0M0 \| \| T1N0M0 \| \| T1N0M0 \| \| T2N0M0  TaN0M0  TaN0M0  T1N0M0  TaN0M0  T1N0M0  T1N0M0  T1N0M0  T1N0M0  T1N0M0  T1N0M0  T1N0M0  T1N0M0  T1N0M0  T1N0M0  T1N0M0  T1N0M0  TaN0M0  TaN0M0  T1N0M0  T1N0M0  T1N0M0  T1N0M0  T1N0M0  TaN0M0  TaN0M0  T1N0M0  T1N0M0  TaN0M0  TaN0M0  TaN0M0 T1N0M0  T1N0M0  T1N0M0  T1N0M0  T2N0M0  T1N0M0  T1N0M0  T1N0M0  T4aN2M1  T1N0M0  T1N0M0  T1N0M0  T1N0M0  T1N0M0  T2aN0M0  T2N0M0  T1N0M0  T1N0M0  T1N0M0  T1N0M0  T1N0M0  T1N0M0  T1N0M0  T1N0M0  T1N0M0  T1N0M0  TisN0M0  T1N0M0  T1N0M0  T1N0M0  T3N0M0  T1N0M0  TaN0M0  T1N0M0  T1N1M0  TxN0M0  T1N0M0 \| | \| NMIBC \| \| --- \| \| NMIBC \| \| NMIBC \| \| NMIBC \| \| NMIBC \| \| NMIBC \| \| NMIBC \| \| NMIBC \| \| NMIBC \| \| NMIBC \| \| NMIBC \| \| MIBC \| \| NMIBC \| \| NMIBC \| \| NMIBC \| \| NMIBC \| \| NMIBC \| \| NMIBC \| \| NMIBC \| \| NMIBC \| \| NMIBC \| \| NMIBC \| \| NMIBC \| \| NMIBC \| \| NMIBC \| \| NMIBC \| \| NMIBC \| \| NMIBC \| \| NMIBC \| \| NMIBC \| \| NMIBC \| \| NMIBC \| \| NMIBC \| \| NMIBC \| \| NMIBC \| \| NMIBC \| \| NMIBC \| \| NMIBC \| \| NMIBC \| \| NMIBC \| \| NMIBC \| \| NMIBC  NMIBC  NMIBC  NMIBC  NMIBC  MIBC  NMIBC  MIBC  NMIBC  MIBC  NMIBC  NMIBC  MIBC  MIBC  NMIBC  NMIBC  MIBC  NMIBC  NMIBC  MIBC  NMIBC  NMIBC  NMIBC  NMIBC  NMIBC  NMIBC  NMIBC  NMIBC  NMIBC  NMIBC  NMIBC  MIBC  NMIBC  NMIBC  NMIBC  MIBC  NMIBC  NMIBC \| | \| 1 \| \| --- \| \| 3 \| \| 3 \| \| 3 \| \| 2 \| \| 3 \| \| 2 \| \| 3 \| \| 3 \| \| 2 \| \| 3 \| \| 3 \| \| 3  3  2  3  2  3  2  3  2  2  3  3  2  2  1  3  3  3  0  3  3  3  3  2  3  2  3  2  2  1  3  2  3  3  0  1  0  1  0  3  3  1  1  1  3  0  3  1  2  2  2  0  2  3  3  1  0  1  3  2  0  1  2  0  2  3  1 \| |
