# Supplementary material for: PKIB facilitates bladder cancer proliferation and metastasis through mediation of HSP27 phosphorylation by PKA
Source: Cell Death Dis. 2025 Jul 1;16(1):470. doi: 10.1038/s41419-025-07814-7 (PMC12219054; doi:10.1038/s41419-025-07814-7)
Supplement: Supplementary file 5 — Supplementary original Western blots [file 41419_2025_7814_MOESM5_ESM.docx]

**Figure 2**







Figure 2.A T24 β-actin Figure 2.A T24 PKIB







Figure 2.A 5637 β-actin Figure 2.A 5637 PKIB







Figure 2.E T24 β-actin Figure 2.E T24 N-cadherin







Figure 2.E T24 Vimentin Figure 2.E 5637 β-actin







Figure 2.E 5637 N-cadherin Figure 2.E 5637 Vimentin

**Figure 3**







Figure 3.A T24 PKA Figure 3.A T24 β-actin







Figure 3.B Cytoplasm β-actin Figure 3.B Cytoplasm LMNB1







Figure 3.B Cytoplasm PKA Figure 3.B Nucleus β-actin







Figure 3.B Nucleus LMNB1 Figure 3.B Nucleus PKA







Figure 3.D PKA Figure 3.E IP:FLAG IB:HA







Figure 3.E IP:FLAG IB:FLAG Figure 3.E(Left) INPUT IB:FLAG







Figure 3.E(Left) INPUT IB:HA Figure 3.E IP:HA IB:HA







Figure 3.E IP:HA IB:FLAG Figure 3.E(Right) INPUT IB:FLAG







Figure 3.E(Right) INPUT IB:HA Figure 3.F(Left) IP IB:HSP27







Figure 3.F(Left) IP IB:PKA Figure 3.F(Left) INPUT IB:PKA







Figure 3.F(Left) INPUT IB:HSP27 Figure 3.F(Right) IP IB:PKA







Figure 3.F(Right) IP IB:HSP27 Figure 3.F(Right) INPUT IB:PKA







Figure 3.F(Right) INPUT IB:HSP27 Figure 3.H INPUT IB:FLAG







Figure 3.H INPUT IB:HA Figure 3.H IP IB:FLAG







Figure 3.H IP IB:HA Figure 3.K HSP27 S78







Figure 3.K HSP27 S15 Figure 3.K HSP27 S82







Figure 3.K HSP27 Figure 3.K β actin







Figure 3.L HSP27 S78 Figure 3.L HSP27 S82







Figure 3.L HSP27 S15 Figure 3.L PKA







Figure 3.L HSP27 Figure 3.L β actin







Figure 3.M HSP27 S15 Figure 3.M HSP27 S78







Figure 3.M HSP27 S82 Figure 3.M HSP27







Figure 3.M PKA Figure 3.N p-Ser







Figure 3.N GST Figure 3.N PKA







Figure 3.O INPUT IB: HA Figure 3. O INPUT IB: FLAG







Figure 3.O IP IB: p-Ser Figure 3. O IP IB: FLAG







Figure 3. O IP IB: HA Figure 3.P HSP27 S15







Figure 3.P HSP27 S78 Figure 3.P HSP27 S82







Figure 3.P HSP27 Figure 3.P β-actin

**Figure 4**







Figure 4.A N-cadherin Figure 4.A Vimentin







Figure 4.A HSP27 Figure 4.A β-actin







Figure 4.I N-cadherin Figure 4.I Vimentin







Figure 4.I HSP27 Figure 4.I β-actin





Figure 4.I PKIB

**Figure 5**







Figure 5.A HSP27 Figure 5.A β-actin







Figure 5.C N-cadherin Figure 5.C Vimentin





Figure 5.C β-actin







Figure 5.G β-actin Figure 5.G AKT







Figure 5.G p-AKT Figure 5.I β-actin







Figure 5.I AKT Figure 5.I p-AKT

**Figure 6**







Figure 6.A MYCN Figure 6.A β-actin





Figure 6.A PKIB

**Figure 7**







Figure 7.K T24 β-actin Figure 7.K T24 HSP27







Figure 7.K T24 HSP27 S15 Figure 7.K T24 HSP27 S78







Figure 7.K T24 HSP27 S82 Figure 7.K T24 PKIB





Figure 7.K T24 MYCN







Figure 7.K 5637 β-actin Figure 7.K 5637 HSP27

Figure 7.K 5637 HSP27 S15 Figure 7.K 5637 HSP27 S78

Figure 7.K 5637 HSP27 S82 Figure 7.K 5637 PKIB

Figure 7.K 5637 MYCN

**Figure S1**

Figure S1.A N-cadherin Figure S1.A Vimentin

Figure S1.A β-actin Figure S1.A PKIB

**Figure S2**

Figure S2.A T24 N-cadherin Figure S2.A T24 Vimentin

Figure S2.A T24 β-actin Figure S2.A T24 PKIB

Figure S2.A 5637 N-cadherin Figure S2.A 5637 Vimentin

Figure S2.A 5637 β-actin Figure S2.A 5637 PKIB

**Figure S3**

Figure S3 C HSP27 S15 Figure S3 C HSP27 S78

Figure S3 C HSP27 S82 Figure S3 C HSP27

Figure S3 C β-actin

Figure S3 D HSP27 S15 Figure S3 D HSP27 S78

Figure S3 D HSP27 S82 Figure S3 D HSP27

Figure S3 D β-actin
